# Therapeutic potential of BOLD-100, a GRP78 inhibitor, enhanced by ATR inhibition in pancreatic ductal adenocarcinoma

**DOI:** 10.1186/s12964-025-02242-8

**Published:** 2025-06-13

**Authors:** Su In Lee, Ah-Rong Nam, Kyoung-Seok Oh, Jae-Min Kim, Ju-Hee Bang, Yoojin Jeong, Sea Young Choo, Hyo Jung Kim, Jeesun Yoon, Tae-Yong Kim, Do-Youn Oh

**Affiliations:** 1 Cancer Research Institute, Seoul National University College of Medicine, 101 Daehak-Ro, Jongno-Gu, Seoul, 03080 Korea; 2 Integrated Major in Innovative Medical Science, Seoul National University Graduate School, Seoul, 03080 Korea; 3 Department of Internal Medicine, Seoul National University Hospital, Seoul, 03080 Korea

**Keywords:** GRP78, ER stress, CHOP, ROS, DNA damage response, ATR, Pancreatic cancer

## Abstract

Pancreatic ductal adenocarcinoma (PDAC) is characterized by poor prognosis and resistance to conventional therapies, necessitating novel treatments. The high proliferative rate and protein synthesis in PDAC induce endoplasmic reticulum (ER) stress, with Glucose-Regulated Protein 78 (GRP78), a key regulator of ER stress and the Unfolded Protein Response (UPR), playing a pivotal role in PDAC progression. Despite its relevance, GRP78-targeted therapies remain unexplored in PDAC. BOLD-100, a novel GRP78 inhibitor, presents a potential therapeutic approach by disrupting GRP78 transcription, though its effects on PDAC have yet to be fully elucidated. Here, we found that BOLD-100 induces PDAC cell death through the UPR pathway activation, leading to CHOP-dependent apoptosis. BOLD-100 generates reactive oxygen species (ROS), inducing R-loop formation that triggers a DNA damage response via the ATR/Chk1 axis. BOLD-100 synergizes with AZD6738, an ATR inhibitor, to enhance anti-tumor efficacy compared to either agent alone in both in vitro and in vivo models. These findings suggest that BOLD-100, especially in combination with an ATR inhibitor, represents a promising therapeutic option for patients with PDAC.

**Supplementary Information:**

The online version contains supplementary material available at 10.1186/s12964-025-02242-8.

## Introduction

Pancreatic cancer, with a 5-year survival rate under 10%, is among the most lethal malignancies [1]. Pancreatic ductal adenocarcinoma (PDAC), which constitutes 90% of pancreatic cancers, is characterized by aggressive progression, late diagnosis, and resistance to standard treatments. Although surgical resection and adjuvant chemotherapy offers a potential therapeutic benefit, they are feasible in only 10–20% of patients with PDAC, underscoring the need for novel therapies [2, 3].

Endoplasmic reticulum (ER) stress supports tumor adaptation and survival, thereby emerging as a therapeutic target in PDAC [4–6]. Glucose-Regulated Protein 78 (GRP78) is a key mediator of the Unfolded Protein Response (UPR), regulates the balance between ER’s protein folding capacity and the load of misfolded or unfolded proteins, enabling cellular adaptation to stress [7–9]. Under normal conditions, GRP78 binds to and inactivates key UPR mediators (IRE1, PERK, ATF6) to maintain homeostasis. During ER stress, GRP78 dissociates from these mediators, including PERK, triggering its auto-phosphorylation and dimerization, which activates downstream molecules such as p-eIF2α, ATF4, and CHOP to manage misfolded proteins, activating the UPR to restore balance. However, when ER stress exceeds a critical threshold, prolonged UPR activation leads to apoptosis [10–13]. This dual role of GRP78, enabling tumor survival under manageable stress but promoting apoptosis under severe stress, highlights its potential as a therapeutic target. In preclinical mouse models, a 50% reduction in GRP78 expression has been shown to significantly impede tumor growth while sparing normal cells [14, 15]. The reduction of stress-induced GRP78 also enhances apoptosis and suppresses processes like angiogenesis, invasion, and metastasis [16, 17]. Aligned with its role in regulating cellular stress responses, elevated GRP78 levels in tumor tissues highlight its contribution to tumor progression [18]. These findings collectively position GRP78 as a promising target for PDAC treatment, although its therapeutic potential in this context remains underexplored.

ER stress-induced disruption of disulfide bond formation, essential for protein folding, is linked to reactive oxygen species (ROS) accumulation and apoptosis [19–22]. ROS-mediated oxidative stress induces DNA damage and replication stress, generating R-loops that activate DNA damage responses (DDR) for repair and stress tolerance [23, 24]. Cancer cells exploit these pathways to survive, making key DDR components promising targets to enhance the anti-tumor effects of ER stress inducers such as GRP78 inhibitors.

BOLD-100, sodium trans-[tetrachlorobis(1H-indazole) ruthenate(III)], is an intravenously administered small molecule compound that downregulates GRP78 expression by inhibiting its mRNA transcription across various cancer types [25–28]. Despite its promising potential in these solid tumors, the therapeutic efficacy of BOLD-100 in pancreatic cancer remains under investigation, with an ongoing clinical trial currently evaluating its effects (NCT04421820), although no results have been publicly released. In this study, we hypothesized that inhibiting GRP78 expression induces ER stress, increases ROS production, and activates the DDR. We aim to explore the anti-tumor effects of BOLD-100 and investigate its potential synergy with DDR-targeting agents.

## Methods

### Human cell lines and reagents

9 human PDAC cell lines were utilized in this study. SNU-213, SNU-324, SNU-2918, PANC-1, Capan-1, Capan-2, AsPC-1, and MIA PaCa2 cells were purchased from the Korean Cell Line Bank (Seoul, Korea). HPAF-II cell was purchased from the American Type Culture Collection (Manassas, United States). PANC-1 and MIA PaCa2 were cultured in DMEM medium (Welgen Inc., Gyeongsan, Korea) containing 10% fetal bovine serum and 10 μg/mL gentamicin at 37 °C under 5% CO2. Other cells were cultured in RPMI1640 medium (Welgen Inc., Gyeongsan, Korea) containing 10% fetal bovine serum and 10 μg/mL gentamicin at 37 °C under 5% CO2. N-Acetyl-L-cysteine (NAC; ROS scavenger) and 2’, 7’-Dichlorodihydrofluorescein diacetates (DCF-DA) were purchased from Sigma-Aldrich (Burlington, Massachusetts, United States). Tauroursodeoxycholic Acid (TUDCA; ER stress inhibitor) was purchased from Merck Millipore (Burlington, Massachusetts, United States). BOLD-100 (GRP78 inhibitor) was kindly provided by Bold Therapeutics Inc. (Vancouver, British Columbia, Canada). AZD6738 (ATR inhibitor) was purchased from Selleck Chemical (Texas, Houston, United States). RNase H was purchased from Thermo Fisher Scientific (Waltham, United Staes).

### Cell viability assay

Cells were seeded in 96-well plates and incubated overnight at 37 °C. The cells were then exposed to increasing concentrations of BOLD-100 alone or in combination with AZD6738 for 3 days. Subsequently, 50 μl of 3-(4,5-dimethylthiazol-2yl)−2,5-diphenyltetrazolium bromide (MTT) solution (TGI) was added to each well, and plates were incubated at 37 °C for 4 h. The medium was removed, and 150 μl of dimethyl sulfoxide was added to each well. Cell viability was measured at 540 nm using a VersaMax Microplate Reader (Molecular Devices, Sunnyvale, CA). The experiments were performed in triplicate.

### Colony-forming assay

Single-cell suspensions were prepared using a syringe. The cells were then seeded in 6-well plates and exposed to certain drugs. Following a 10-day incubation period, the colonies were stained with Coomassie blue for 3 h and counted using CellCounter software (Nghia Ho). Each experiment was repeated three times.

### Cell cycle analysis

Cells were seeded in 60-mm dishes and treated with certain drugs. Subsequently, the cells were harvested and fixed with 70% ethanol at − 20 °C. After 2 days, 7 μl of RNase A (20 mg/mL, Invitrogen, Carlsbad, CA) was added to each well and incubated for 10 min at 37 °C. The cells were then stained with 13 μl of propidium iodide (Sigma-Aldrich) and analyzed using a FACS Calibur flow cytometer (BD Biosciences, San Jose, CA). Each experiment was conducted three times.

### Apoptosis analysis

The FITC Annexin V Apoptosis Detection Kit from BD Biosciences was utilized to identify apoptotic cells. After harvesting the cells, they were suspended in 100 μl of 1X annexin-binding buffer and then incubated in the dark at room temperature for 15 min with annexin-v-phycoerythrin and propidium iodide. Flow cytometry analysis was conducted using a FACS Calibur flow cytometer from BD Biosciences in San Jose, CA. Each experimental run was replicated three times, and a minimum of 10,000 cells were collected in each run.

### Real-time quantitative polymerase chain reaction

Whole-cell RNA extraction was performed using TriZol (Invitrogen), followed by reverse transcription with the ImProm-II Reverse Transcription System (Progmega) following the manufacturer's instructions. The resulting cDNA served as a template for real-time PCR, employing SYBR Green qPCR PreMIX for the reaction, and amplification was monitored on an ABI Prism 7300 instrument (Applied Biosystems). The thermal cycling conditions included an initial cycle at 95 °C for 10 min, followed by 41 cycles at 95 °C for 10 s, 60 °C for 15 s, and 72 °C for 30 s. Sequence information of the primers used was as follows: GRP78, sense 5′-CAT CAC GCC GTC CTA TGT CG- 3′, anti-sense 5′-CGT CAA AGA CCG TGT TCT CG −3′; β-actin: sense 5′-CCA ACC GCG AGA AGA TGA-3′, anti-sense 5′-CCA GAG GCG TAC AGG GAT AG-3′.

### Western blotting

Cells were seeded in 60-mm dishes and treated with specific drugs. Subsequently, the cells were harvested and lysed in RIPA buffer containing protease inhibitors on ice for 20 min. The proteins were then extracted, and equal amounts were used for western blot analyses, determined by the BCA assay (Thermo Fisher Scientific). The proteins were separated using sodium dodecyl sulfate–polyacrylamide gel electrophoresis (SDS-PAGE), and the separated proteins were transferred onto a Nitrocellulose membrane. After blocking the blotted membrane with 5% non-fat milk in Tris-buffered saline with Tween for 1 h, the membrane was incubated overnight at 4 °C with the following primary antibodies:

Anti-phospho-histone H2 A.X (Ser139) (#05–636, Sigma-Aldrich; 1:1000), p-ATR (Thr1989) (#GTX128145, GeneTex; 1:1000), Santa Cruz Biotechnology; GAPDH (#sc-25778, 1:2000), GRP78 (#sc-13539, 1:1000), Cell Signaling Technology; ATR (#cst-2790, 1:1000), Chk1 (#cst-2360, 1:1000), p-Chk1 (Ser 345) (#cst-2341, 1:1000), Caspase-7 (#cst-9492, 1:1000), Caspase-3 (#cst-9662, 1:1000), cyclinB1 (#cst-12231, 1:1000), p21 (#cst-2947, 1:1000), CHOP (#cst-2895, 1:1000), eIF2α(#cst-9722, 1:1000), p-eIF2α(#cst-9721, 1:1000), PERK (#cst-3192, 1:1000), ATF-4 (D4B8) (#cst-11815, 1:1000), ATF-6 (#cst-65880, 1:1000), GADD34 (#cst-41222, 1:1000), Histone H3 (#cst-9715, 1:1000), acetyl-histone-h3 (H3 K9ac) (#cst-9649, 1:1000), beta-actin (#cst-3700, 1:1000), p38α MAPK (#cst-9218, 1:1000), p-p38 (#cst-4511, 1:1000), SAPK/JNK (#cst-9252, 1:1000), phospho-SAPK/JNK (Thr183/Tyr185) (#cst-9251, 1:1000). The membrane was then incubated with HRP-conjugated secondary antibodies (Thermo Fisher Scientific), and protein bands were visualized using ECL detection.

### Immunoprecipitation

Cells were seeded in 60-mm dishes and treated with specific drugs. Subsequently, the cells were harvested and lysed in NP40 buffer containing protease inhibitors on RT for 30 min. The proteins were then extracted, and equal amounts determined by the BCA assay (Thermo Fisher Scientific). Proteins were labeled with PERK (#cst-3192) antibody or IgG rabbit (#ab37415) at 4 °C rotor overnight. Samples were incubated with Agarose A/G bead for 1 h at 4 °C rotor. The beads were then washed with NP40 buffer three times and analyzed by western blotting.

### TPE-MI staining assay

Cells were rinsed with PBS prior to staining and then treated with freshly diluted tetraphenylethene maleimide (TPE-MI) at a concentration of 50 μM for 30 min at 37 °C. TPE-MI is a non-fluorescent dye that fluoresces upon binding to free thiols in unfolded proteins, allowing detection of protein unfolding. For flow cytometry, cells were resuspended in PBS, pelleted by centrifugation (1300 rpm for 5 min), and then resuspended in 200 μl of PBS in flow cytometry tubes. Among singlet cell populations, TPE-MI positive cell percentage and mean fluorescence intensity of TPE-MI were measured using flow cytometry. Each experiment was performed in triplicate.

### siRNA interference

Specific siRNAs targeting CHOP and scrambled siRNA as negative control were purchased from Genolution (Seoul, Korea). Cells were transfected with 50 nM siRNA and incubated for 24 h. The transfected cells were harvested and re-seeded for further experiments. Sequence information of the siRNAs was as follows: CHOP #1; sense 5′- GAG UCA UUG CCU UUC UCC UUU −3′, anti-sense 5′- AGG AGA AAG GCA AUG ACU CUU −3′, CHOP #4; sense 5′- GCU AGC UGA AGA GAA UGA AUU −3′, anti-sense 5′- UUC AUU CUC UUC AGC UAG CUU −3′, Negative Control: sense 5′- CCU CGU GCC GUU CCA UCA GGU AGU U −3′, anti-sense 5′- CUA CCU GAU GGA ACG GCA CGA GGU U −3′.

### Measurements of cellular ROS

Cells were washed with serum-free media and harvested in PBS. Then, cells were stained with 25 μM DCF-DA for 30 min at 37 °C and 5% CO2 in the dark. After staining, samples were washed with ice-cold PBS and centrifuged for 5 min at 1300 rpm. Flow cytometry analysis was conducted using a FACS Calibur flow cytometer (BD Biosciences, San Jose, CA), collecting a minimum of 10,000 cells in each experiment, which was repeated three times.

### Immunofluorescence

Following exposure to specific drugs, cells underwent fixation with 4% paraformaldehyde (Biosesang, Gyeonggi-do, Korea) for 15 min and subsequent permeabilization with 0.5% Triton X-100 (Sigma-Aldrich) for 15 min. Then, cells incubated with 2% BSA in Tris-buffered saline with Tween for 1 h at room temperature. The immunostaining process involved primary antibodies, including anti-p-histone H2 AX (γ-H2 AX) (Sigma-Aldrich, #05–636; 1:300), anti-p-ATR (Thr1989) (GeneTex, #GTX128145; 1:100), and anti-S9.6 (kerafast, #ENH001). Following an overnight incubation at 4 °C with the primary antibodies, cells were stained with secondary antibodies for 1 h at room temperature: Alexa Fluor 488 goat anti-rabbit IgG (Invitrogen, #A-11008; 1:100) and Alexa Fluor 594 goat anti-mouse IgG (Invitrogen, #A-11032; 1:100–300). DAPI solution (Sigma-Aldrich) was used for nuclear staining, and fixed cells were imaged using either a STELLARIS 5 (Leica Microsystems) or Zeiss LSM 800 (Carl Zeiss) confocal laser scanning microscope.

### Histone Isolation

The Histone Extraction Kit (#ab113476) from Abcam was used. Cells were harvested and pelleted by centrifugation at 1000 rpm for 5 min at 4 °C. The cells were then resuspended in 1X Pre-Lysis Buffer and lysed on ice for 10 min. Afterward, the lysate was centrifuged at 3000 rpm for 5 min at 4 °C, and the supernatant was removed. The cell pellet was resuspended in three volumes of Lysis Buffer and incubated on ice for 30 min. Following this, the mixture was centrifuged at 12,000 rpm for 5 min at 4 °C, and the supernatant fraction was transferred. Next, 0.3 volumes of Balance-DTT Buffer were added to the supernatant immediately. Finally, the protein concentration was quantified with an OD reading, using BSA as a standard.

### In vivo xenograft experiments

Animal experiments were performed at the Institute for Experimental Animals, Seoul National University (Seoul, Korea) according to institutional guidelines, and prior approval of the study protocol was obtained from the Institutional Animal Care and Use Committee (SNU-220817–1-1). Four-week-old female athymic nude mice were purchased from Orient Bio Inc. (Seongnam, Korea). Capan-1 xenograft model mice were established via subcutaneous inoculation of 9 × 10^6^ cells in 100 μl of phosphate buffered saline. When the tumor volume reached 200 mm^3^, the mice were randomly assigned to treatment groups while ensuring that the mean tumor volume was comparable between groups. BOLD-100 (50, 75 mg/kg) were treated once a week for 4 weeks by Intraperitoneal (IP) injection. AZD6738 (25 mg/kg) were administered orally once a day for 4 weeks (5 days on/2 days off). Body weights and tumor sizes were measured every other day. The tumor volume was calculated using the following formula: tumor volume = [(width)^2^ × height]/2. For immunohistochemical analysis, tumor tissues were fixed in 4% paraformaldehyde, embedded in paraffin, and sectioned. The sections were deparaffinized, rehydrated, and subjected to antigen retrieval using a heat-induced epitope retrieval method. After antigen retrieval, slides were stained by incubation with anti-Ki-67 (#MA5-14,520, Invitrogen), GRP78 (#sc-13539, Santa Cruz), CHOP (#NBP2-49,550, Novus), p-ATR (#cst-30632), gamma H2 AX (p Ser139) (#NB100-384, Novus). Each marker was detected using the OptiView DAB IHC Detection Kit (Venetana, #760–700). H-Score was calculated using image analyzer-assisted interpretation to assess the extent of the immunoreactivity in viable tumor regions, excluding necrotic areas. All procedures were performed according to the manufacturer's instructions.

### Statistical analysis

Data analyses were performed using GraphPad Prism, version 8.0. All statistical tests were two-sided student’s t-test unless specifically mentioned on figure legends. Statistical significance was set at *p* < 0.05. The half-maximal inhibitory concentration (IC50) of agents was also analyzed using SigmaPlot software. Combined drug effects were analyzed by calculating the combination index (CI) using CalcuSyn software (Biosoft, Cambridge, UK). CIs of < 1, 1, and > 1 indicate synergistic, additive, and antagonistic effects, respectively.

## Results

### BOLD-100 leads to growth inhibition and induces cell apoptosis in pancreatic cancer cells

**Fig. 1:**
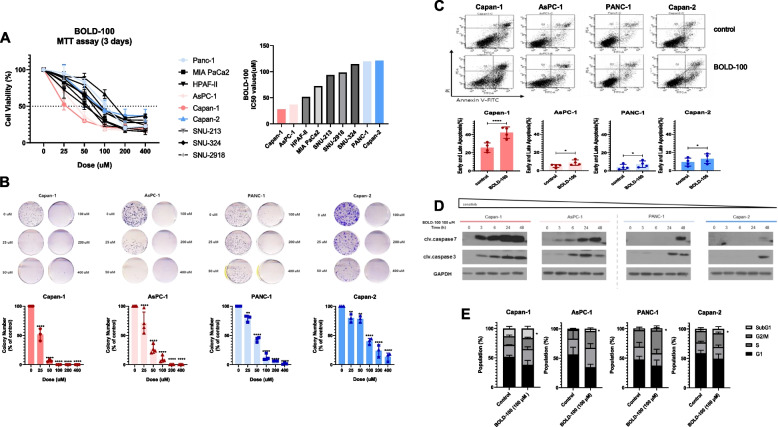
BOLD-100 leads to growth inhibition and induces cell apoptosis in pancreatic cancer cells. **A** Using MTT assays, anti-proliferative effects of BOLD-100 was evaluated in 9 human PC cell lines: Capan-1, AsPC-1, HPAF-II, MIA PaCa2, SUN-213, SNU-324, SNU-2918, PANC-1, and Capan-2. **B** Colony formation assays demonstrated the cytotoxic effect of BOLD-100. **C** FACS-based evaluation of apoptosis rates incubated with 100 μM BOLD-100 for 24 h. Data from three independent experiments are presented as mean ± SD, ns: not significant, *, *p <*0.05; ****, *p <*0.0001. **D** Protein expression levels of cleaved Caspase 7 and cleaved Caspase 3 in the cells treated with 100 μM BOLD-100. GAPDH was used as the loading control. **E** Cell cycle analysis was performed in PDAC cells after 100 μM BOLD-100 treatment for 24 h. The data of three independent experiments are depicted as mean ± SD ∗, *p <*0.05

To assess the antiproliferative impact of BOLD-100, the MTT assay was performed after treatment with BOLD-100 using a panel of 9 PDAC cell lines. It revealed a dose-dependent reduction in cell viability (Fig. 1A). We selected representative four cell lines by their IC50 values to investigate the mechanisms of action of BOLD-100: Capan-1 and AsPC-1 were relatively more sensitive, while PANC-1 and Capan-2 were relatively less sensitive. BOLD-100 treatment also significantly suppressed the colony formation of four PDAC cell lines (Fig. 1B). Further analysis using Annexin V/Dead Cell Apoptosis assay (Fig. 1C) and Western blot targeting cleaved Caspase 3 and cleaved Caspase 7 (Fig. 1D) corroborated the induction of cell apoptosis. Additionally, cell cycle analysis demonstrated an increase in the subG1 phase cell population upon BOLD-100 treatment (Fig. 1E). These collective findings confirm the antiproliferative effects of BOLD-100 with the induction of apoptosis.

### BOLD-100 induces prolonged ER stress, activating the UPR pathway and causing CHOP-dependent apoptosis

**Fig. 2:**
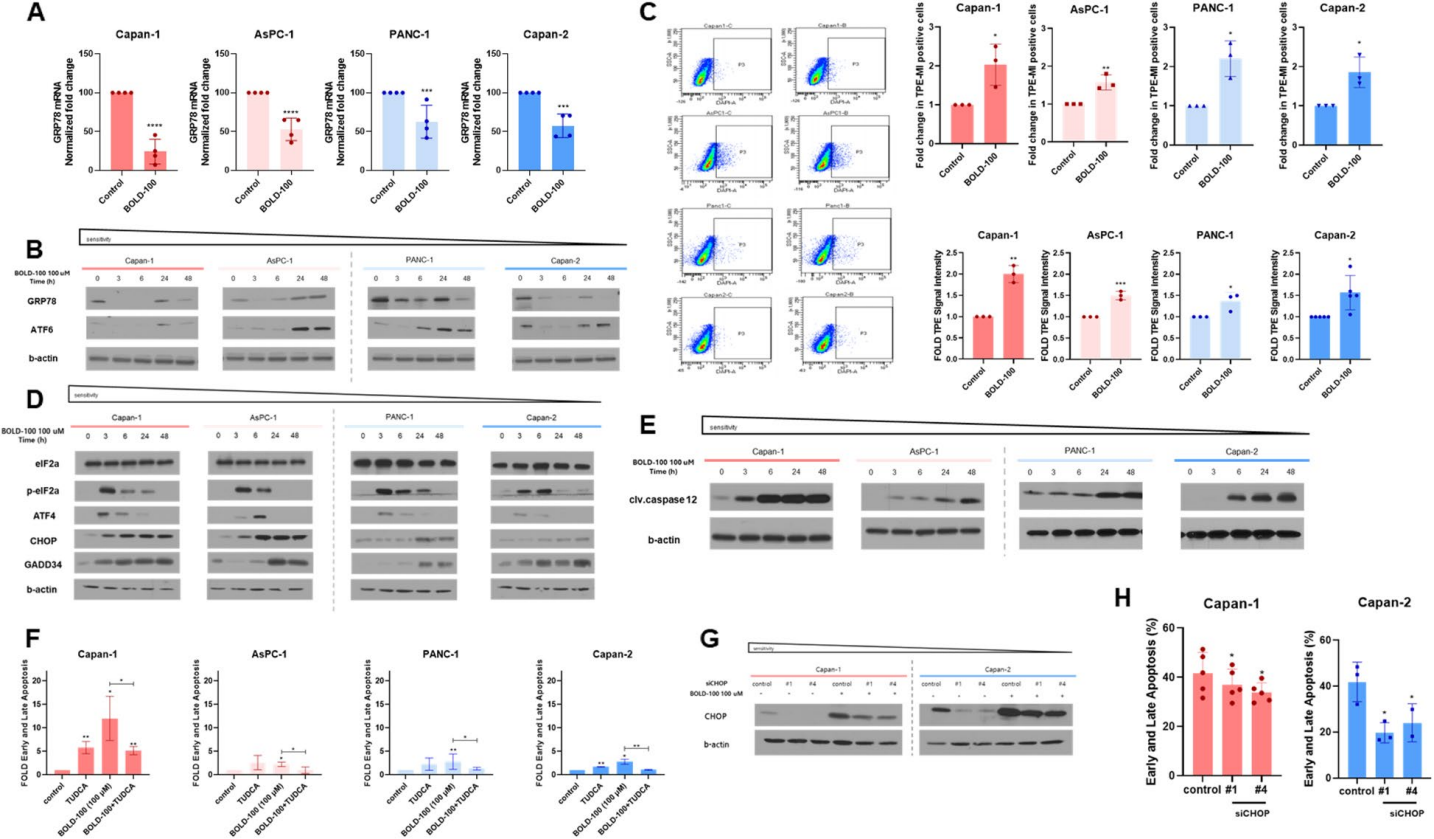
Reduction of GRP78 promotes the UPR pathway and CHOP-dependent apoptosis in PDAC cells. **A** The mRNA expression level of GRP78 in PDAC cells was determined by reverse transcription-polymerase chain reaction (qRT-PCR) after treatment with 100 μM BOLD-100 treatment for 6 h. Quantified GRP78 transcripts were normalized to b-actin, and the data of three biological replicative experiments are shown as mean ± SD ∗, *p <* 0.05; ∗∗, *p <* 0.005; ***, *p <* 0.001. **B** The whole-cell lysate was prepared and immunoblotted to determine the expression level of GRP78 and ATF-6.** C** Representative flow cytometry dot plots of TPE-high population in PDAC cells. Quantitative analysis of TPE signal intensity of each cell preparation. Cells were treated with 100 μM BOLD-100 for 6 h. Three independent experiments were conducted, and the data are shown as mean ± SEM *, *p <* 0.05. **D** Cells were treated with BOLD-100 for the indicated period and subjected to immunoblotting.Western blot analysis indicated that BOLD-100 activated the downstream signaling pathways of UPR. b-Actin was used as the loading control. **E** The protein level of cleaved Caspase 12 in the cell treated with 100 μM BOLD-100. **F** FACS-based evaluation of apoptosis rates. PDAC cells were incubated with BOLD-100 (100 µM) 24 h and TUDCA (100 µM) 24 h. Values were calculated based on three or more independent experiments and presented as mean ± SD ∗, *p* < 0.05; ∗∗, *p* < 0.005. **G** Protein expression levels of CHOP in siCont and siCHOP-transfected cells. (H) FACS-based evaluation of apoptosis rates in si-transfected cells treated with 100 µM BOLD-100. Values were calculated based on three independent experiments and presented as mean ± SD ∗, *p* < 0.05; ∗∗, *p* < 0.005; ***, *p* < 0.001

Next, we investigated the mechanism underlying the anti-tumor effect of BOLD-100. qPCR analysis revealed a significant decrease in the mRNA levels of GRP78 following BOLD-100 treatment (Fig. 2A). We also confirmed a reduction in GRP78 protein levels and dissociation between GRP78 and PERK up to 6 h treatment in PDAC cells (Fig. 2B, Fig S1 A). Interestingly, we observed the evident rebound of GRP78 expression, implying a potential UPR feedback loop mediated by ATF-6. During ER stress, GRP78 dissociates from ATF6, allowing it to translocate to the Golgi via its Golgi localization signals, where it becomes activated and subsequently upregulates GRP78 expression to restore ER homeostasis (Fig. 2B) [29]. GRP78 is an essential ER chaperone responsible for the degradation of misfolded proteins, and its reduction leads to the accumulation of unfolded proteins [30]. We utilized TPE-MI labeling, a thiol probe for measuring unfolded protein load in cells [31]. Our findings revealed elevated TPE-MI signal intensity in BOLD-100-treated PDAC cells compared to untreated cells (Fig. 2C). This accumulation of unfolded proteins induced more severe ER stress, leading to activation of the UPR pathway, as indicated by increased p-eIF2α, ATF-4, and CHOP levels (Fig. 2D). Notably, CHOP upregulates GADD34, which inhibits eIF2α phosphorylation, suggesting a negative feedback loop harnessed by cancer cells to restore homeostasis (Fig. 2D). However, continuous protein translation during prolonged ER stress can overwhelm the folding capacity of ERs and potentially leading to cell apoptosis [4]. We observed a time-dependent increase of Caspase 12 cleavage following BOLD-100, which is a specific molecular event in cells undergoing ER stress-induced apoptosis (Fig. 2E). To validate that ER stress is the main mechanism of cancer cell death by BOLD-100, we used Tauroursodeoxycholic Acid (TUDCA), an ER stress inhibitor (Fig S1B). We observed that TUDCA reduced elevated apoptosis levels induced by BOLD-100 (Fig. 2F). SiRNA-mediated silencing of CHOP, a UPR specific apoptosis factor, in Capan-1 and Capan-2 cells reversed the BOLD-100-induced anti-proliferation and apoptosis (Fig S1 C, Fig. 2G, H). These data indicate that BOLD-100 triggers ER stress- and CHOP-dependent apoptosis through the activation of the UPR pathway following GRP78 reduction.

### ER stress-induced reactive oxygen species (ROS) is implicated in the cell death by p38/JNK pathway

**Fig. 3:**
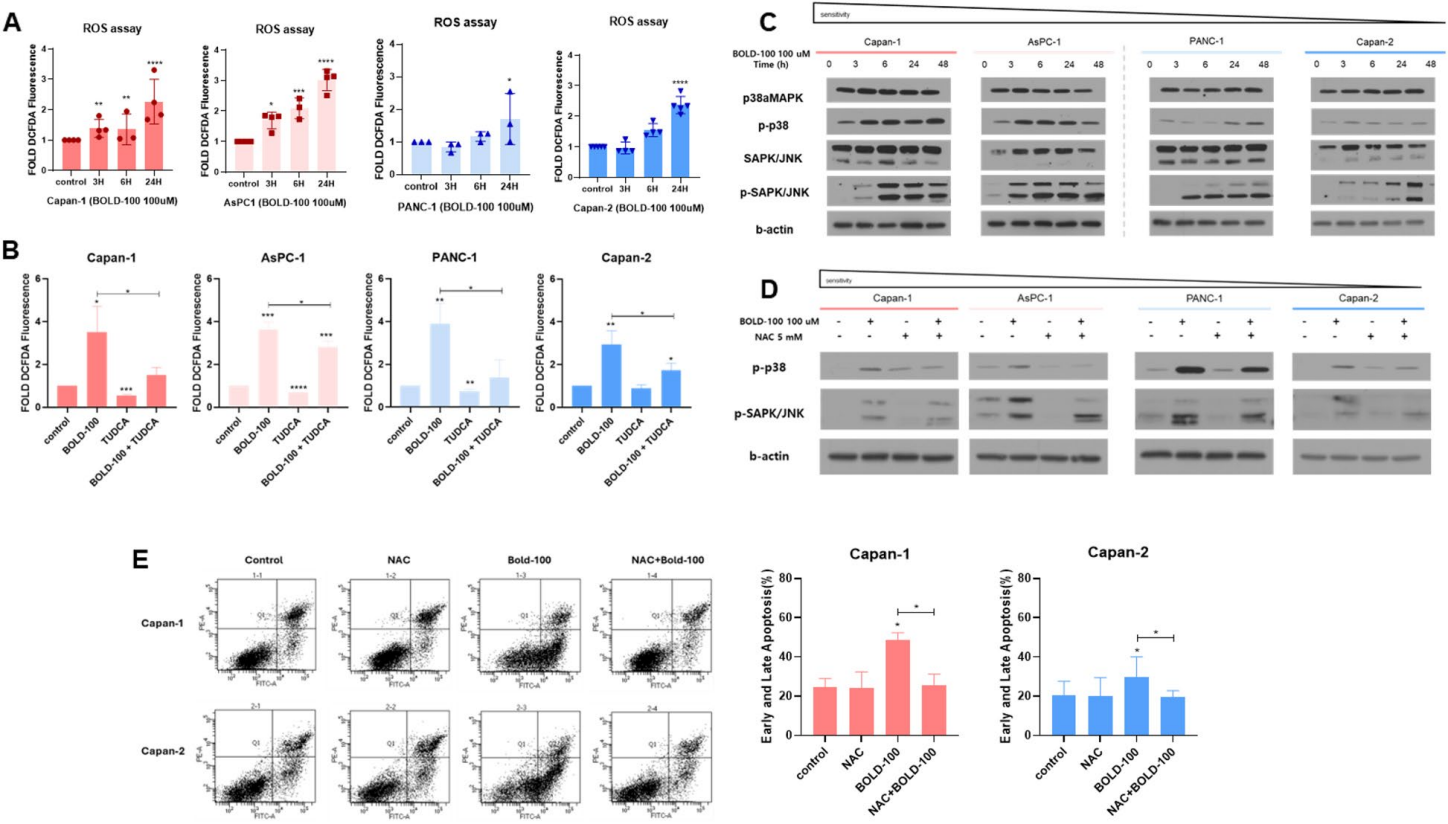
BOLD-100 induces apoptosis via ER stress-dependent ROS. **A** ROS levels of PDAC cells incubated with 100 µM BOLD-100. **B** ROS levels of PDAC cells incubated with BOLD-100 (100 µM) 24 h or TUDCA (100 µM) for 24 h or BOLD-100 (100 µM) plus TUDCA (100 µM) for 24 h pretreated cells. **C** Cells were exposed to time-dependent BOLD-100 (100 µM) treatment. Whole-cell extracts were used for western blotting. β-Actin was used as the loading control. **D** Cells incubated with NAC (5 mM) or BOLD-100 (100 µM) or NAC (5 mM) for 2 h, followed by 100 µM BOLD-100. Whole-cell extracts were used for western blotting. β-Actin was used as the loading control. **E** Flow cytometry was used for apoptosis analysis of BOLD-100 (100 µM) for 24 h or NAC (5 mM) for 3 h or or NAC (5 mM) plus BOLD-100 (100 µM)-treated cells. Values were calculated based on three independent experiments and presented as mean ± SD ∗, *p* < 0.05; ∗∗, *p* < 0.005; ***, *p* < 0.001

Previous studies have demonstrated that ER stress and ROS amplify each other in a feed-forward loop, disrupting cell function and activating pro-apoptotic pathways [20, 32, 33]. We observed an increase in ROS level with BOLD-100 treatment, as confirmed by enhanced DCF-DA probe levels, and reduction in the BOLD-100-induced ROS levels following TUDCA treatment (Fig. 3A, B). Additionally, BOLD-100 activated the p38/JNK pathway, which is a critical mediator of ROS-induced cell death, in response to oxidative stress (Fig. 3C) [34, 35]. To clarify the role of ROS in BOLD-100-induced apoptosis, we treated PDAC cells with the antioxidant N-acetylcysteine (NAC), which suppressed the phosphorylation of p38 and JNK and reduced apoptosis rates following BOLD-100 (Fig. 3D, E). These results indicate that ROS generated during ER stress plays a crucial role in BOLD-100-induced apoptosis through the JNK/p38 pathway.

### Increased ROS by BOLD-100 triggers the DNA damage response

**Fig. 4:**
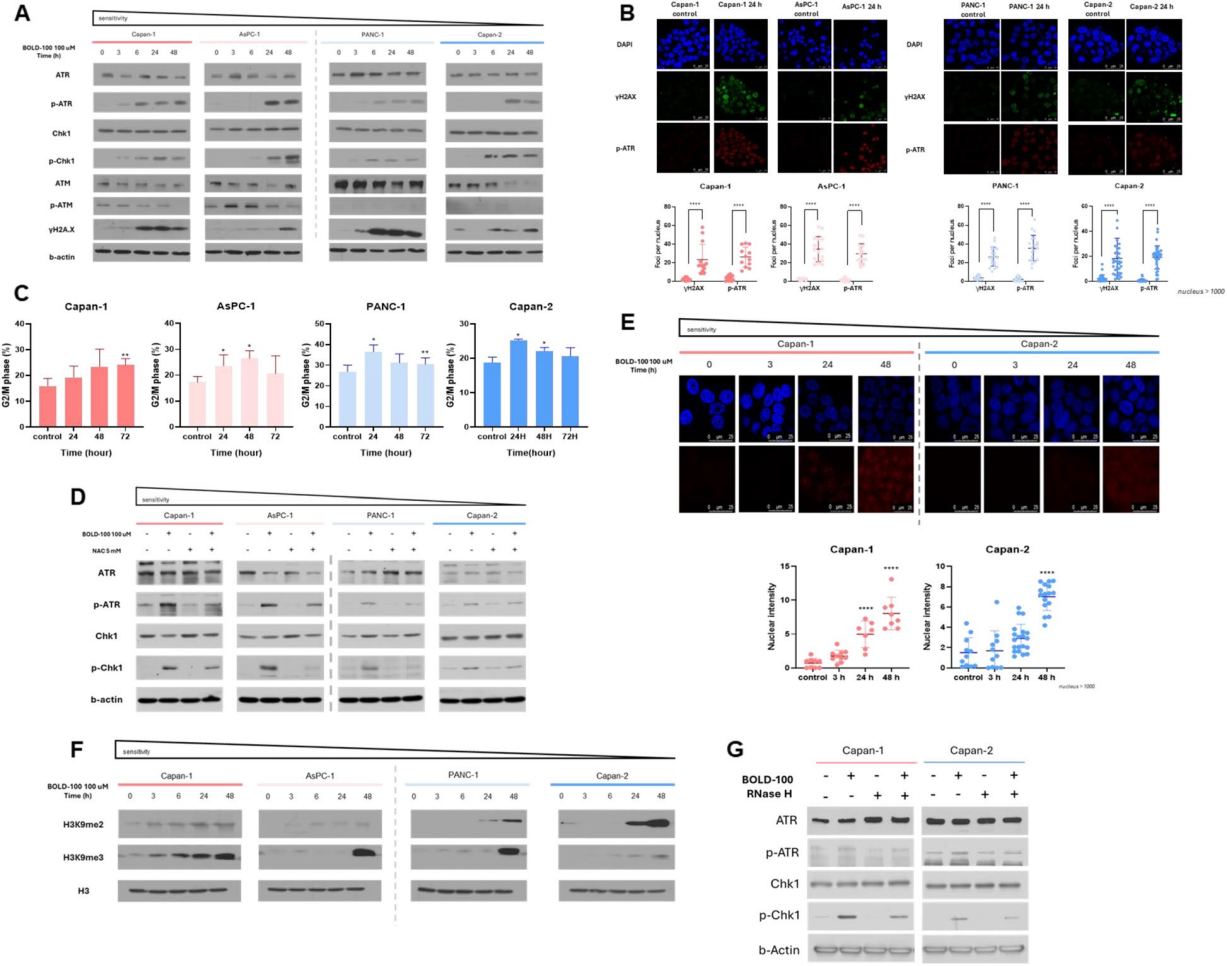
BOLD-100 induces ROS-dependent ATR/Chk1 activation in PDAC cells. **A** Protein expression levels of ATR, p-ATR, Chk1, p-Chk1 and γ-H2 AX. Cells were treated with BOLD-100 (100 µM) in a time-dependent manner (0, 3, 6, 24, and 48 h). **B** Immunofluorescence assays were carried out as described. After the indicated cells were exposed to BOLD-100 (100 µM) in a time-dependent manner, cells were stained for γ-H2 AX (green) and p-ATR (red) and counterstained with DAPI (blue). Representative images from three experiments are shown and the scale bar is 10 µm. The percentage of γ-H2 AX- or p-ATR-positive cells was determined by examining n >1000 nuclei per sample. Data from three independent experiments are depicted as mean ± SEM *, *p* < 0.05. **C** Cell cycle analysis together with flow cytometry was performed after 100 µM BOLD-100 treatment in the indicated cells. Data of quantitative measurement of cell cycle distribution of triplicate repeats are depicted as mean ± SEM. Data are presented from three independent experiments. **D** Cells incubated with BOLD-100 (100 µM) for 24 h or NAC (5 mM) for 2 h or NAC followed by 100 µM BOLD-100. Whole-cell extracts were used for western blotting. β-Actin was used as the loading control. **E** Cells were treated with BOLD-100 for the indicated periods. After fixation, cells were permeabilized with 0.5% Triton X-100 and then, stained for S9.6 (red) and counterstained with DAPI (blue). Representative images are presented, and the scale bar is 10 µm. The percentage of S9.6-positive cells was determined by examining n >1000 nuclei per sample. Values were calculated based on three independent experiments and presented as mean ± SD ∗, *p* < 0.05; ∗∗, *p* < 0.005; ***, *p* < 0.001. **F** Protein expression levels of H3 K9 me2 and H3 K9 me3. Cells were treated with BOLD-100 (100 µM) in a time-dependent manner (0, 3, 6, 24, and 48 h). **G** Protein expression levels of p-ATR and p-Chk1. Cells were treated with BOLD-100 (100 µM) for 24 h and RNase H (0.1 U). GAPDH was used as the loading control

ROS can cause DNA damage by generating oxidative lesions, such as 8-oxoguanine, which lead to base modifications, single-strand breaks (SSBs), and double-strand breaks (DSBs). DNA sensors ATM and ATR are both involved in detecting these lesions, initiating DDR pathways responsible for subsequent DNA repair and promoting cancer cell survival [23, 36]. We found that BOLD-100 treatment activated the ATR/Chk1 axis, with only a transient and limited effect on ATM activation, along with a time-dependent increase in γ-H2 AX, a marker of DNA double-strand breaks (DSBs) (Fig. 4A). Immunofluorescence analysis confirmed increased p-ATR and γ-H2 AX foci, further indicating significant DNA damage and selective activation of the ATR pathway (Fig. 4B). These findings suggest that ATR plays a predominant role in ROS-mediated DDR activation in this context. ATR activation leads to Chk1 phosphorylation, cdc25c inhibition, and subsequent G2/M cell cycle arrest [37]. Consistent with this, BOLD-100 treatment resulted in a time-dependent accumulation of cells in the G2/M phase (Fig. 4C). To explore the roles of ROS in this process, we treated cells with NAC, which suppressed the BOLD-100-induced upregulation of p-ATR and p-Chk1 (Fig. 4D). While ATR is known to regulate base excision repair (BER) pathways, we found no significant changes in the expression of BER-related proteins following BOLD-100 treatment (Fig S2). This finding suggests that BOLD-100 may not influence BER in this context. Instead, ATR activation appeared to be linked to R-loops, which form during cellular stress when stalled replication forks allow RNA to hybridize with DNA [23, 38]. These R-loops exacerbate replication stress and activate the ATR/Chk1 pathway as the cell attempts to stabilize replication forks and mitigate DNA damage. Using the S9.6 antibody to detect RNA/DNA hybrids, we found that BOLD-100-induced ROS increased S9.6 nuclear intensity, indicating R-loop accumulation (Fig. 4E, Fig S3). Furthermore, BOLD-100 treatment elevated levels of H3 K9 me2 and H3 K9 me3 (Fig. 4F), histone modifications associated with transcriptional repression and heterochromatin formation, often observed as a consequence of R-loop formation. Supporting the role of R-loops in ATR/Chk1 activation, RNase H treatment, which resolves R-loops by degrading RNA in RNA/DNA hybrids, reversed BOLD-100-induced phosphorylation of ATR and Chk1 (Fig. 4G). These findings collectively demonstrate that R-loop accumulation plays a critical role in driving ATR/Chk1 activation under BOLD-100 treatment.

### Synergistic anti-tumor effects of BOLD-100 and ATR inhibitor in PDAC cells

**Fig. 5:**
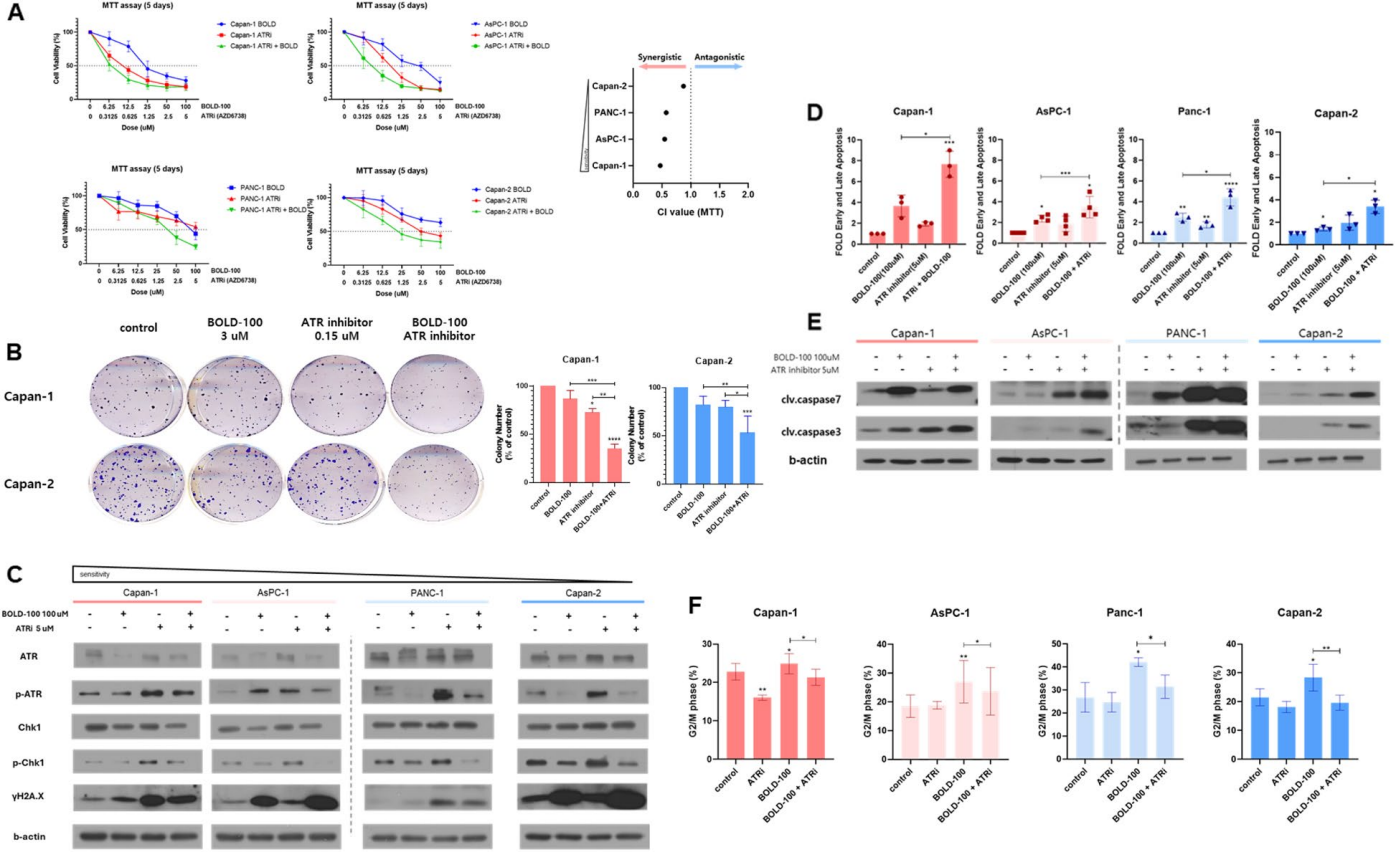
Effects of combination treatment with GRP78 and ATR inhibitors. **A** Each cell line was exposed to various concentrations of BOLD-100 (0, 12.5, 25, 50, and 100 µM), AZD6738 (0, 0.625, 1.25, 2.5, and 5 µM), and increasing concentrations of BOLD-100 (0, 12.5, 25, 50, and 100 µM) plus AZD6738 (0, 0.625, 1.25, 2.5, and 5 µM). After 120 h of incubation following treatment, an MTT assay was performed. Data on the percentage of viable cells from at least three independent experiments are shown in each graph. **B** Clonogenic assays were performed after cells were treated with the indicated doses of BOLD-100 (50 µM), AZD6738 (2.5 µM), or a combination of BOLD-100 (50 µM), and AZD6738 (2.5 µM) for 9 days. Quantification of clonogenic assays was performed in more than three independent experiments. Scale bars are presented as mean ± SEM *, *p* < 0.05; **, *p* < 0.005; ***, *p* < 0.001. **C** Protein expression levels of ATR, p-ATR, Chk1, p-Chk1, and γ-H2 AX. Cells were treated with BOLD-100 (100 µM), AZD6738 (5 µM), or BOLD-100 (100 µM) plus AZD6738 (5 µM) for 24 h. **D** FACS-based evaluation of apoptosis rates in PDAC cells. Cells were treated with BOLD-100 (100 µM) 24 h, AZD6738 (5 µM) 24 h, or BOLD-100 (100 µM) plus AZD6738 (5 µM) 24 h. Values were calculated based on three independent experiments and presented as mean ± SD ∗, *p* < 0.05; ∗∗, *p* < 0.005; ***, *p* < 0.001. **E** Whole-cell extracts from cells treated with BOLD-100 (100 µM) 24 h, AZD6738 (5 µM) 24 h, or BOLD-100 (100 µM) plus AZD6738 (5 µM) 24 h were used for immunoblotting. GAPDH was used as the loading control. **F** Cell cycle analysis together with flow cytometry was performed after 100 µM BOLD-100 treatment and BOLD-100 (100 µM) plus AZD6738 (5 µM) 24 h in the indicated cells. Data of quantitative measurement of cell cycle distribution of triplicate repeats are depicted as mean ± SEM. Data are presented from three independent experiments

The above results provide evidence supporting the evaluation of the combination treatment of BOLD-100 with the ATR inhibitor (AZD6738). To assess the synergistic effects of these combined regimens, we conducted 120-h MTT assays. Significant synergistic effects were observed in PDAC cells with the combined treatment of BOLD-100 and AZD6738 (Fig. 5A). his was further supported by the substantial reduction in the colony formation by the combination treatment in Capan-1 and Capan-2 cells (Fig. 5B). Co-treatment with AZD6738 significantly attenuated the BOLD-100-induced upregulation of p-ATR and p-Chk1. Additionally, an increase in γ-H2 AX accumulation was observed across all cell lines under co-treatment (Fig. 5C). The combined treatment enhanced apoptosis, with elevated levels of cleaved caspase 7 and cleaved caspase 3 compared to the others (Fig. 5D, E). Furthermore, the observed accumulation of cells in the G2/M phase, induced by ATR activation, was significantly reduced with co-treatment of AZD6738 (Fig. 5F), suggesting disruption of cell cycle arrest and promotion of cell death. These findings indicate that the combination of BOLD-100 and AZD6738 synergistically enhances anti-tumor effects in vitro.

### Dual inhibition of GRP78 and ATR enhances anti-tumor activity in a xenograft model

**Fig. 6:**
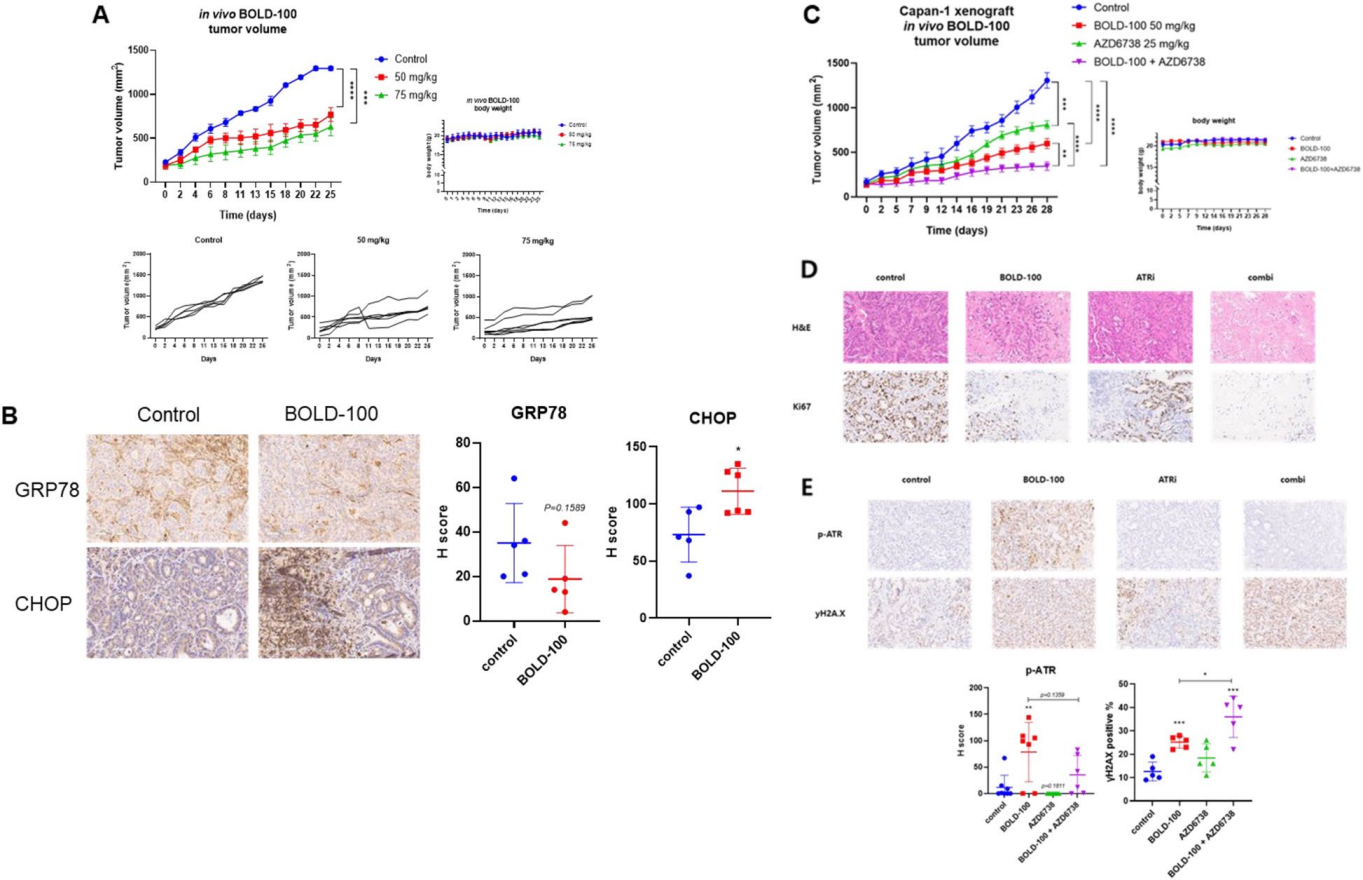
Anti-tumor effects of GRP78 and ATR co-inhibition in the xenograft model. **A** Growth curves of subcutaneously transplanted Capan-1 tumors upon treatment with vehicle (citrate buffered saline)/BOLD-100 (50 mg/kg and 75 mg/kg). Tumor volumes are presented as mean ± SEM (n = 5 for control, n = 6 for 50 mg/kg, and n = 7 for 75 mg/kg). One mouse in the 50 mg/kg group exhibited a transient volume spike followed by a rapid decrease; however, the tumor was confirmed to be intact upon dissection and was retained in the analysis. ns: not significant; *, *p <* 0.05; **, *p <* 0.005. Individual tumor growth curves and body weight data are also shown for each treatment group. **B** Representative images of immunohistochemical analysis of transplanted Capan-1 tumors collected from the animals upon the end of the 4-week treatment schedule. Tissue slides were stained with GRP78 and CHOP. *p < 0.05, **p < 0.01, ***p < 0.001. (C) Tumor growth curve and mouse body weights in Capan-1 xenografted mice treated with vehicle, BOLD-100 (50 mg/kg), AZD6738 (25 mg/kg), or both BOLD-100 and AZD6738 for up to 4 weeks. (D) Representative images of immunohistochemical analysis of transplanted Capan-1 tumors collected from the animals upon the end of the 4-week treatment schedule. Tissue slides were stained with H&E and Ki67. (E) Immunohistochemical analysis of p-ATR and gamma H2 A.X, staining in Capan-1 xenograft excised tumors. *p < 0.05, **p < 0.01, ***p < 0.001

We established a xenograft model using the Capan-1 cell line to confirm our finding in vivo. BOLD-100 monotherapy suppressed tumor growth in Capan-1 xenograft mice at 50 mg/kg and 75 mg/kg, while the body weight remained unchanged between the BOLD-100 and vehicle groups (Fig. 6A). BOLD-100 treatment induced ER stress and exhibited antitumor effects, as indicated by increased CHOP and a trend toward GRP78 downregulation in vivo, although the latter did not reach statistical significance (*p* = 0.1589) (Fig. 6B). Based on the in vitro observation, we investigated the in vivo anti-tumor effect of the combination strategy of BOLD-100 and AZD6738 using Capan-1 xenograft model. Notably, both BOLD-100 and AZD6738 monotherapy suppressed tumor growth, while the combination regimen significantly inhibited tumor growth (Fig. 6C). During the experiment, we did not observe body weight loss in any mouse, indicating that BOLD-100 did not cause significant adverse effects. Co-treatment of BOLD-100 and AZD6738 reduced tumor proliferation, as evidenced by a greater decrease in Ki67-positive cells compared to other groups (Fig. 6D). In addition, consistent with in vitro data, the BOLD-100-induced p-ATR induction was reversed by the combination regimen, while γ-H2 AX expression was upregulated (Fig. 6E). In conclusion, these IHC results indicate that the combination of BOLD-100 and AZD6738 enhances anti-tumor effects compared to monotherapy, suggesting a potential combination benefit in vivo.

## Discussion

This study demonstrates the therapeutic potential of targeting GRP78 using BOLD-100 in PDAC. By reducing GRP78 levels, BOLD-100 disrupts protein homeostasis, leading to persistent ER stress and UPR activation, culminating in CHOP-dependent apoptosis. The critical role of CHOP in this process was confirmed by siRNA-mediated CHOP knockdown, which reversed BOLD-100-induced cell death. These findings elucidate the mechanisms underlying BOLD-100-induced cancer cell death and underscore the pivotal role of CHOP in the ER stress response triggered by BOLD-100 in PDAC cells. Additionally, BOLD-100 elevates ROS levels, amplifying apoptotic pathways through the JNK and p38 axis. ROS-mediated DNA damage further activates the ATR and Chk1 pathway, linking ROS to DDR activation.

Unlike prior studies that inferred ER stress based on stress marker expression, our findings provide functional evidence that BOLD-100 directly impairs protein folding capacity, leading to the accumulation of misfolded proteins [39, 40]. This mechanistic insight strengthens the causal link between GRP78 suppression and sustained activation of the UPR, rather than relying solely on downstream transcriptional changes. As proteotoxic stress escalates, it may initiate secondary layers of cellular stress, including replication stress and genome instability. In this context, BOLD-100 was found to induce R-loop accumulation, suggesting that replication fork stalling may be involved in this process [41–43]. Transcription-replication conflicts driven by ROS-induced stress likely exacerbate replication stress and genomic instability. These R-loops not only trigger the ATR/Chk1 pathway but also lead to epigenetic reprogramming, marked by highly compacted heterochromatin with increased histone methylation (H3 K9 me2 and H3 K9 me3) [44–47]. These epigenetic modifications halt transcription machinery to stabilize the genome under stress but may also create vulnerabilities that can be therapeutically exploited. Targeting histone methyltransferases involved in these R-loop-mediated epigenetic changes, such as G9a/GLP and SETDB1, could enhance the anti-tumor effects of BOLD-100 [48–50]. Moreover, the putative interplay between R-loop-induced epigenetic changes and DDR activation highlights a complex crosstalk, underscoring the potential for novel therapeutic approaches that integrate epigenetic and DDR-targeting strategies.

In combination with the ATR inhibitor AZD6738, BOLD-100 exhibited synergistic effects in vitro and enhanced anti-tumor activity in vivo, significantly suppressing tumor growth in a Capan-1 xenograft model. While a recent study demonstrated BOLD-100’s modulation of the ATR signaling axis in colorectal cancer cells, our work represents the first in vivo validation of a BOLD-100 and ATR inhibitor combination regimen. Notably, this is also the first study to explore this combination in pancreatic cancer, offering translational insight into a therapeutic strategy with relevance for hard-to-treat tumor types [51]. These findings highlight the dual role of BOLD-100 in modulating ER stress and DDR pathways, providing a strong rationale for its use in combination therapies. While BOLD-100 showed comparable anti-tumor effects across all tested PDAC cell lines with similar mechanisms of action, GRP78 expression did not correlate with sensitivity to BOLD-100 (data not shown). This suggests that GRP78 levels alone may not serve as a predictive biomarker. GRP78 dynamics are highly unstable, with a fast turnover rate tightly regulated by diverse post-translational modifications in response to stress, complicating its use as a biomarker. Future efforts should focus on identifying robust predictive biomarkers. Large cohort studies could clarify subtype-specific responses to BOLD-100, utilizing transcriptional classifications, such as basal-like versus classical subtypes, or genotypic differences like KRAS mutation status. Furthermore, although our data support GRP78 as a key mediator of BOLD-100-induced ER stress and apoptosis, the target specificity of BOLD-100 remains to be fully elucidated. Direct binding assays, rescue experiments, or target engagement studies were not performed in this study and will be essential in future research to confirm GRP78 as a primary molecular target. The mechanism by which BOLD-100 downregulates GRP78 is also not fully understood. Proteomic evidence suggests that BOLD-100 may interact with GTF2I, a transcription factor known to activate GRP78 expression, and that disruption of this interaction could plausibly explain the observed GRP78 reduction and subsequent ER stress activation [52].

While our findings provide compelling evidence for the therapeutic potential of BOLD-100, several limitations warrant consideration. R-loop accumulation and ATR activation were demonstrated, but direct evidence of replication stress, such as changes in replication fork dynamics or comprehensive DDR protein profiling, remains unexplored. Future studies should investigate replication fork progression and stability at a mechanistic level to elucidate the impact of ROS-induced R-loops. Additionally, while we tested the Capan-1 xenograft model, a cell line representative of homologous recombination repair deficiency (HRD) due to BRCA2 deletion, other cell lines without an HRD background were not fully addressed in our in vivo model. This limitation is particularly relevant given that the only currently approved DDR-acting agent, a PARP inhibitor, is primarily effective in patients with germline BRCA mutations. Expanding the application of DDR inhibitors beyond HRD remains an important objective, and future studies should evaluate the efficacy of BOLD-100 and ATR inhibitors in models representative of diverse genomic contexts.

In conclusion, this study identifies GRP78 as a viable therapeutic target in PDAC and establishes BOLD-100 as an effective agent for inducing ER stress and apoptosis. Its synergistic interaction with ATR inhibitor in vitro and its enhanced anti-tumor efficacy offer a novel combination strategy for PDAC treatment. These findings provide a foundation for future research and clinical translation of BOLD-100 in combination therapies for the treatment of patients with PDAC.

## Supplementary Information

Supplementary Material 1

Supplementary Material 2

## Data Availability

No datasets were generated or analysed during the current study.
