# Supplementary material for: Therapeutic potential of BOLD-100, a GRP78 inhibitor, enhanced by ATR inhibition in pancreatic ductal adenocarcinoma
Source: Cell Commun Signal. 2025 Jun 13;23:281. doi: 10.1186/s12964-025-02242-8 (PMC12164152; doi:10.1186/s12964-025-02242-8)
Supplement: Supplementary file 1 — Supplementary Material 1 [file 12964_2025_2242_MOESM1_ESM.docx]

**Supplementary Information for**

**Therapeutic Potential of BOLD-100, a GRP78 Inhibitor, enhanced by ATR Inhibition in Pancreatic Ductal Adenocarcinoma**

Su In Lee^1,^ Ah-Rong Nam^1^, Kyoung-Seok Oh^1^, Jae-Min Kim^1,2^, Ju-Hee Bang^1^, Yoojin Jeong^1^, Sea Young Choo^1^, Hyo Jung Kim^1^, Jeesun Yoon^1,3^, Tae-Yong Kim^1,3^ and Do-Youn Oh^1,2,3^

^1^Cancer Research Institute, Seoul National University College of Medicine, Seoul 03080, Korea

^2^Integrated Major in Innovative Medical Science, Seoul National University Graduate School, Seoul 03080, Korea

^3^Department of Internal Medicine, Seoul National University Hospital, Seoul 03080, Korea

Correspondence:

Do-Youn Oh, MD, Ph.D.

Professor,

Department of Internal Medicine, Seoul National University Hospital, Seoul, Korea

Cancer Research Institute, Seoul National University College of Medicine, Seoul, Korea

101 Daehak-ro, Jongno-gu, Seoul 03080, Korea;

Tel: +82-2-2072-0701; Fax: +82-2-762-9662;

**Email:** [ohdoyoun@snu.ac.kr](mailto:ohdoyoun@snu.ac.kr)

**
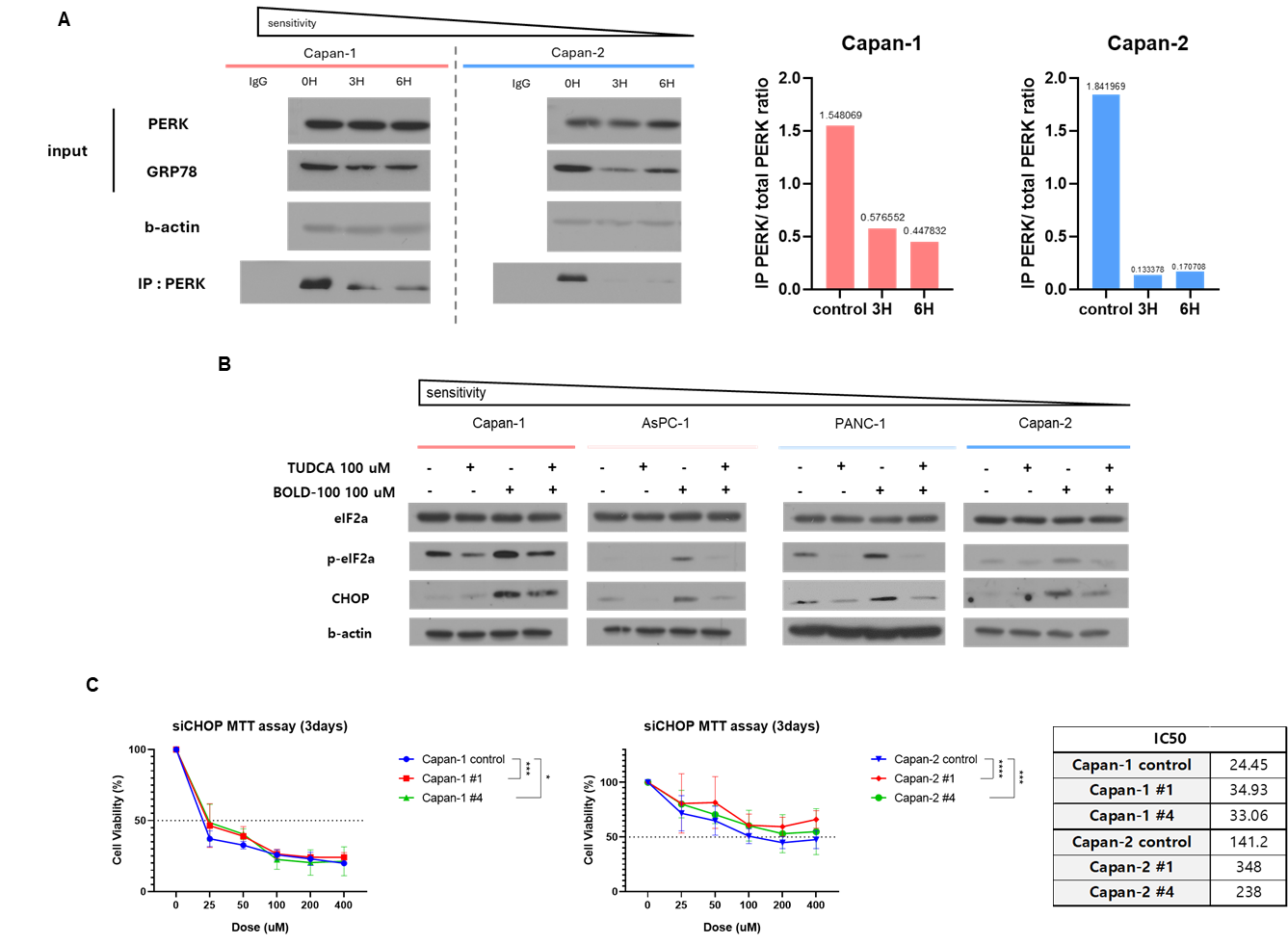
**

**Fig. S1. BOLD-100 induces apoptosis through ER stress mechanisms.** (A) Cells were treated with 100 µM BOLD-100 for 6 h. Immunoprecipitation was performed using an anti-PERK antibody, followed by western blot analysis to assess the interaction between GRP78 and PERK. GRP78 input was included as a positive control (left panel). The ratio of immunoprecipitated PERK to total PERK was quantified from whole cell lysates using densitometric analysis in ImageJ (right panel). (B) Protein expression levels of PERK, eIF2α, p- eIF2α, and CHOP. The cells were incubated with TUDCA (100 µM) for 24 h or BOLD-100 (100 µM) for 24 h or TUDCA (100 µM) plus BOLD-100 (100 µM)-treated cells. (C) Each cell line was exposed to various concentrations of BOLD-100 (0, 25, 50, 100, 200 and 400 µM). After 72 h of incubation following treatment, an MTT assay was performed. Data on the percentage of viable cells from at least three independent experiments are shown in each graph.


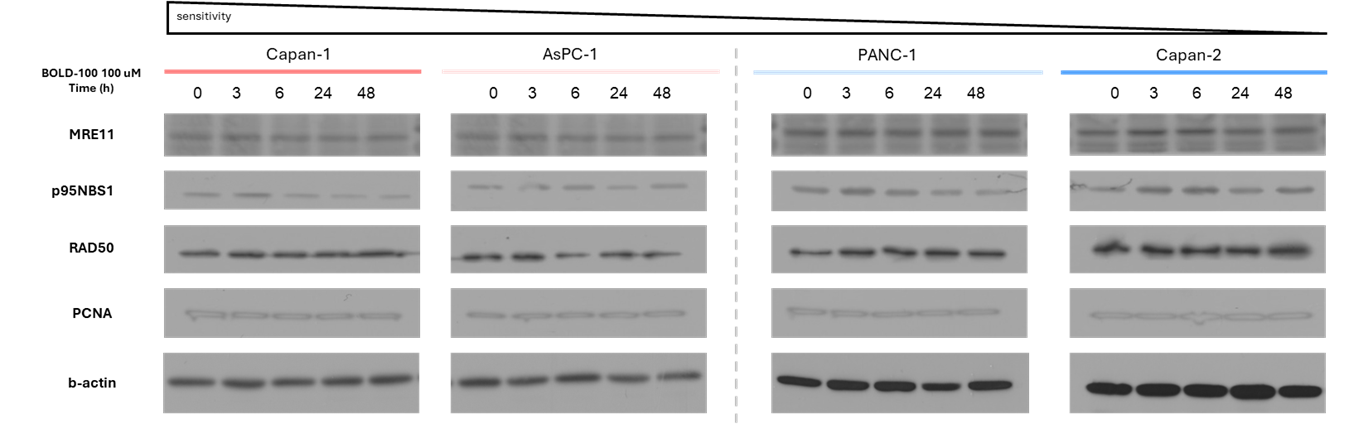


**Fig. S2. BOLD-100 does not impact BER pathways.** Protein expression levels of MRE11, p95NBS1, RAD50, and PCNA. Cells were treated with BOLD-100 (100 µM) in a time-dependent manner (0, 3, 6, 24, and 48 h). GAPDH was used as the loading control.


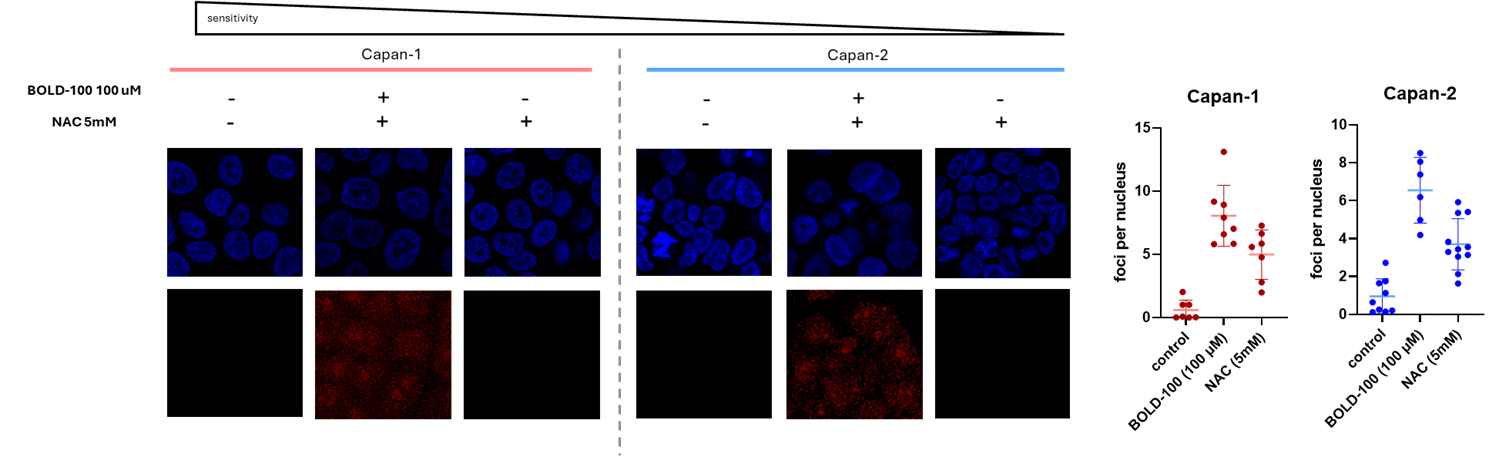


**Fig. S3. BOLD-100 induces R-loop formation through ROS.** Cells were treated with BOLD-100 (100 µM) for 48 h and NAC (5mM) for 3 h. After fixation, cells were permeabilized with 0.5% Triton X-100 and then, stained for S9.6 (red) and counterstained with DAPI (blue). Representative images are presented, and the scale bar is 10 µm. Result Is derived from one experiment.
