# Supplementary material for: Therapeutic potential of BOLD-100, a GRP78 inhibitor, enhanced by ATR inhibition in pancreatic ductal adenocarcinoma
Source: Cell Commun Signal. 2025 Jun 13;23:281. doi: 10.1186/s12964-025-02242-8 (PMC12164152; doi:10.1186/s12964-025-02242-8)

Figure 1D

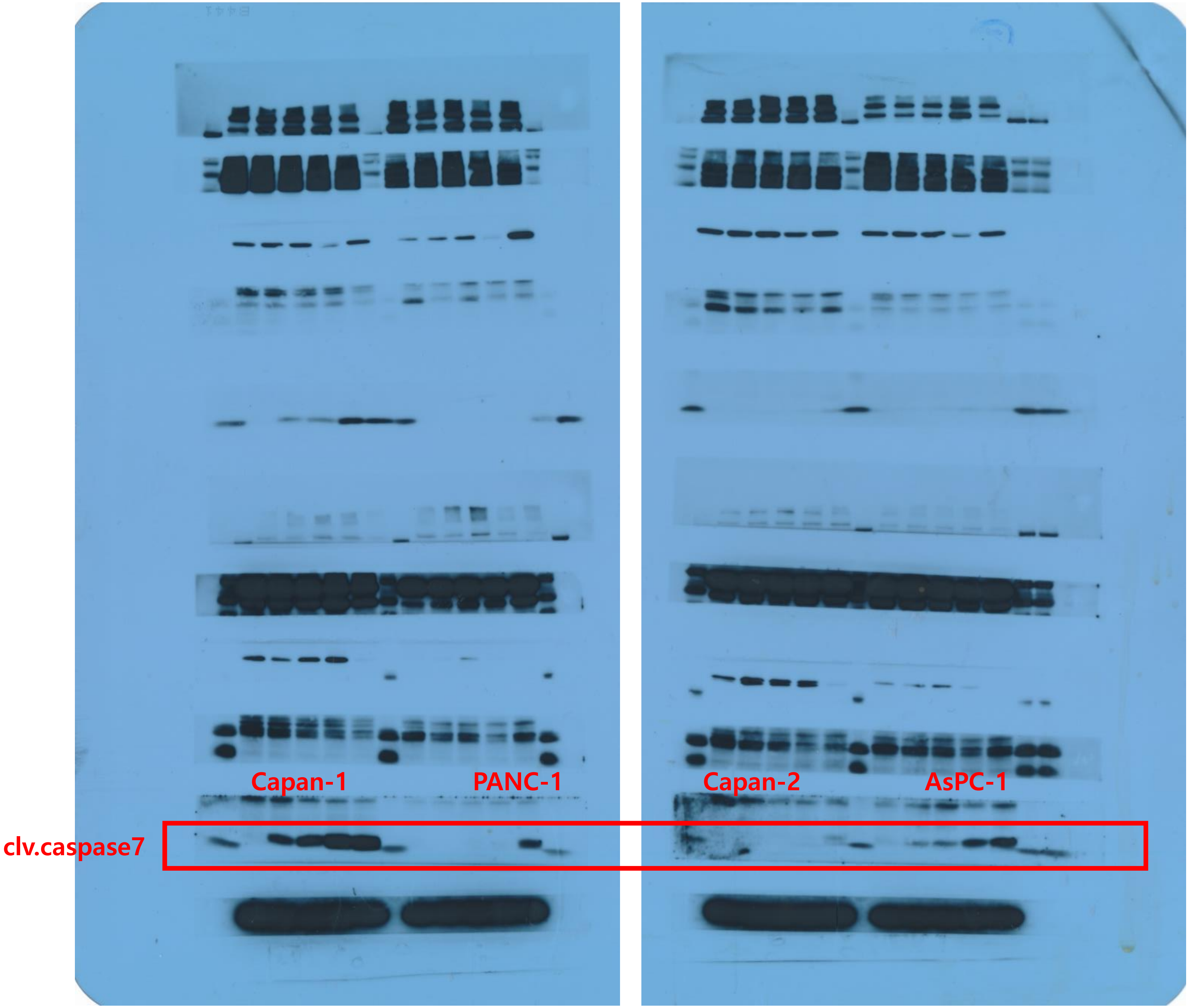

Figure 1D

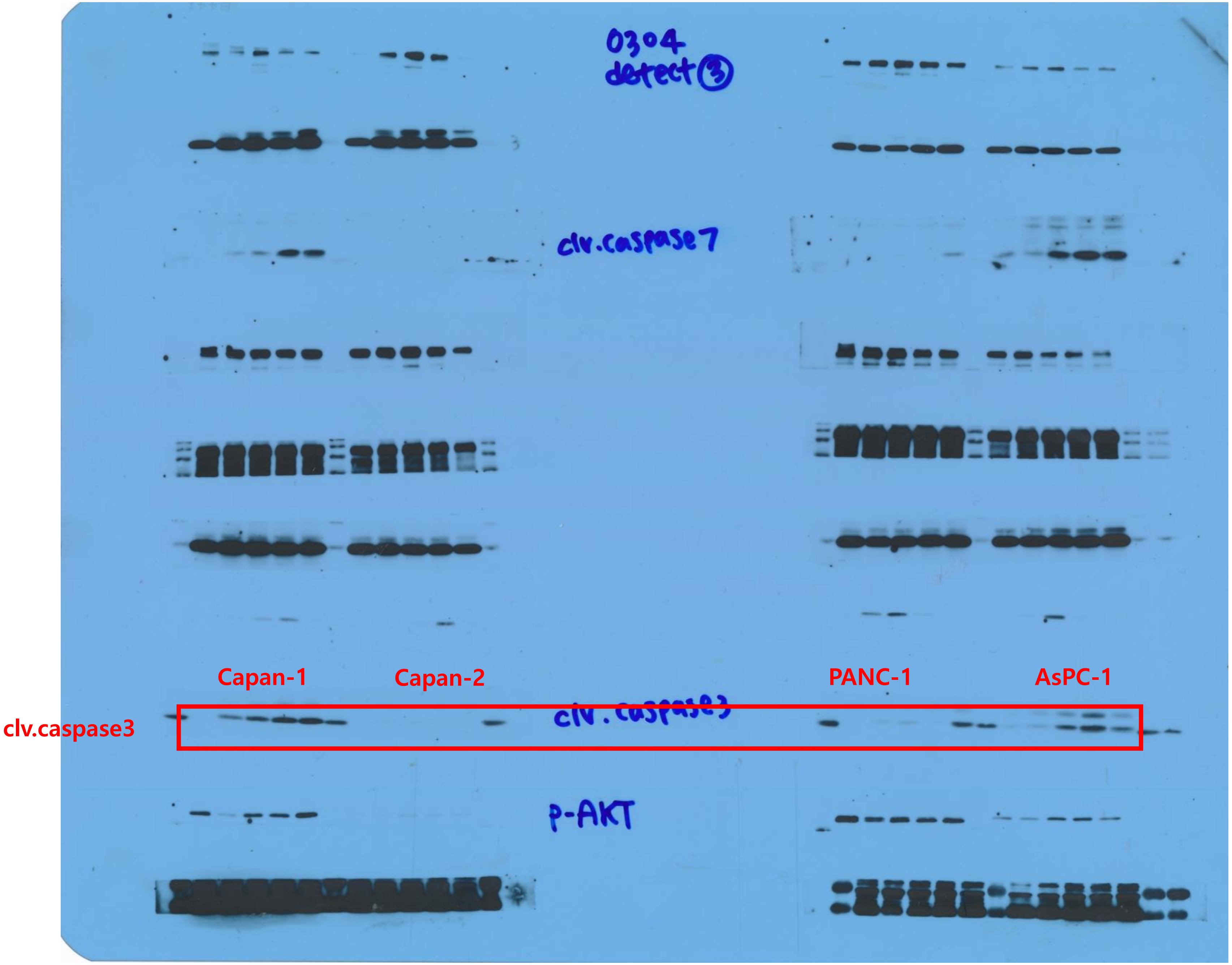

0304
detect③
clv.caspase7
Capan-1
Capan-2
PANC-1
AsPC-1
clv.caspase3
clv.caspase3
p-AKT

Figure 1D

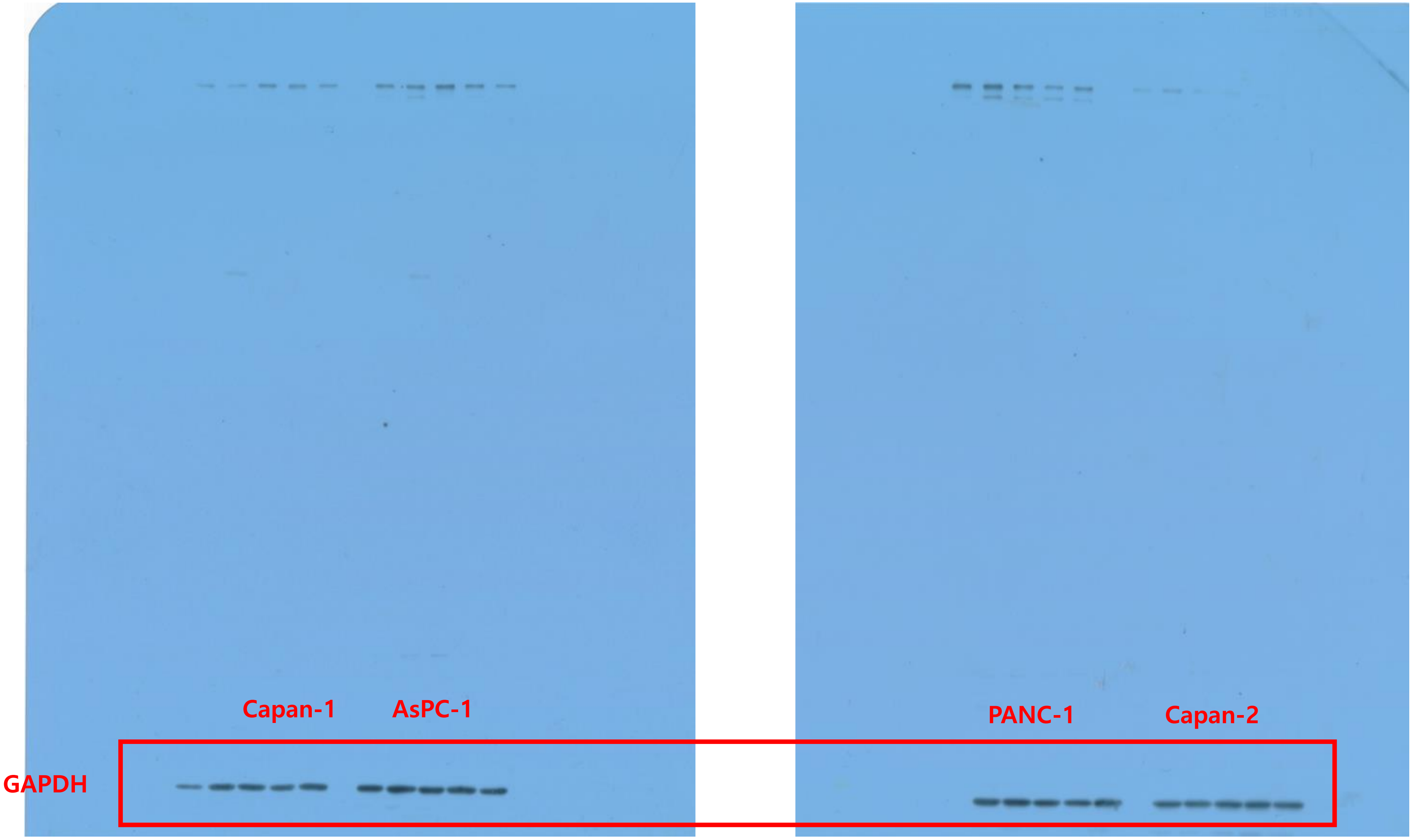

Figure 2B

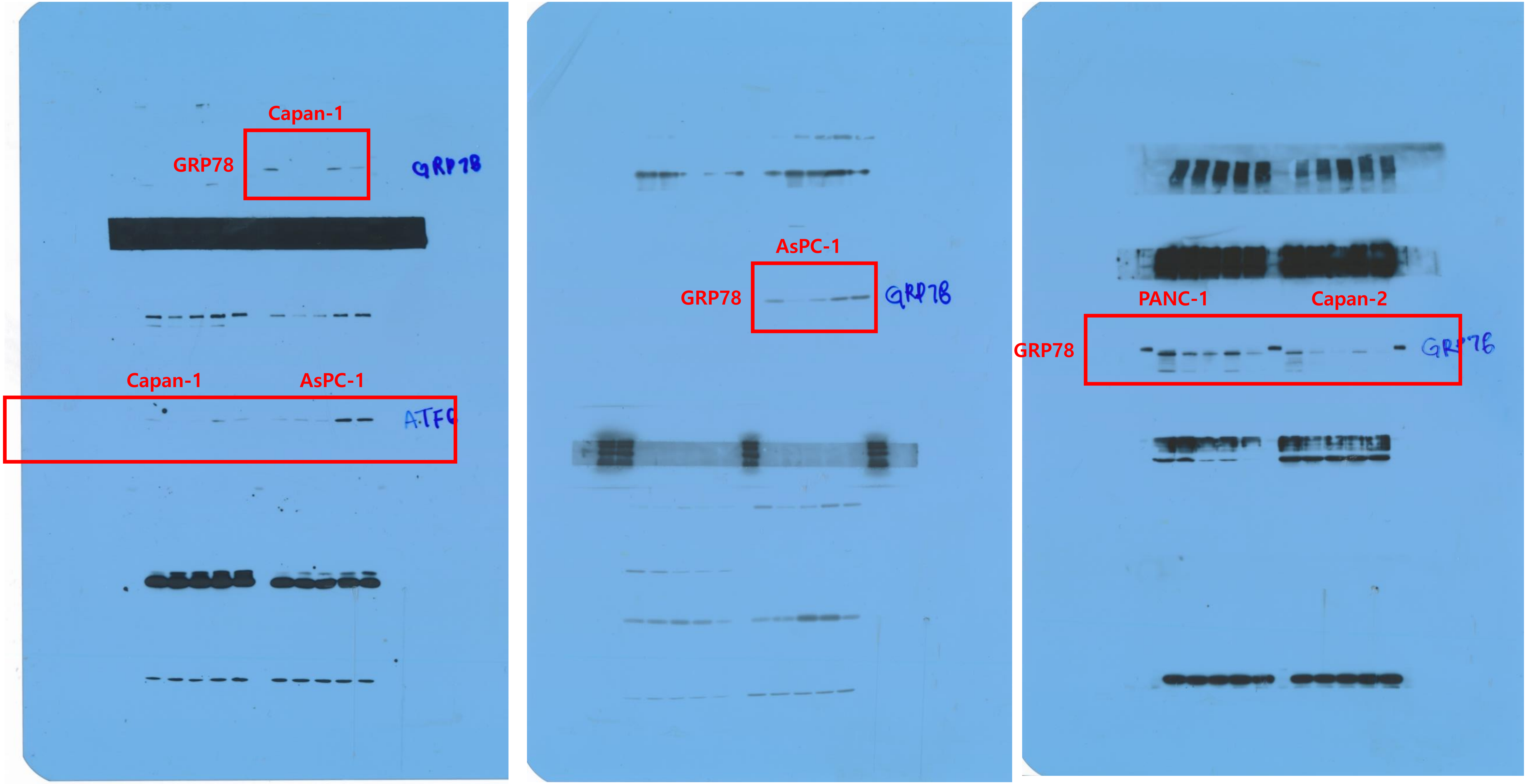

Capan-1
GRP78
GRP78
Capan-1
AsPC-1
ATF6
AsPC-1
GRP78
GRP78
PANC-1
Capan-2
GRP78
GRP78

Figure 2B

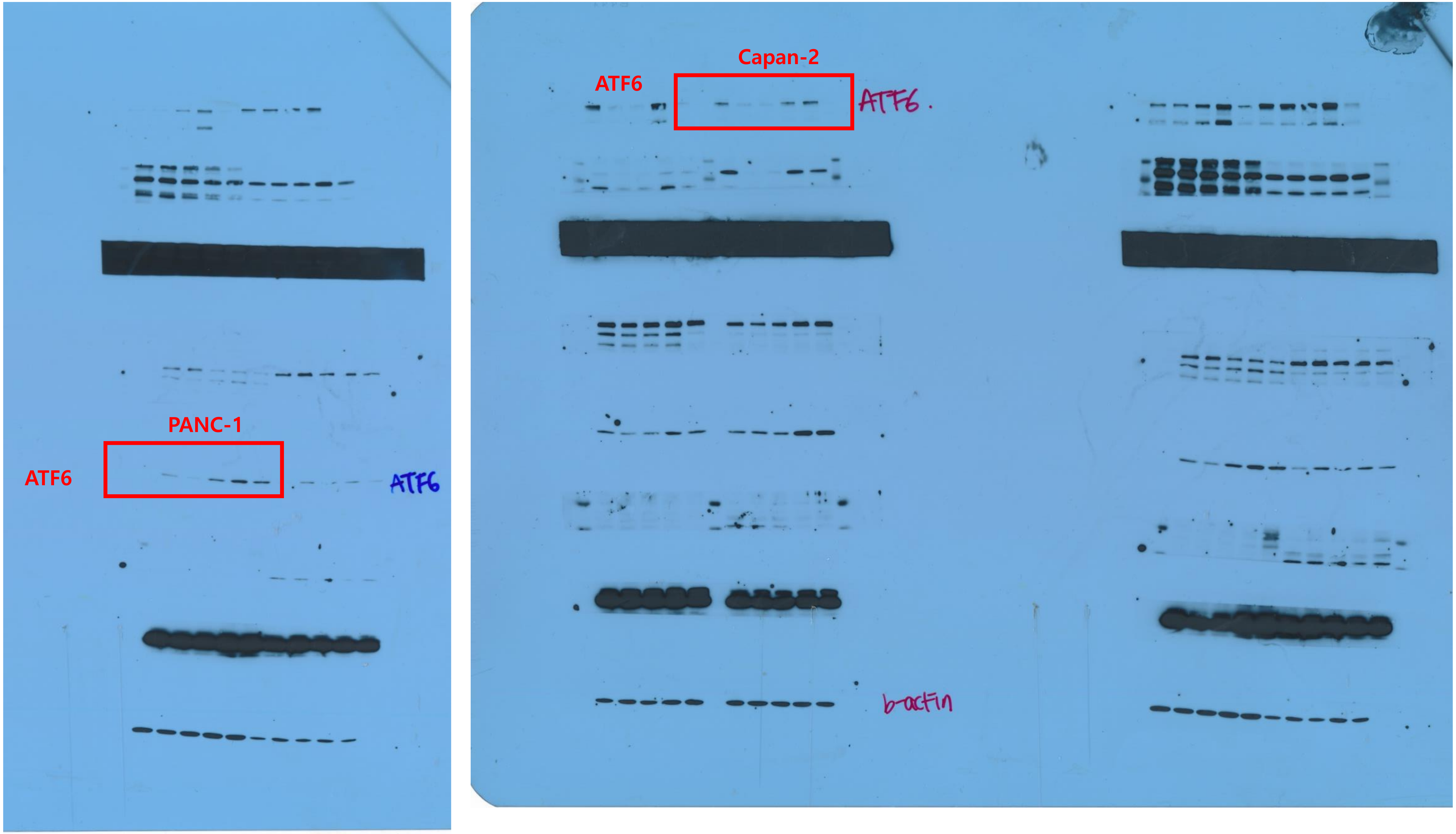

Figure 2B

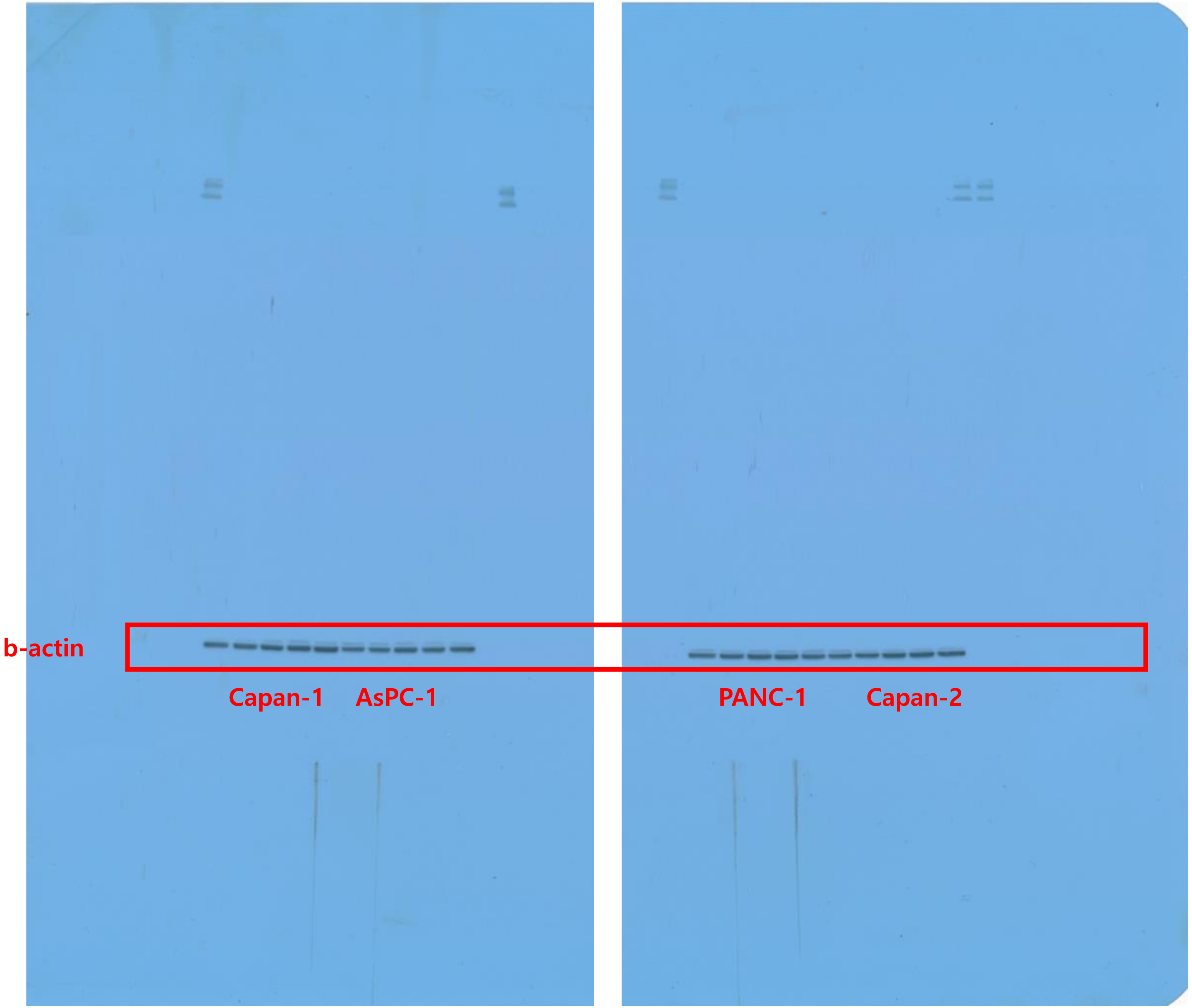

Figure 2D

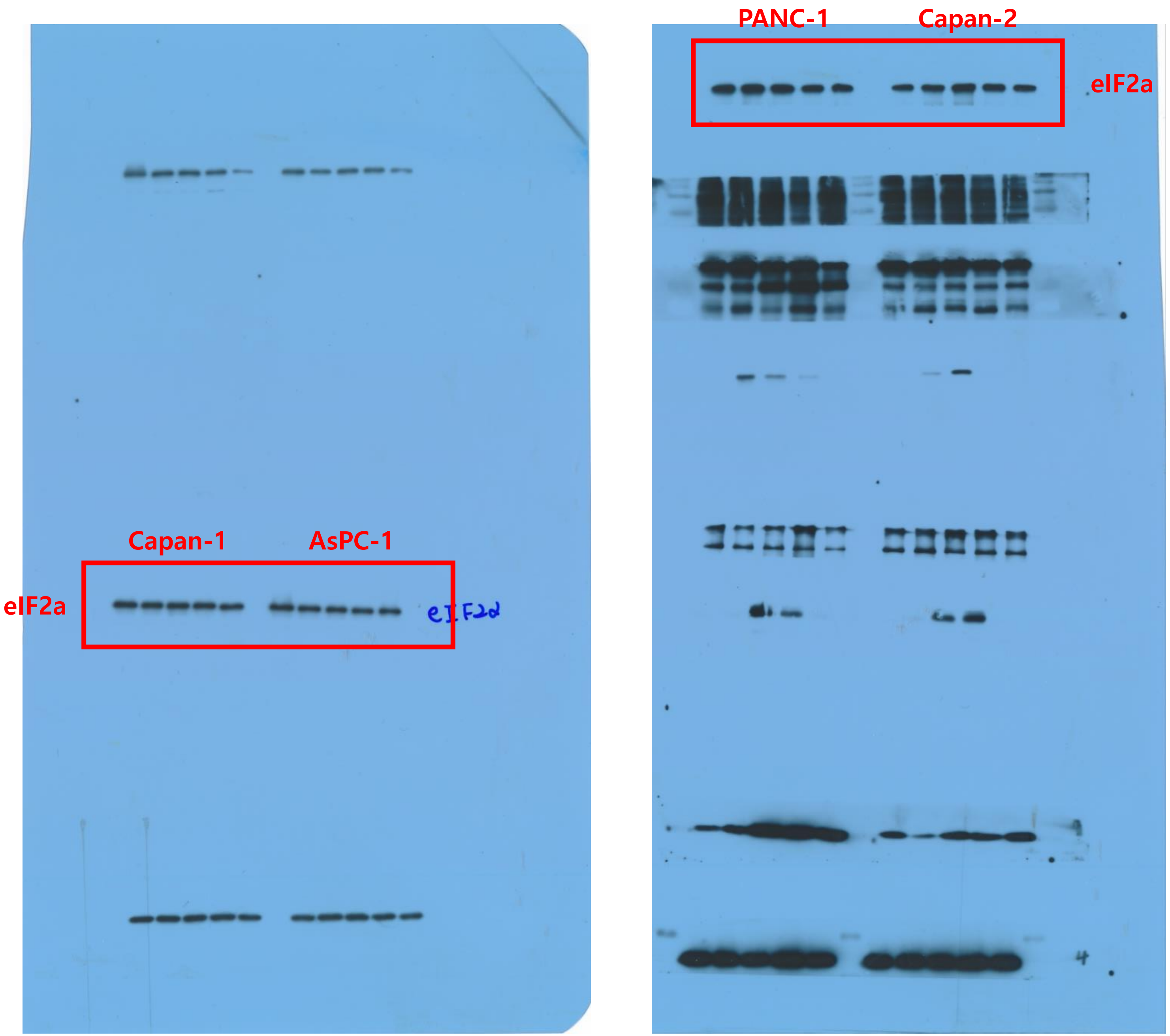

Figure 2D

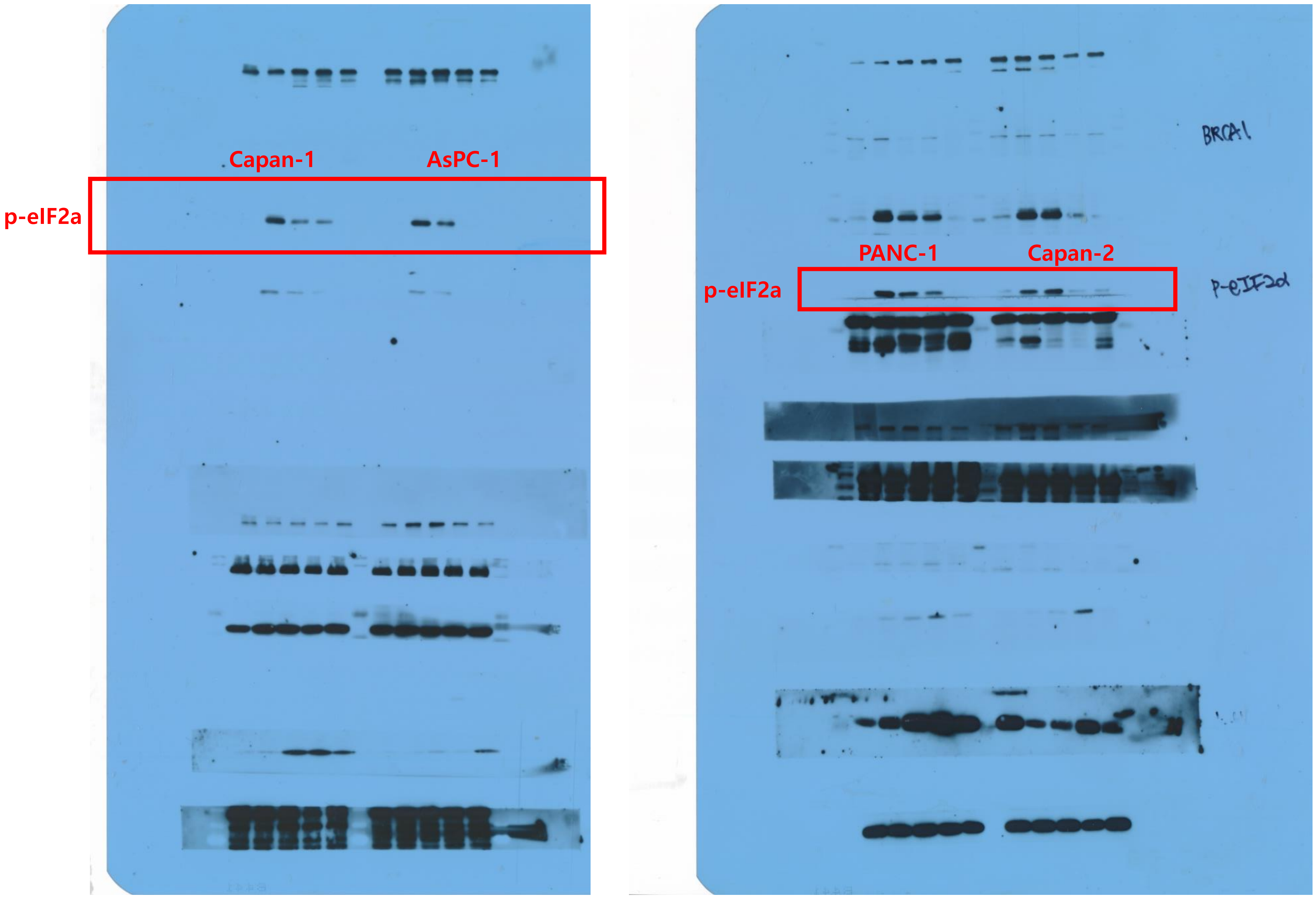

Figure 2D

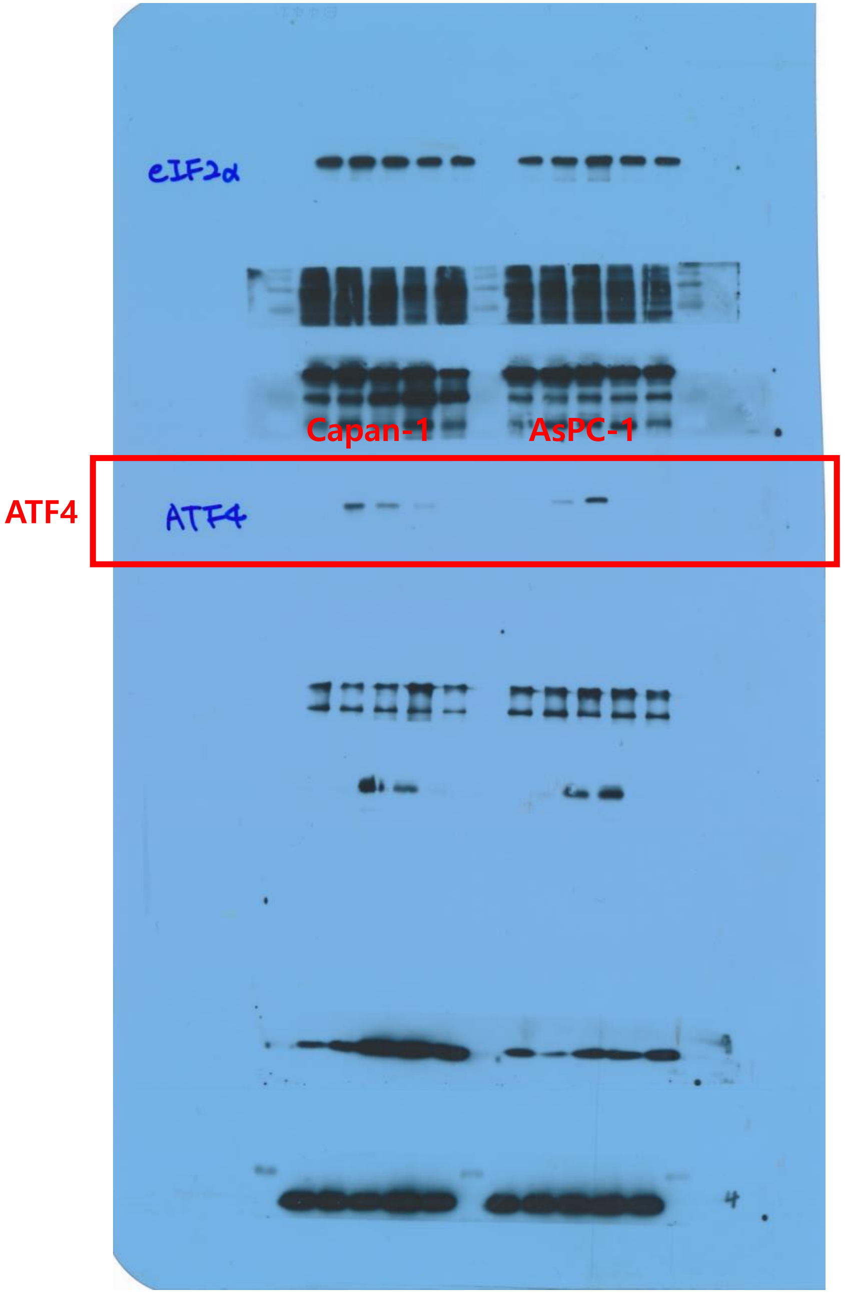

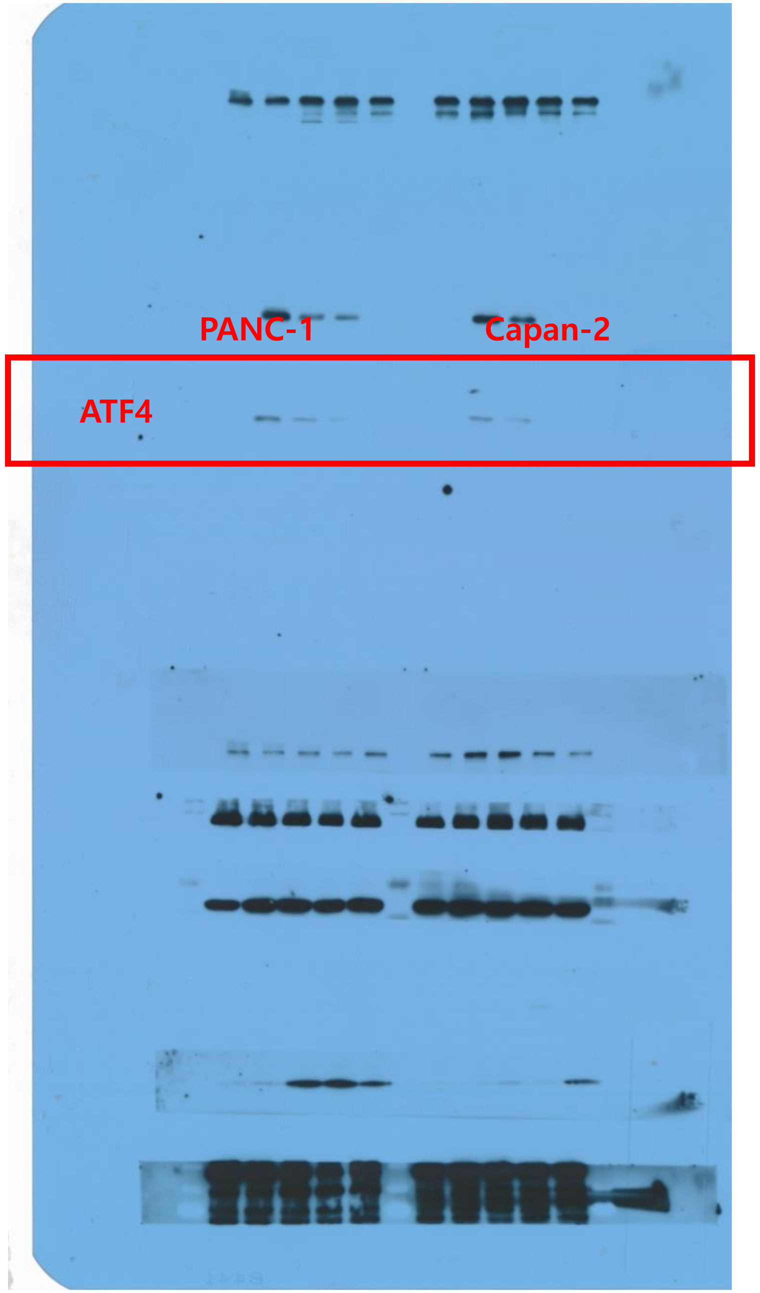

Figure 2D

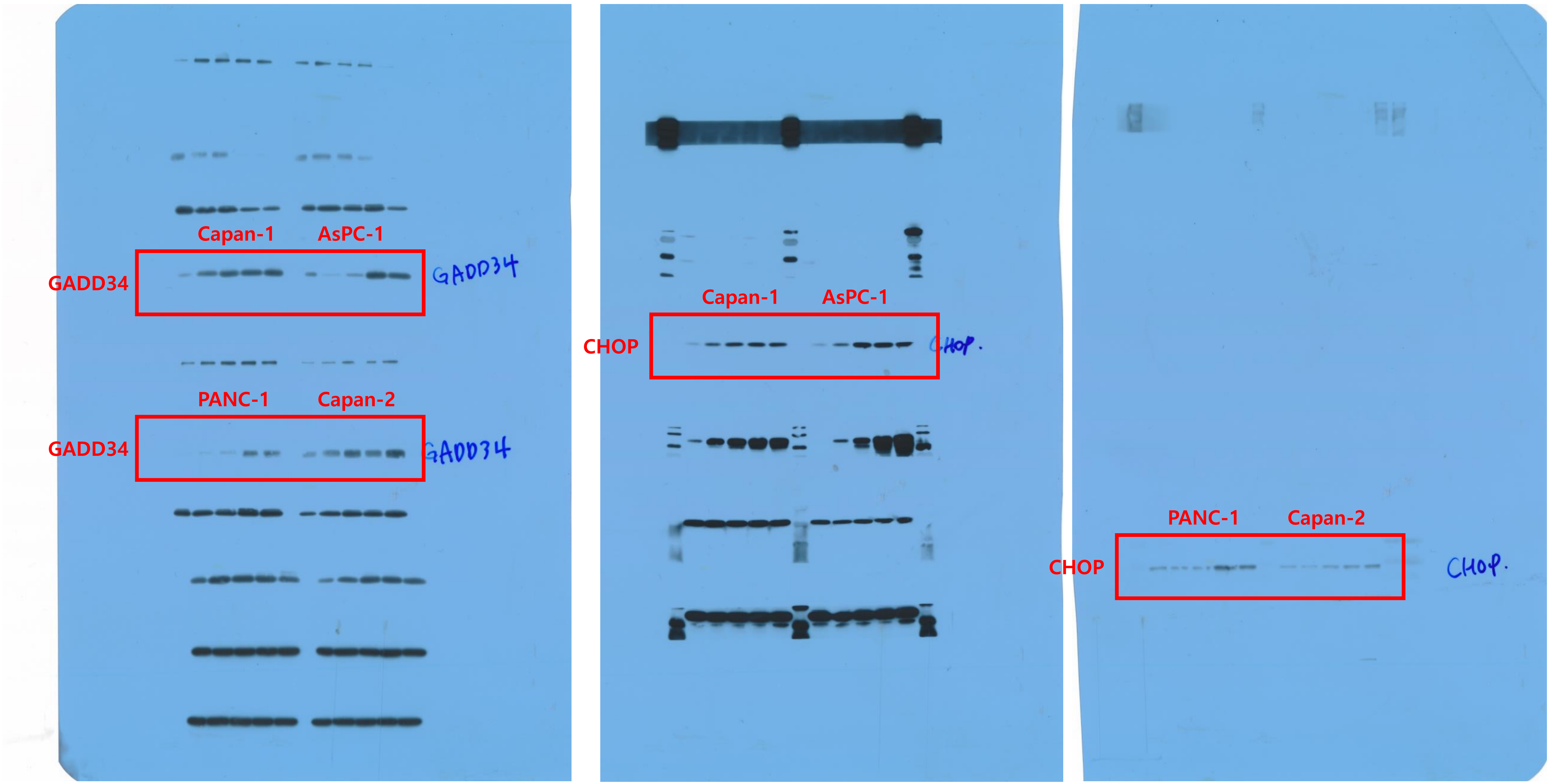
Capan-1
AsPC-1
GADD34
GADD34
PANC-1
Capan-2
GADD34
GADD34
Capan-1
AsPC-1
CHOP
CHOP.
PANC-1
Capan-2
CHOP
CHOP.

Figure 2D

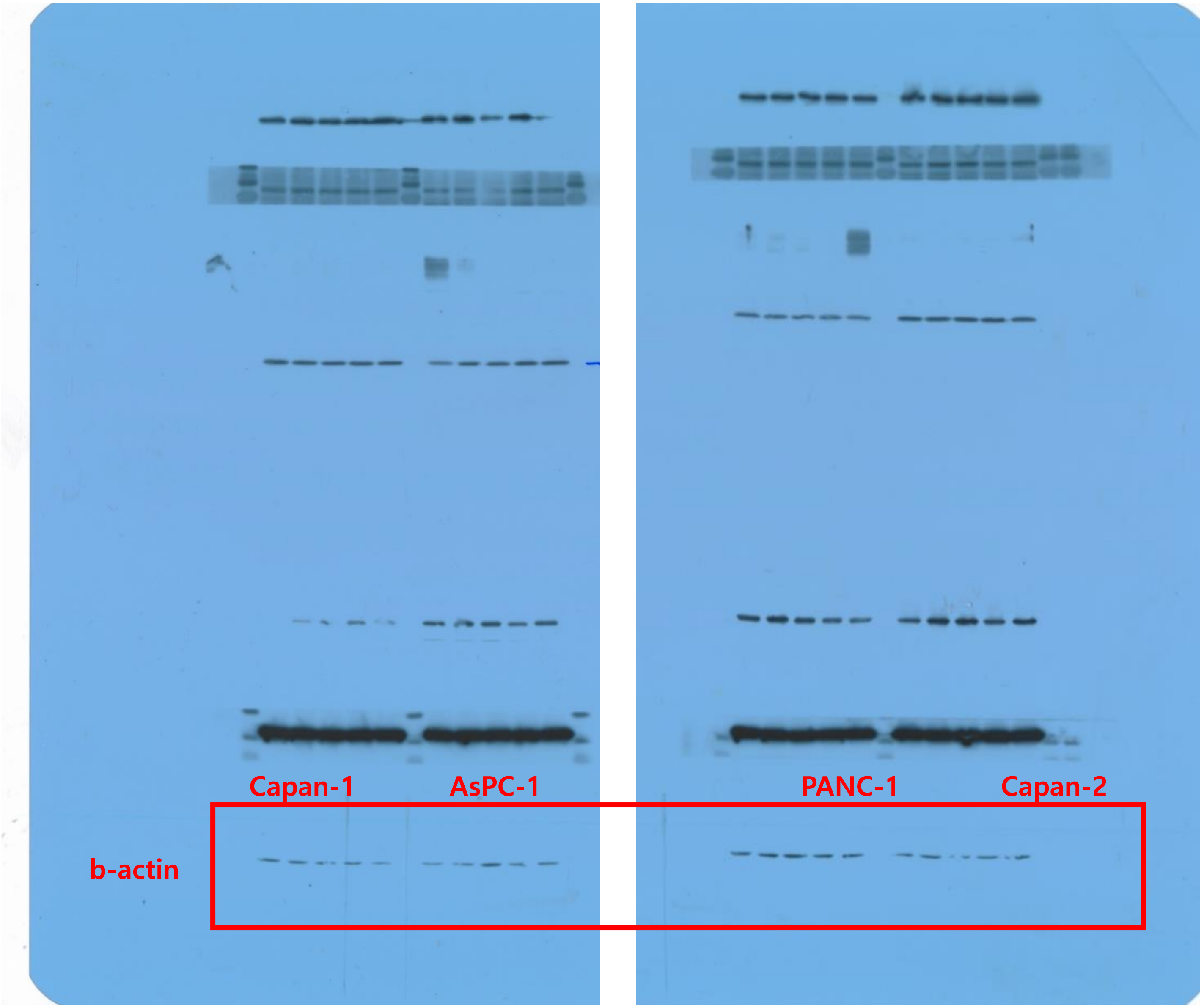

Figure 2E

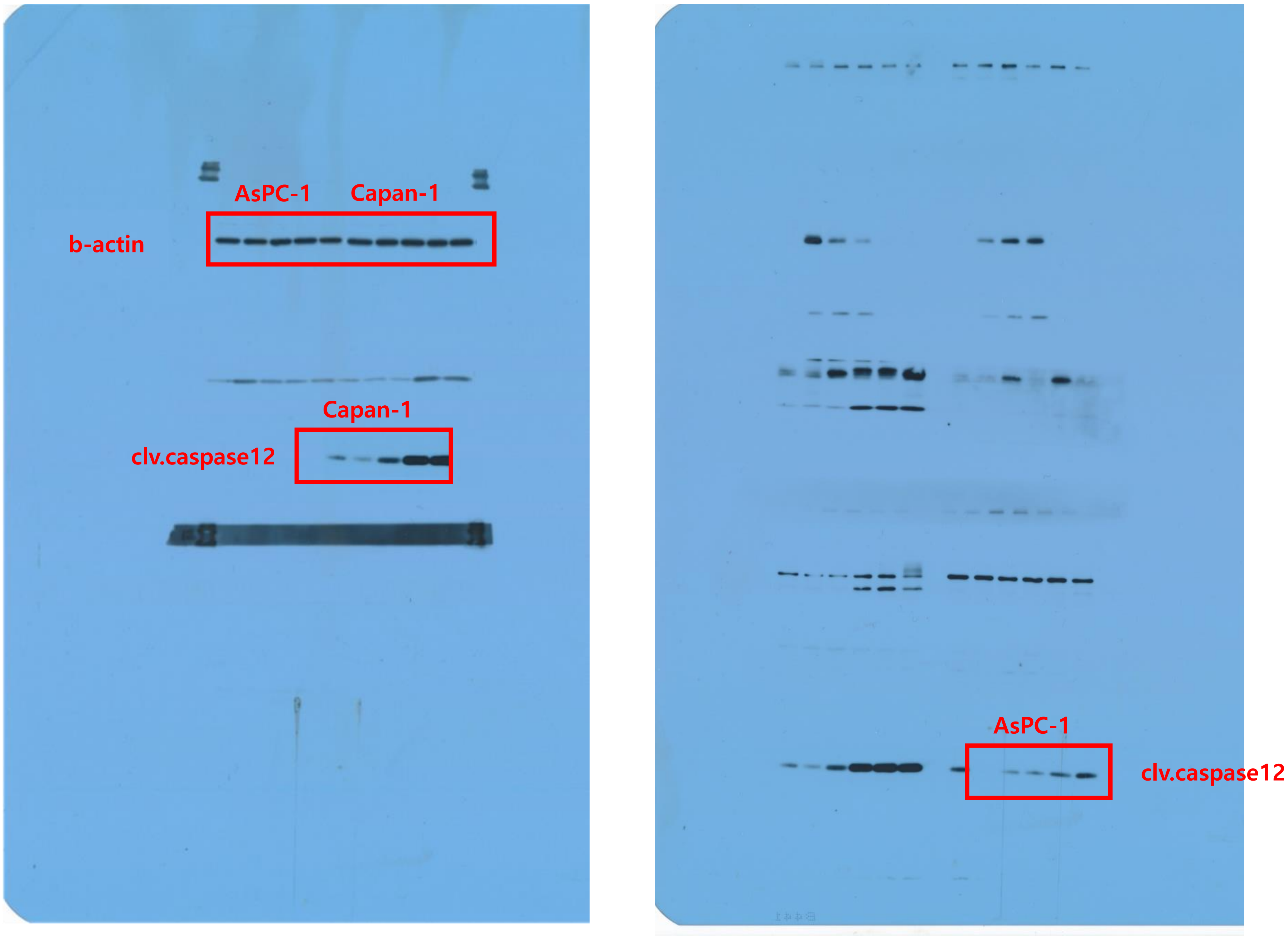
AsPC-1
Capan-1
b-actin
Capan-1
clv.caspase12
AsPC-1
clv.caspase12

Figure 2E

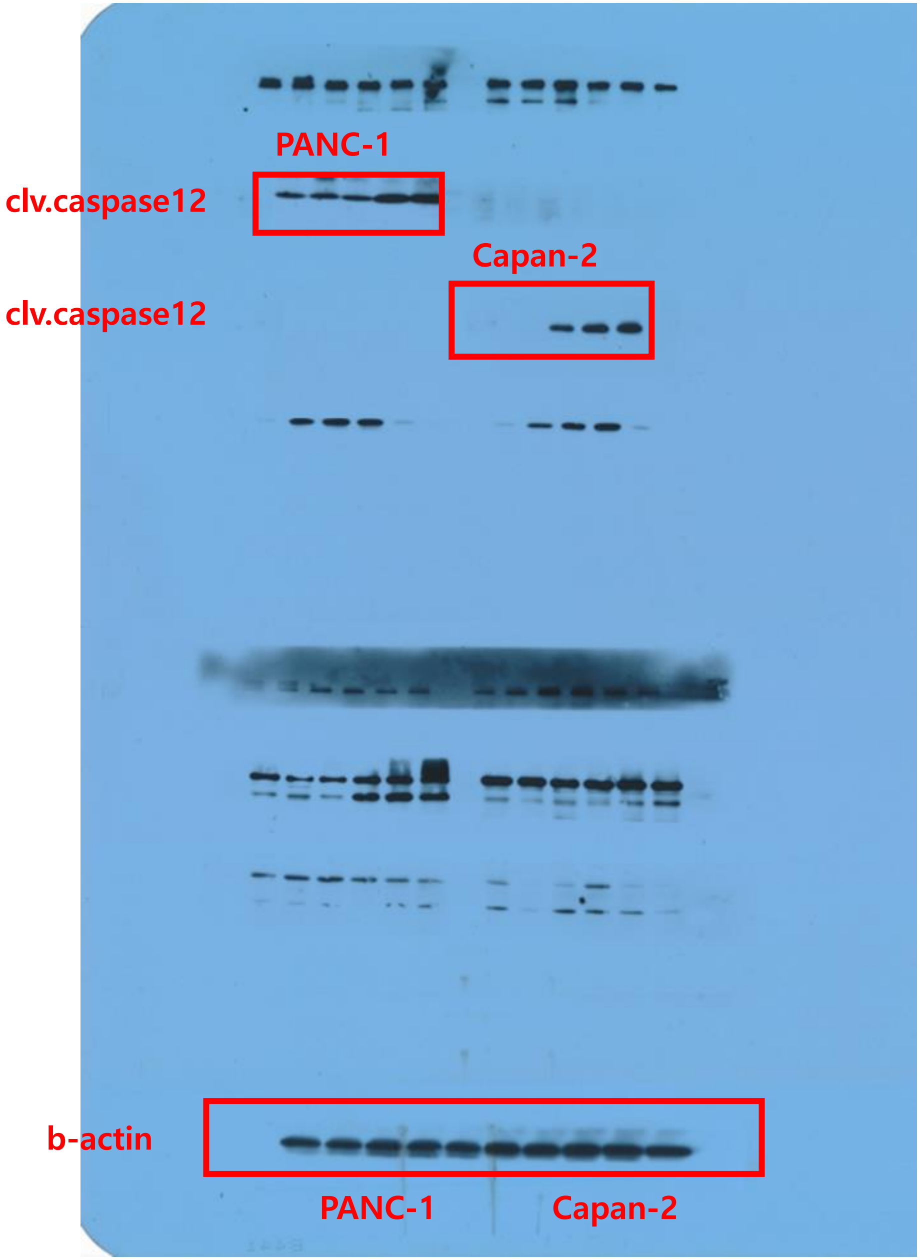
PANC-1
clv.caspase12
Capan-2
clv.caspase12
b-actin
PANC-1
Capan-2

Figure 2G

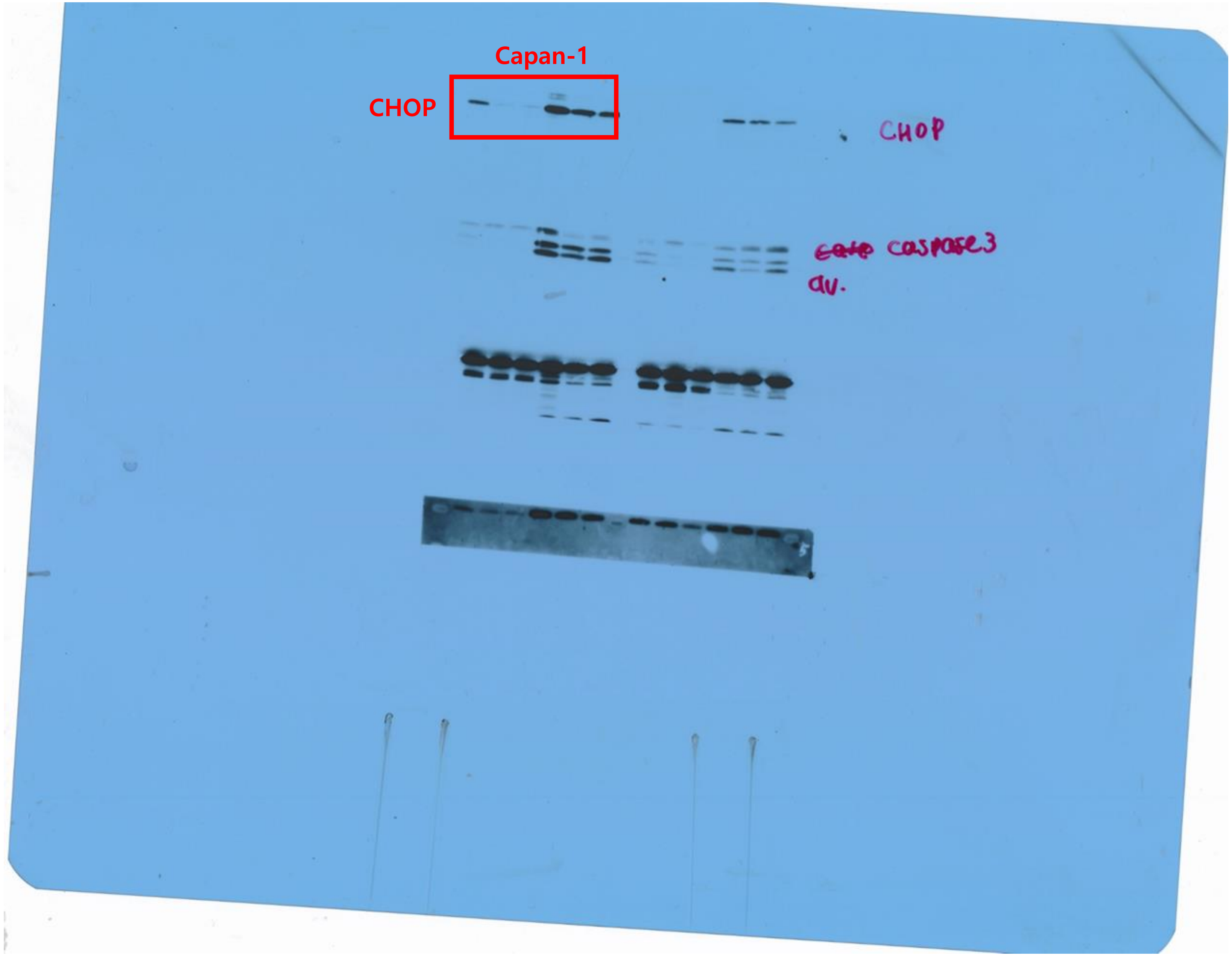

Figure 2G

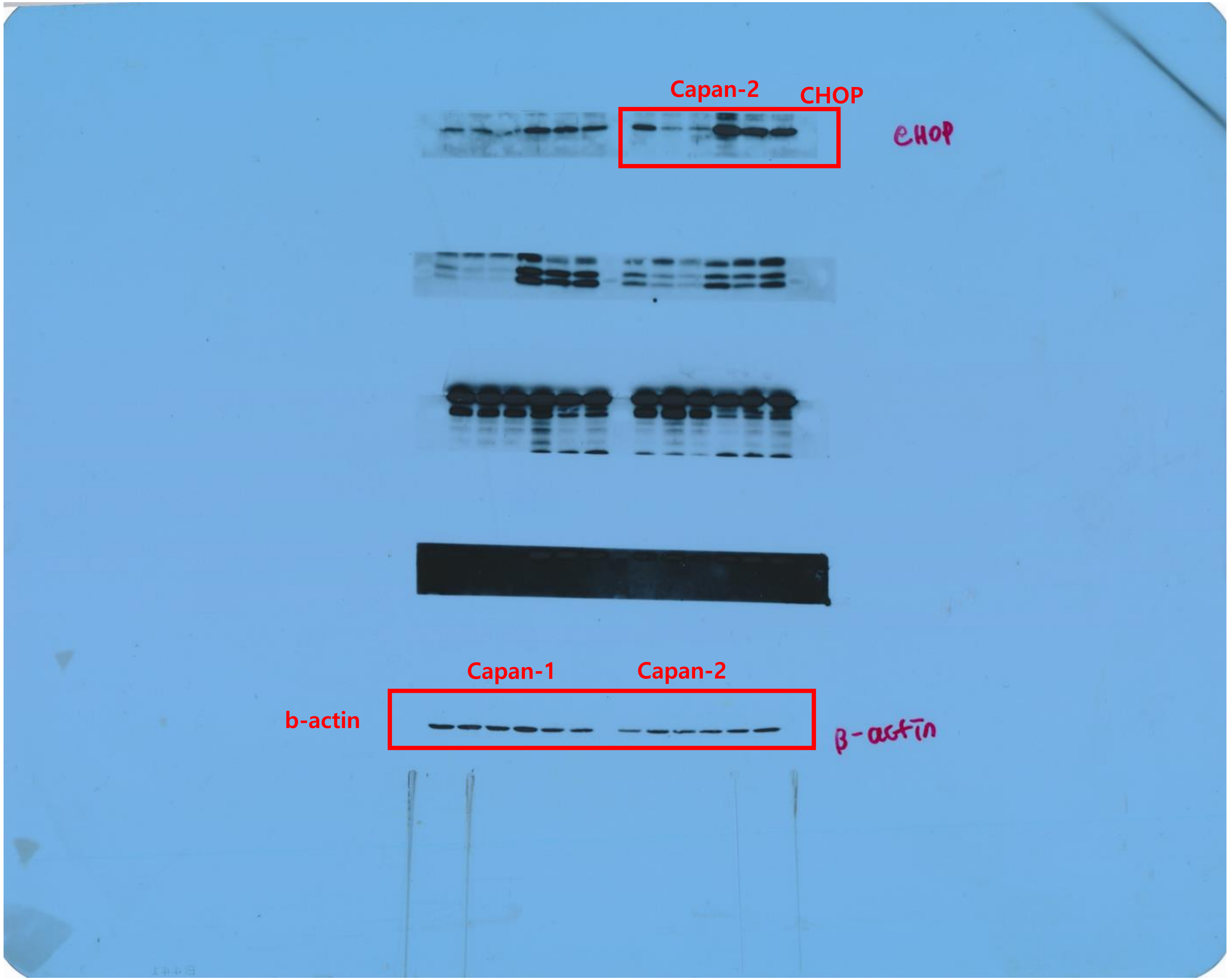

Figure 3C

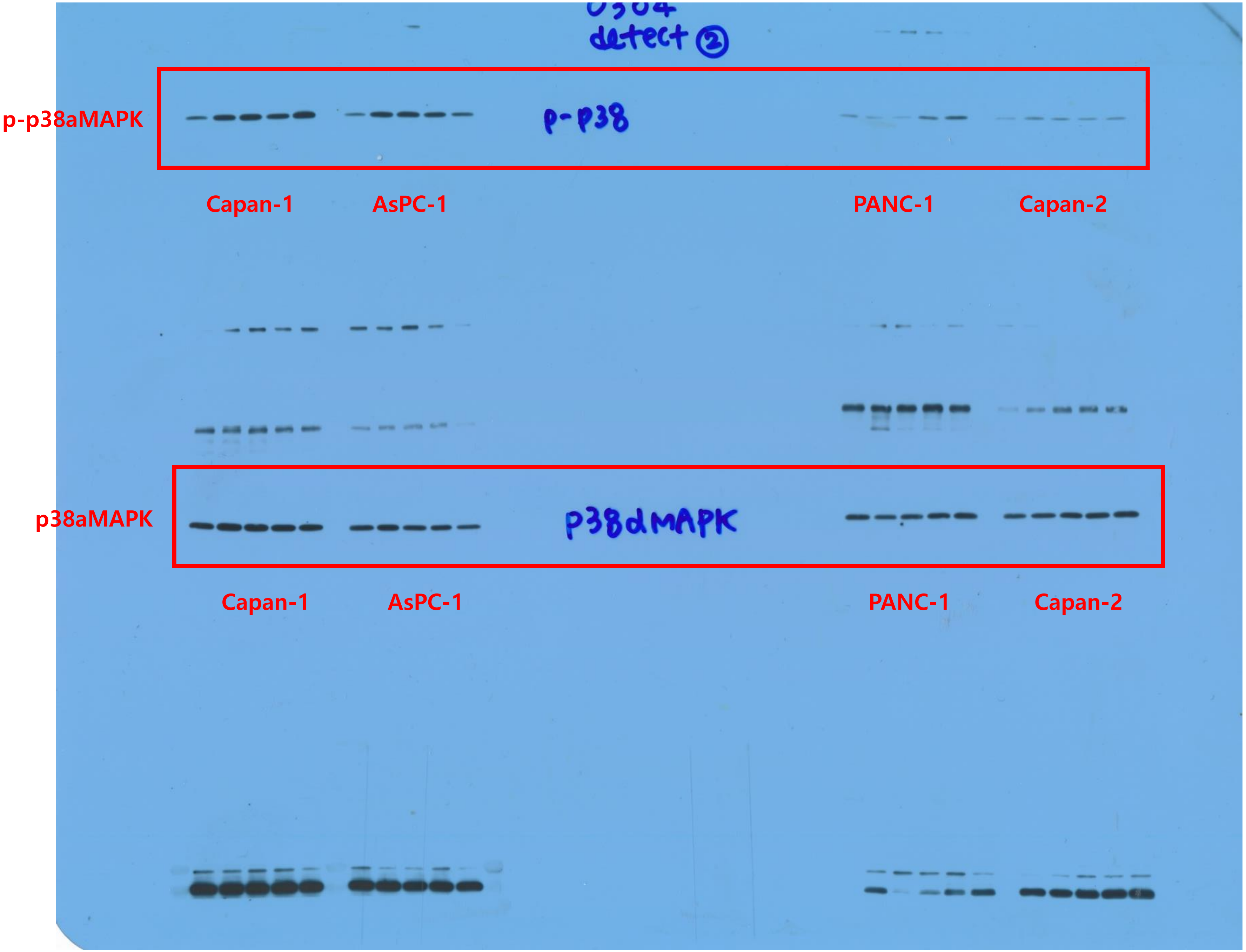

0304
detect ②
p-p38aMAPK
p-p38
Capan-1
AsPC-1
PANC-1
Capan-2
p38aMAPK
p38αMAPK
Capan-1
AsPC-1
PANC-1
Capan-2

Figure 3C

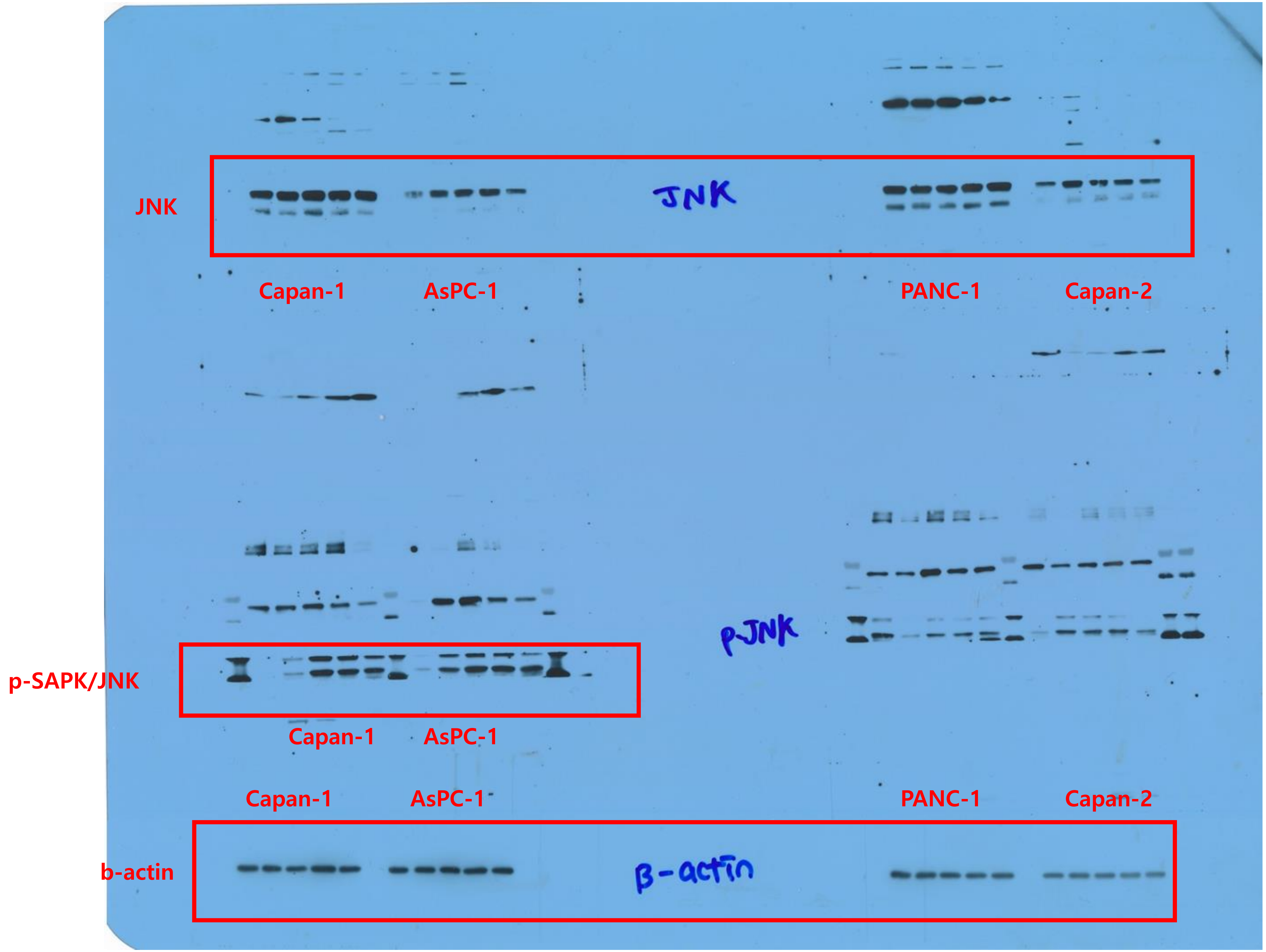

JNK
JNK
Capan-1
AsPC-1
PANC-1
Capan-2
p-JNK
p-SAPK/JNK
Capan-1
AsPC-1
Capan-1
AsPC-1
PANC-1
Capan-2
b-actin
β-actin

Figure 3C

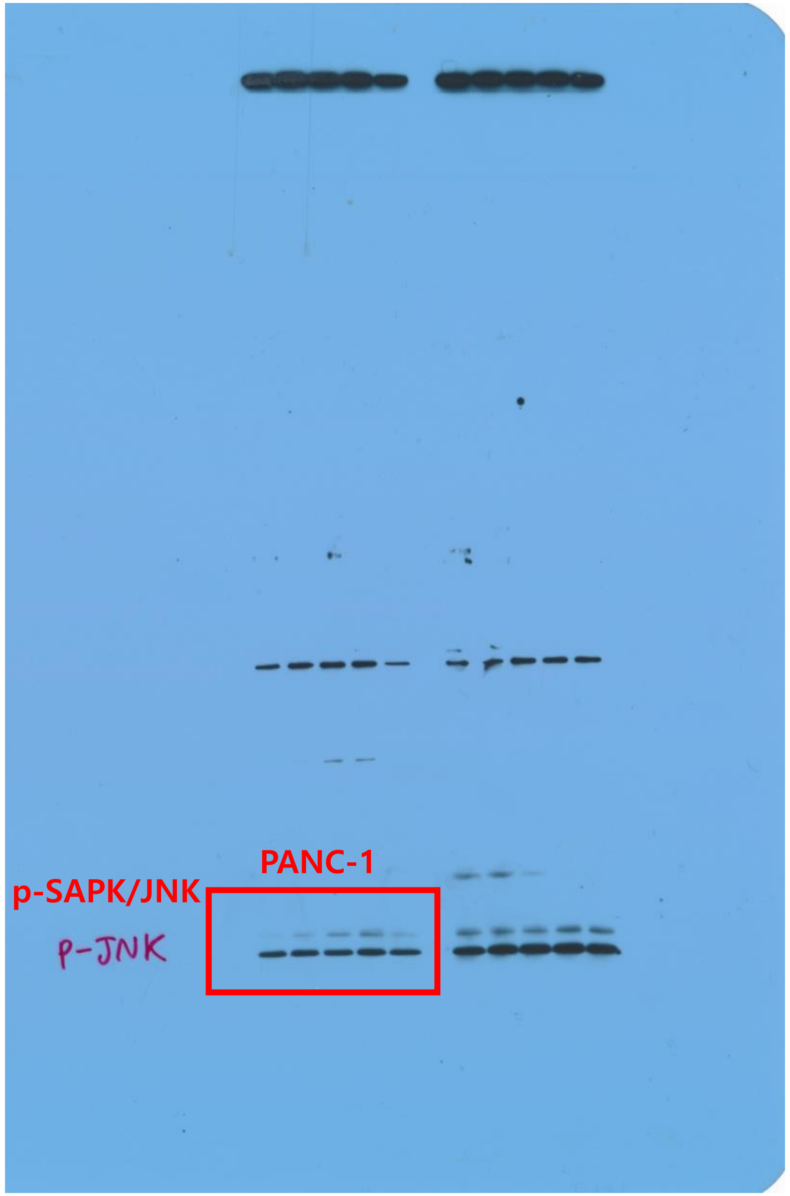

Figure 3C

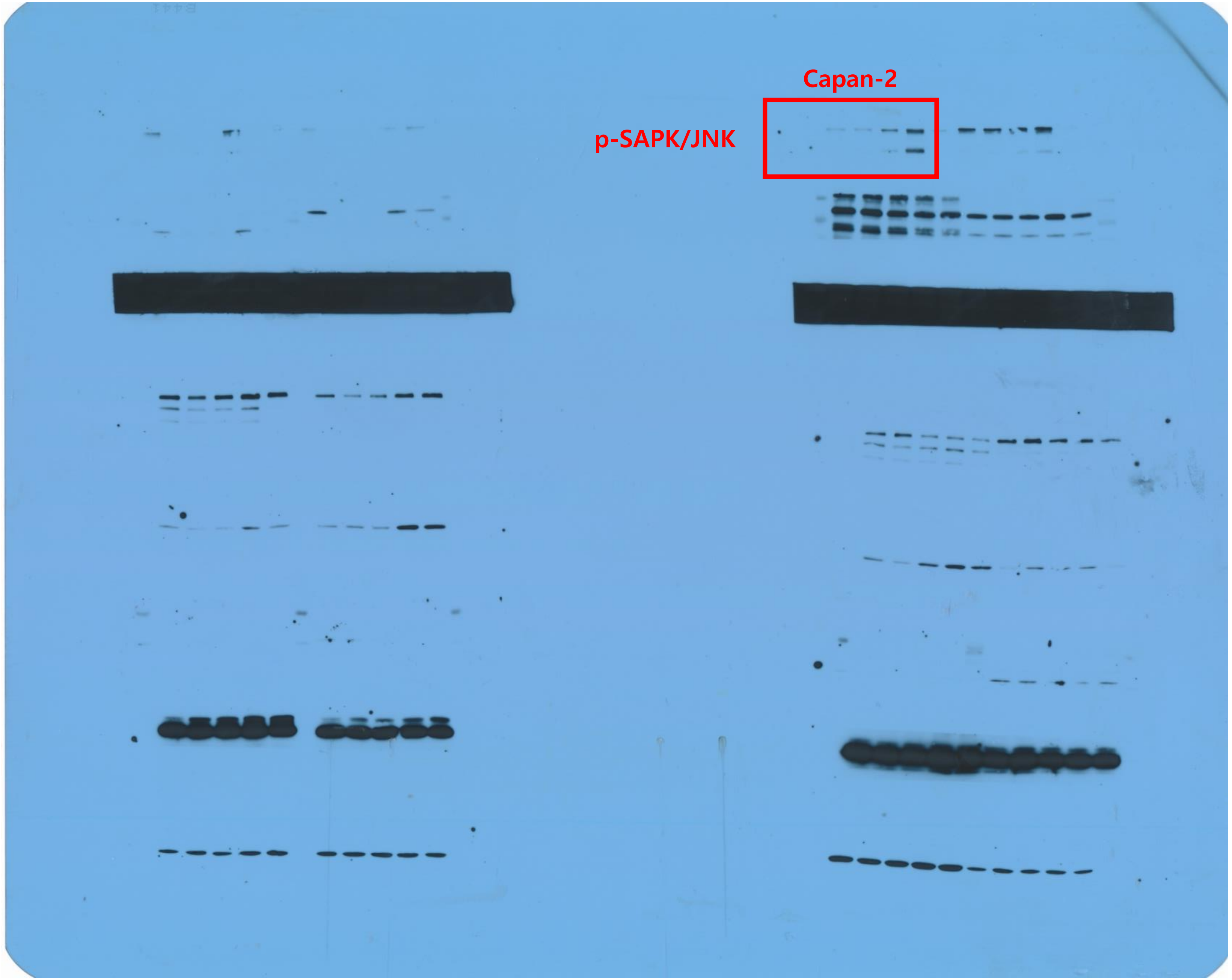

Figure 3D

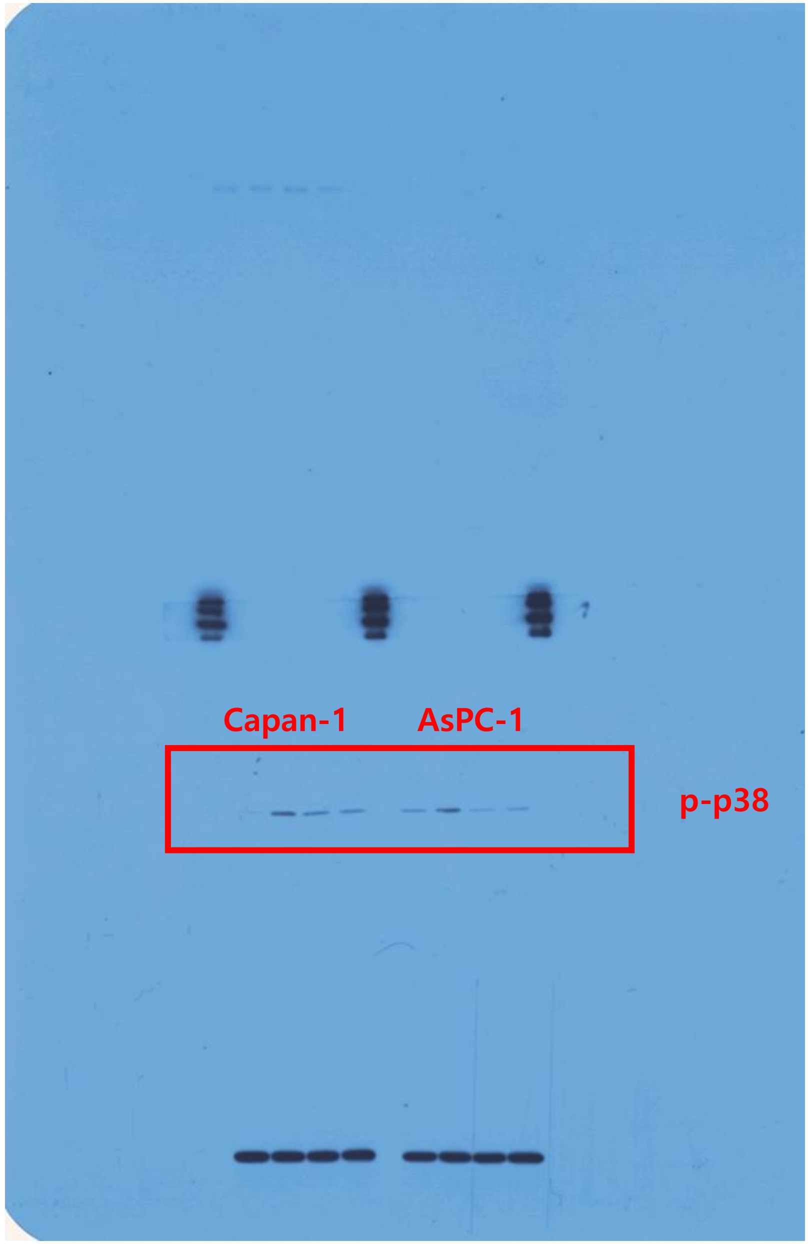

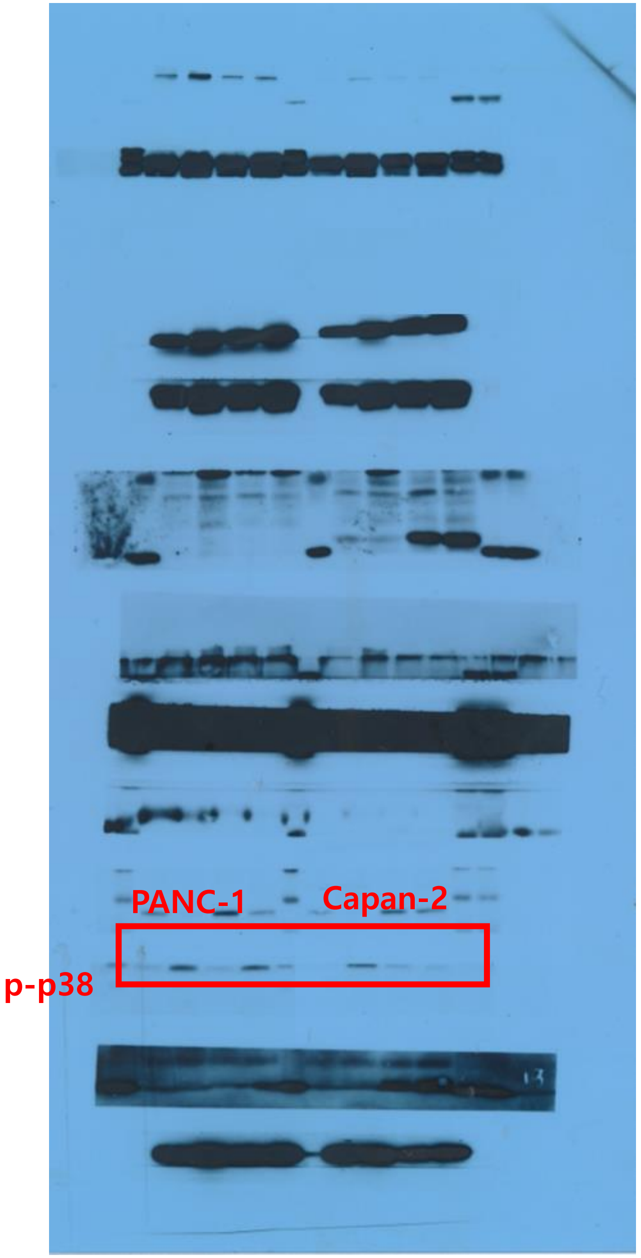

Figure 3D

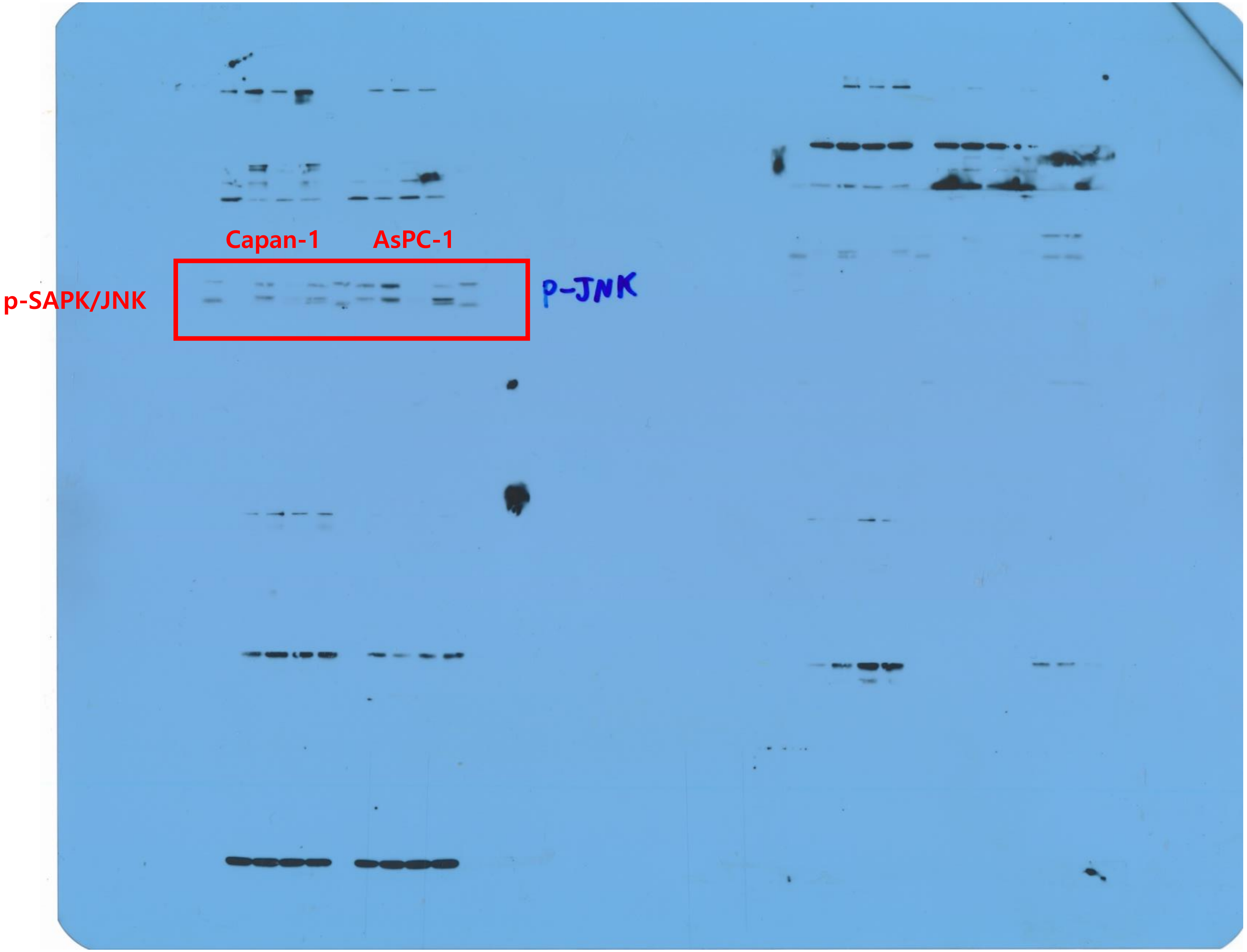

Figure 3D

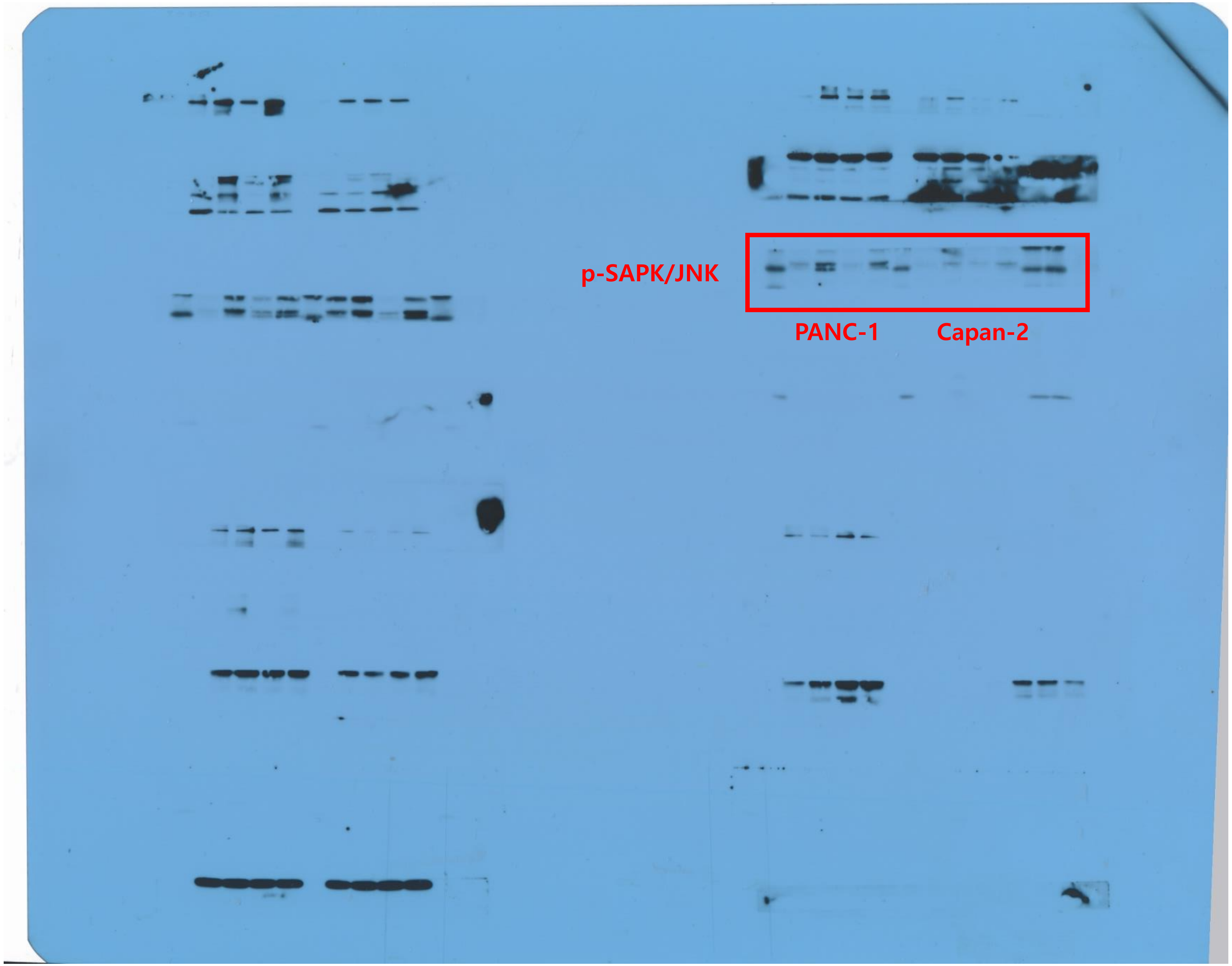

Figure 3D

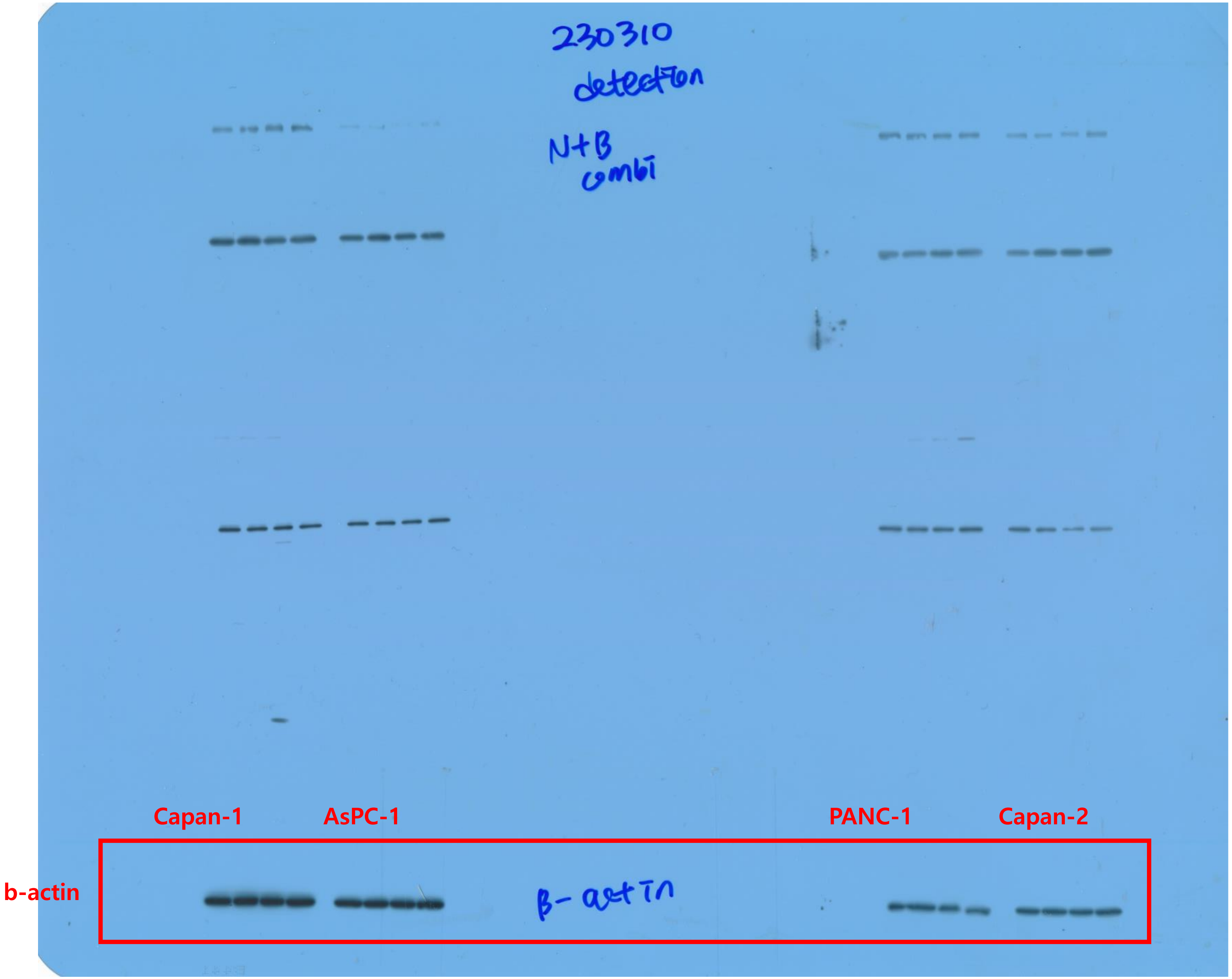

230310
detection
N+B
combi
Capan-1
AsPC-1
PANC-1
Capan-2
b-actin
β-actin

Figure 4A

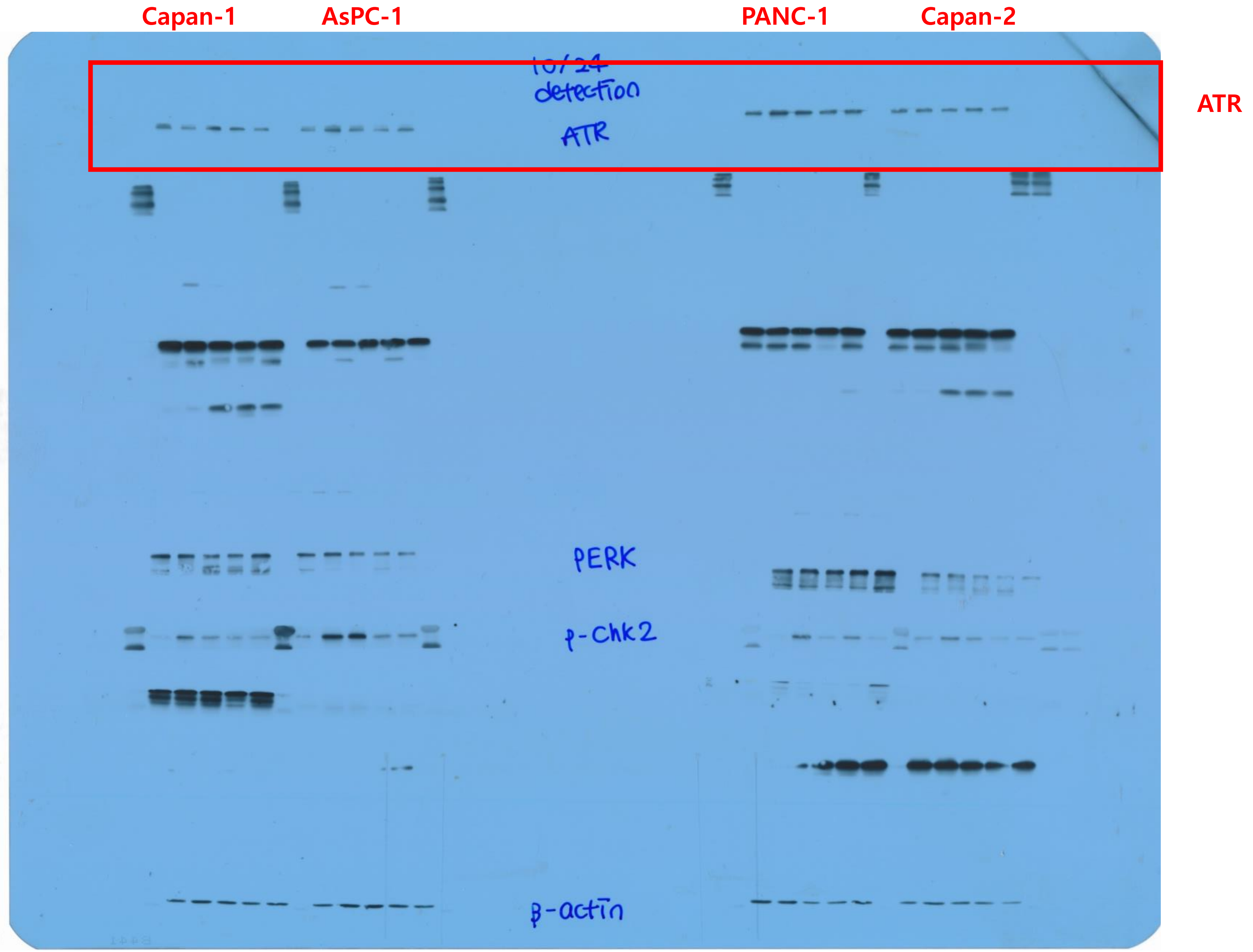

Capan-1
AsPC-1
PANC-1
Capan-2
ATR
10/24
detection
ATR
PERK
p-Chk2
β-actin

Figure 4A

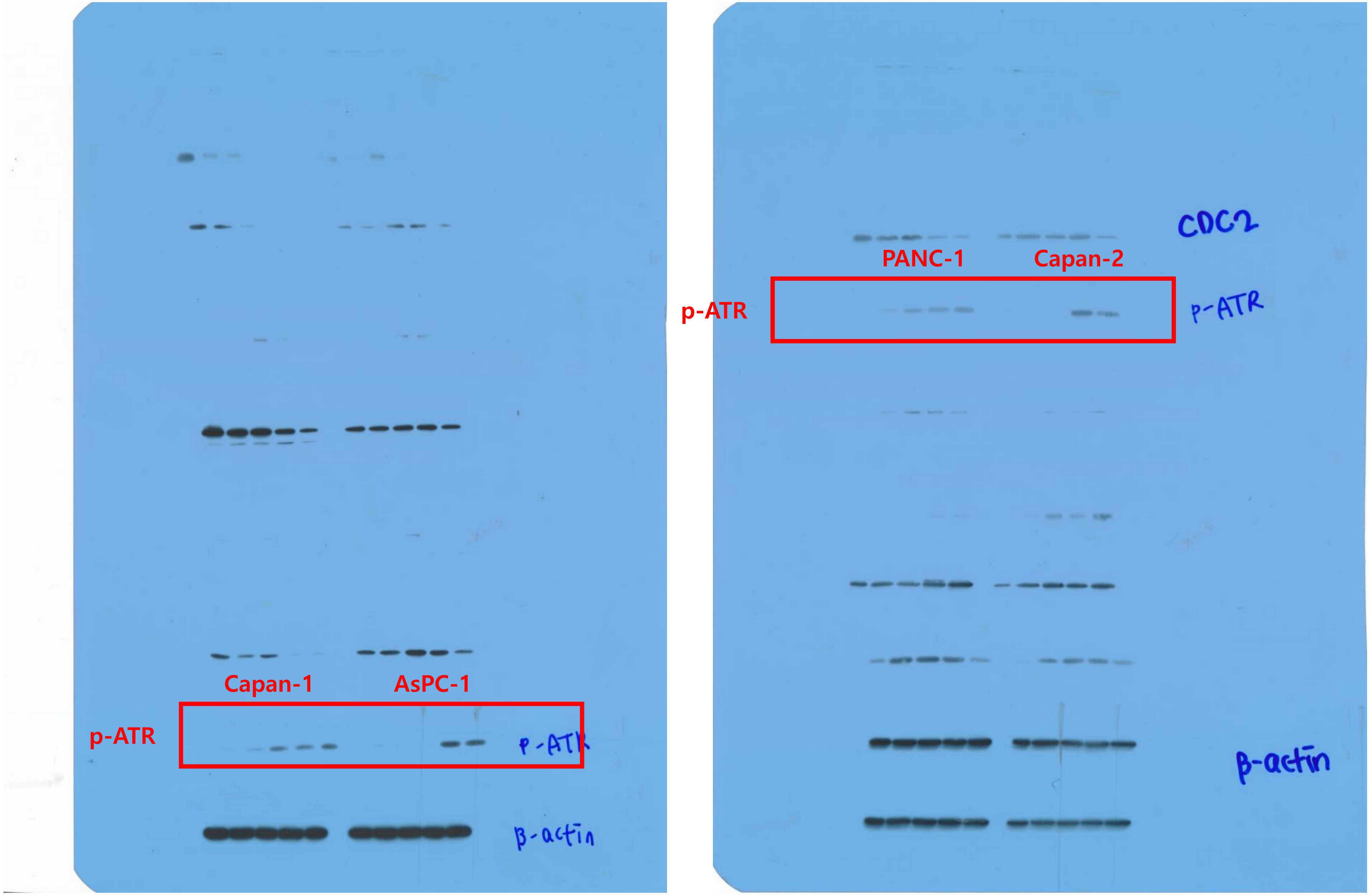

p-ATR
Capan-1
AsPC-1
p-ATR
β-actin
CDC2
PANC-1
Capan-2
p-ATR
p-ATR
β-actin

Figure 4A

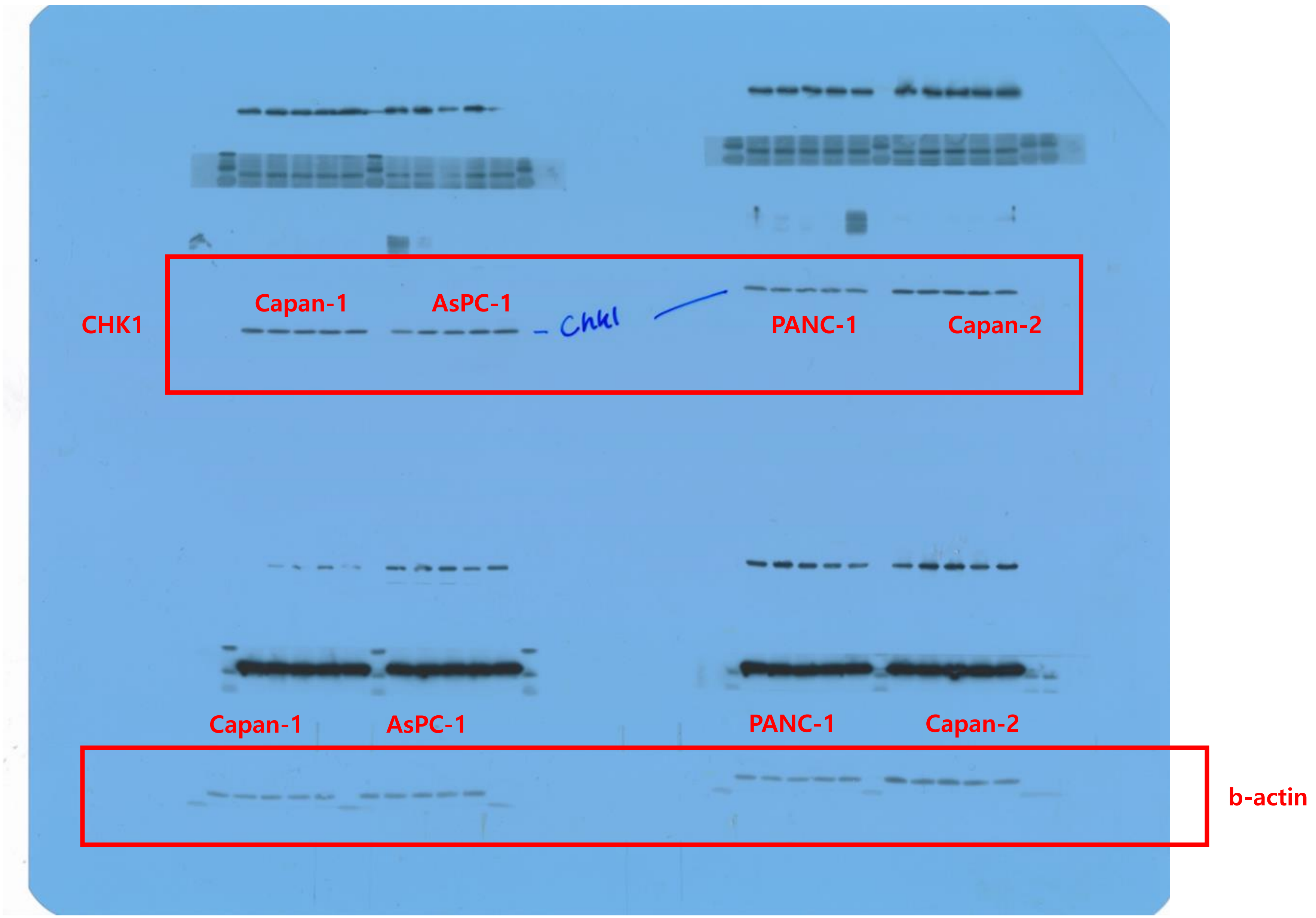

CHK1
Capan-1
AsPC-1
Chk1
PANC-1
Capan-2
Capan-1
AsPC-1
PANC-1
Capan-2
b-actin

Figure 4A

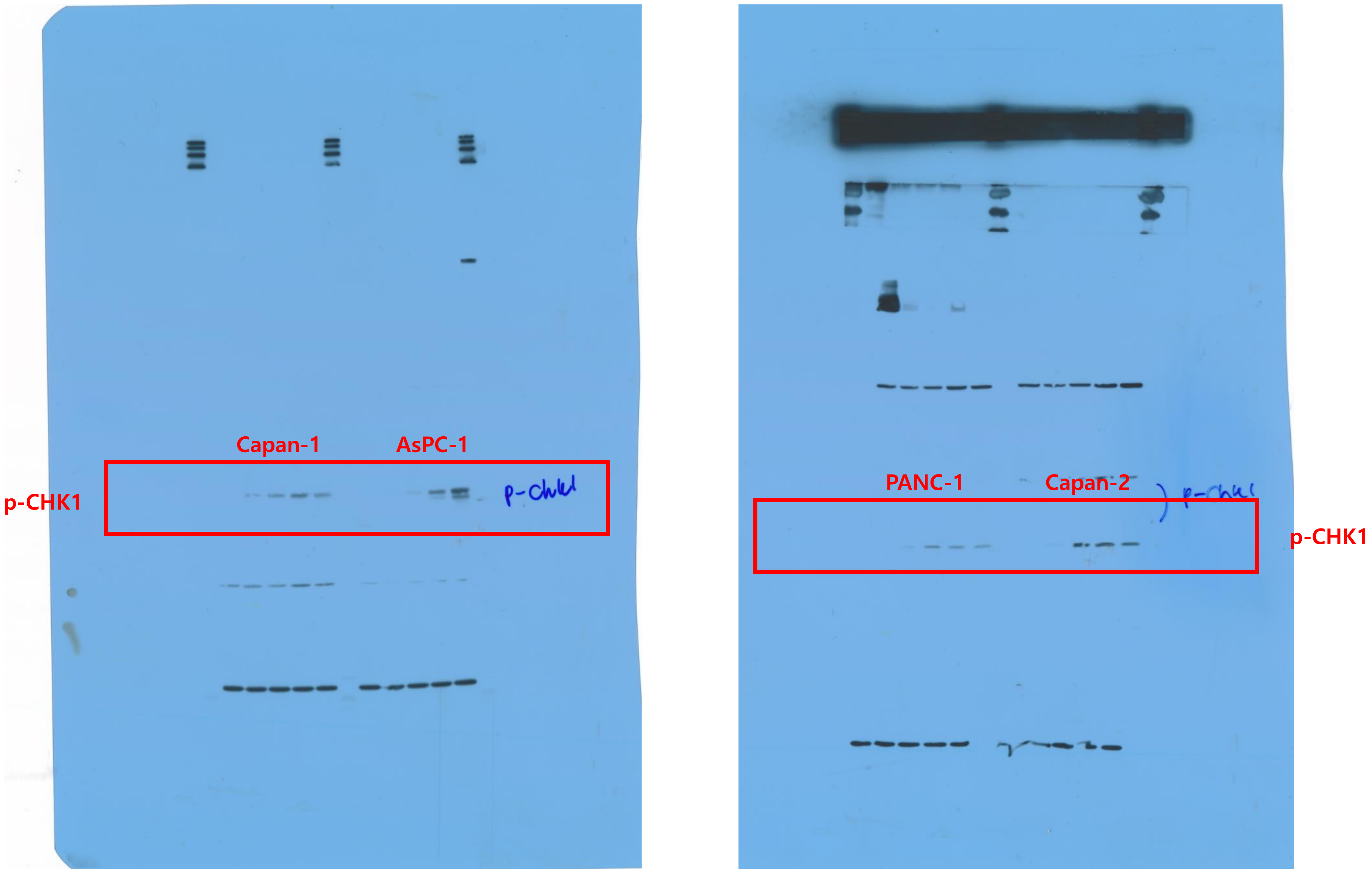

Figure 4A

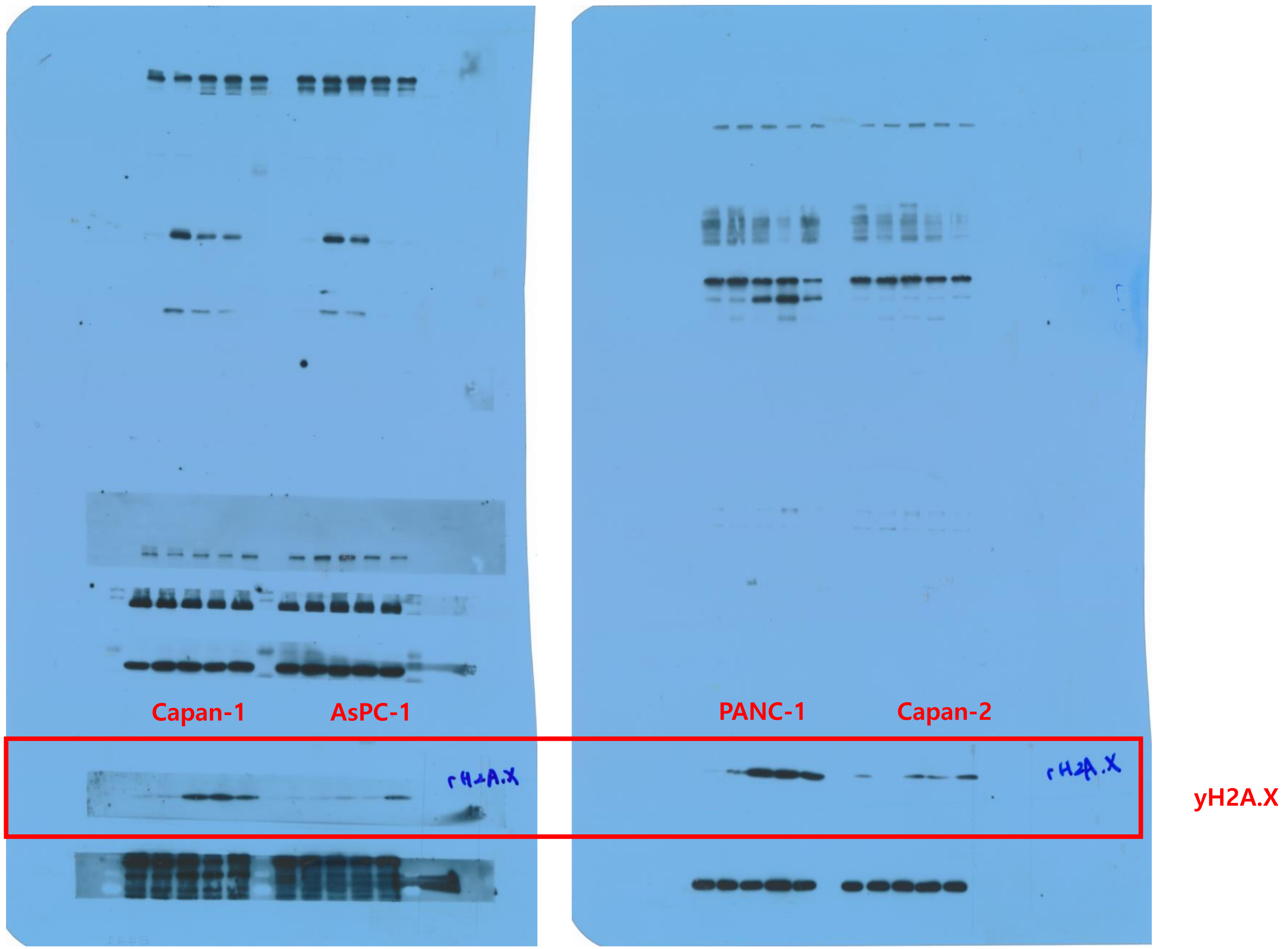

Figure 4D

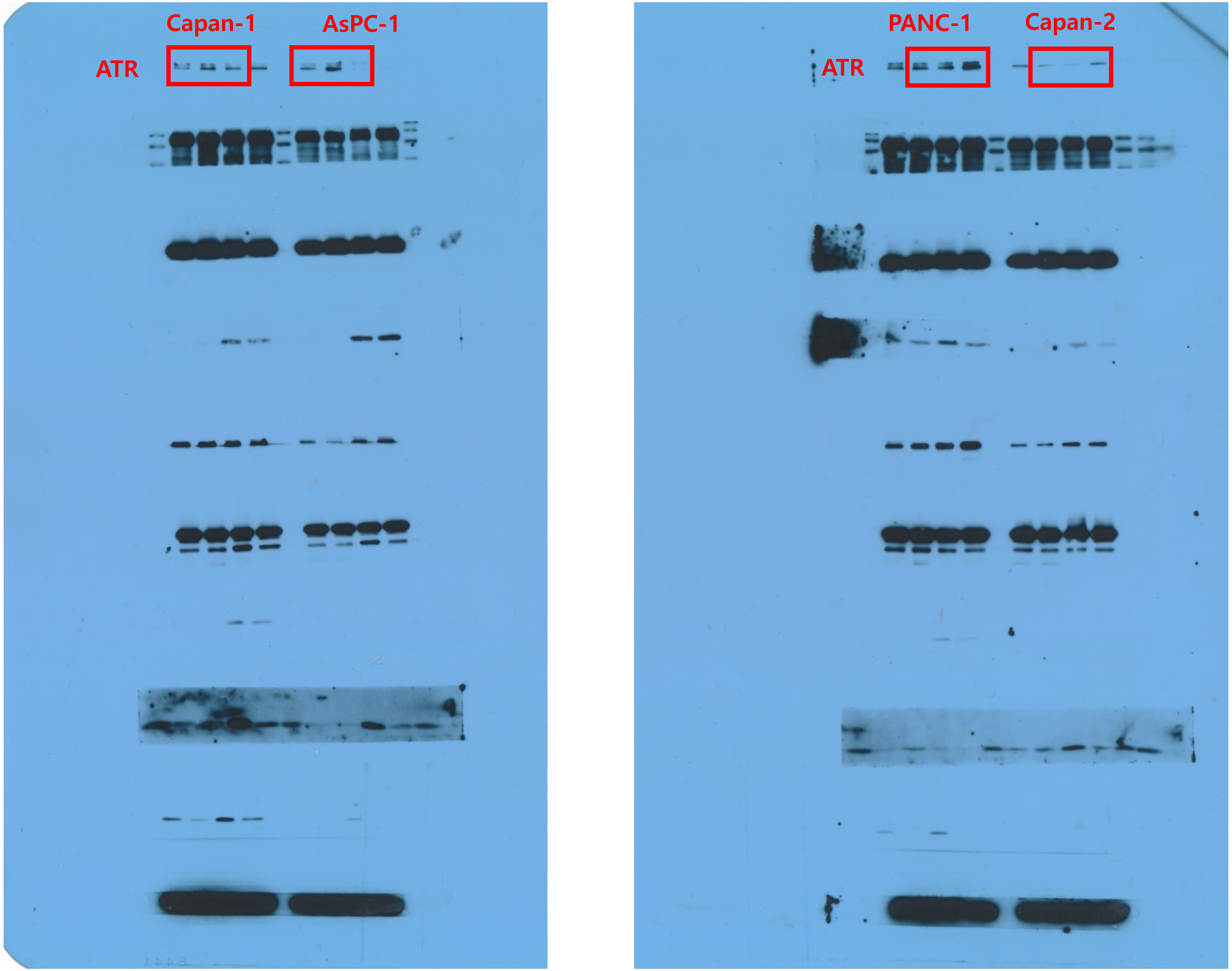

Figure 4D

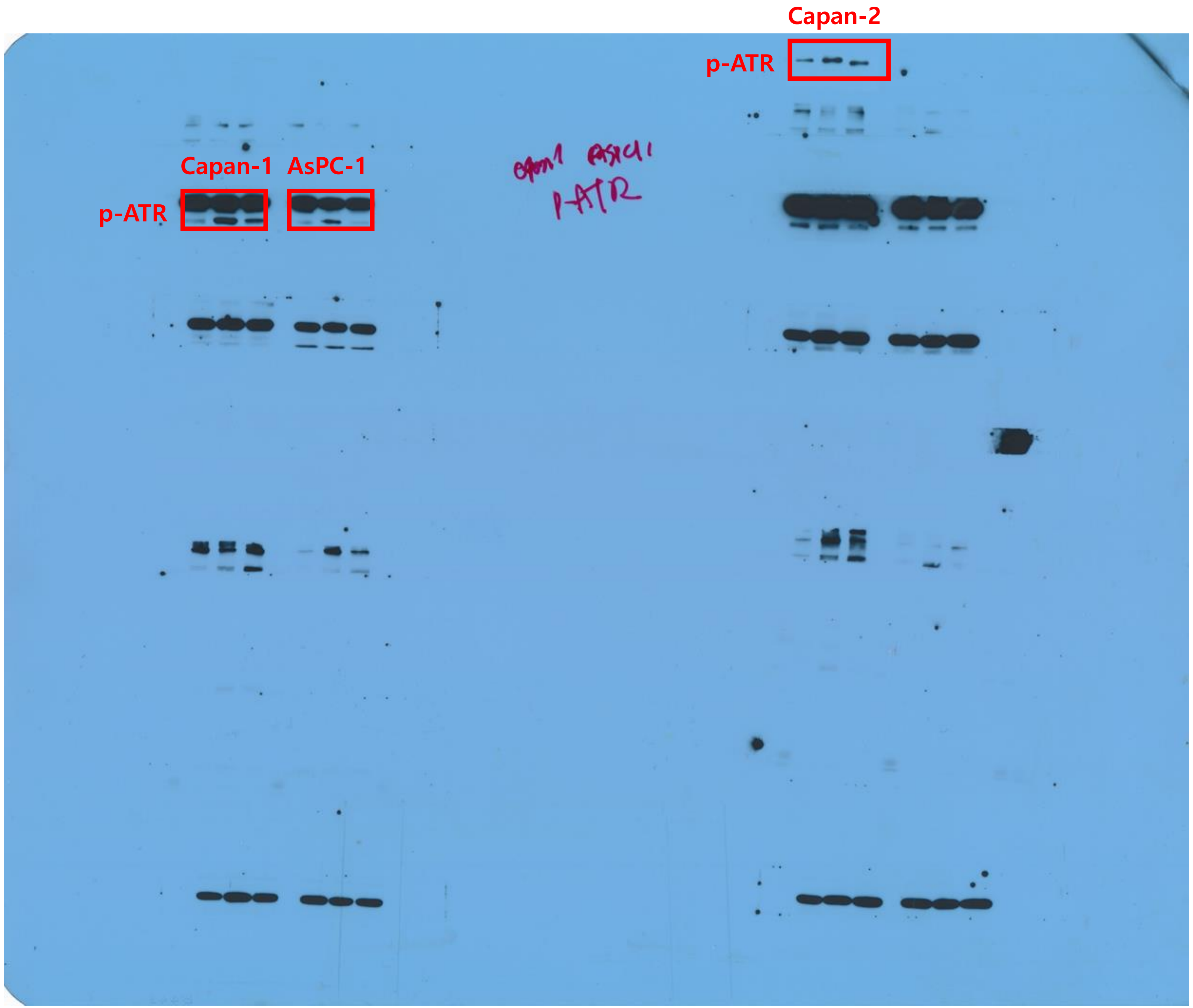

Figure 4D

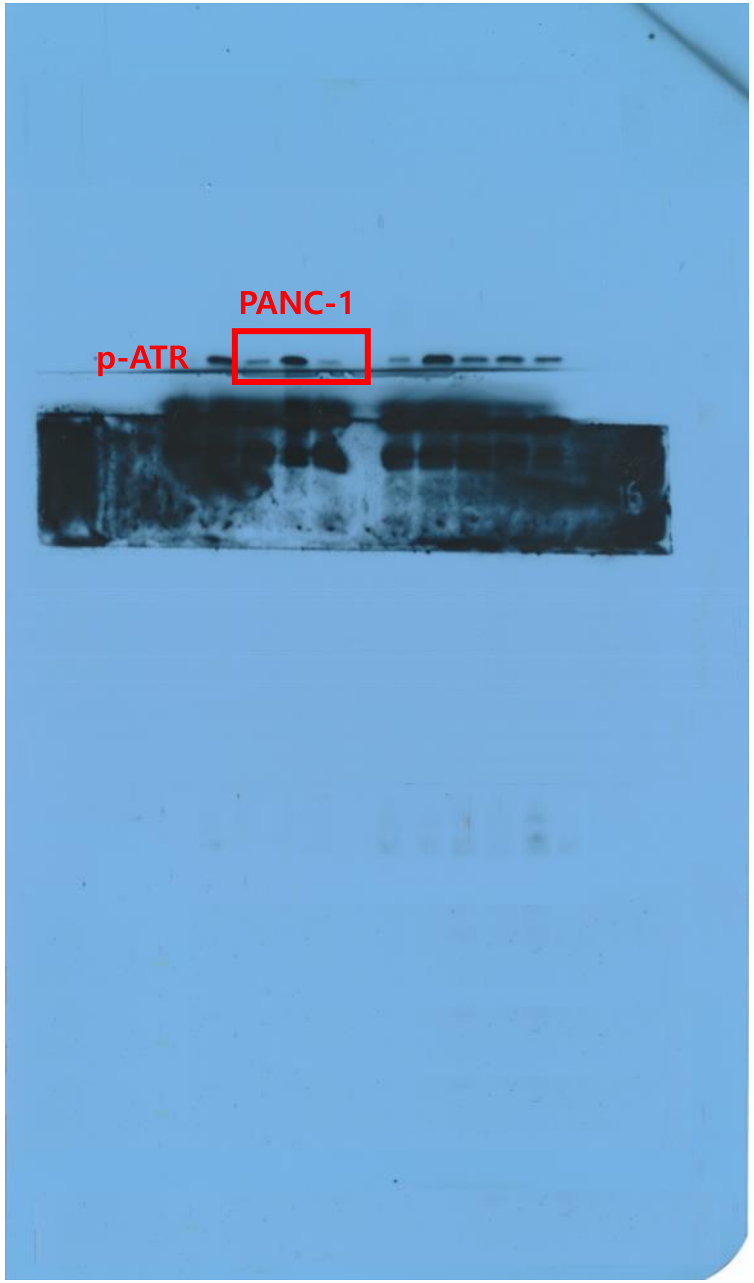

Figure 4D

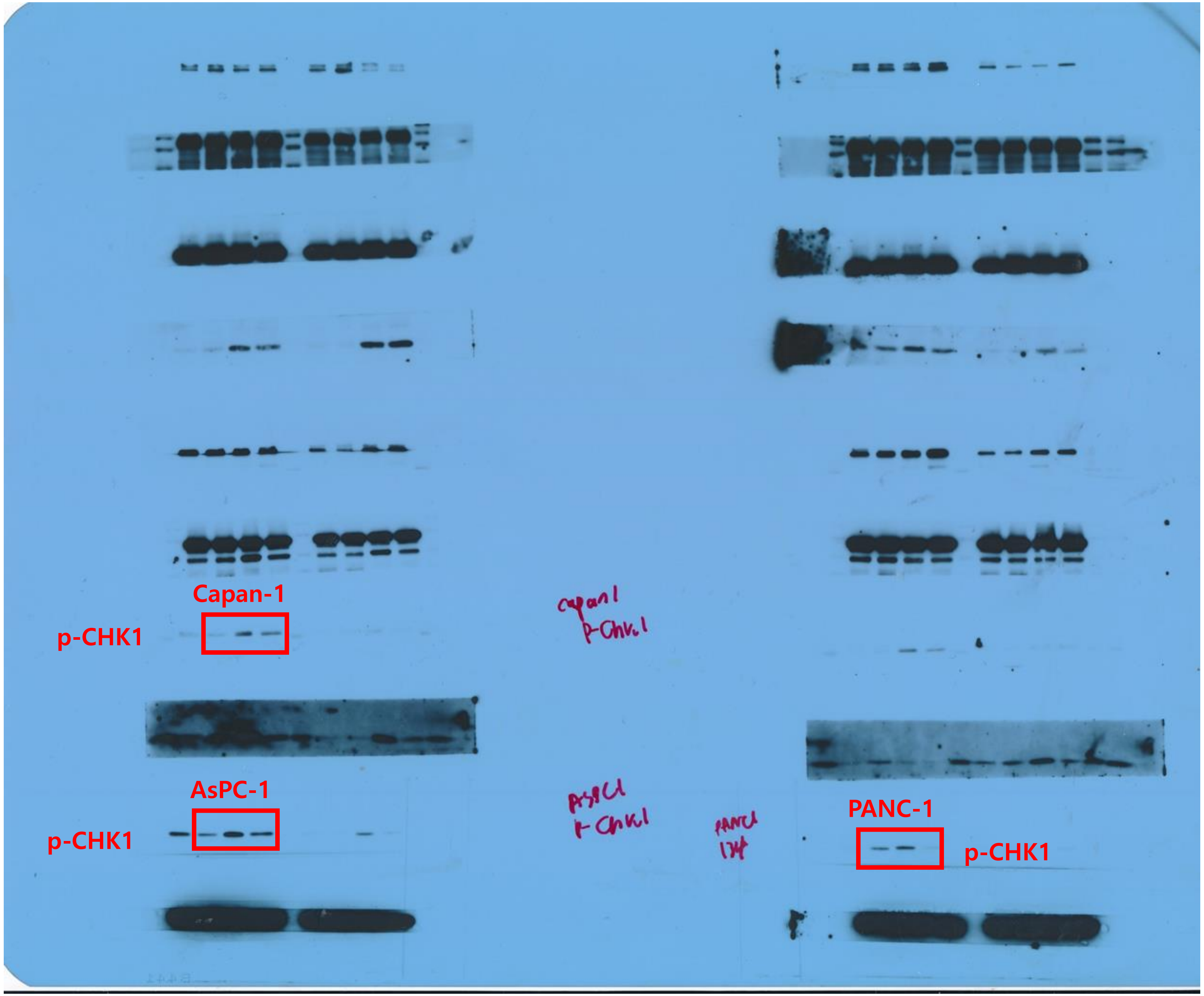

Figure 4D

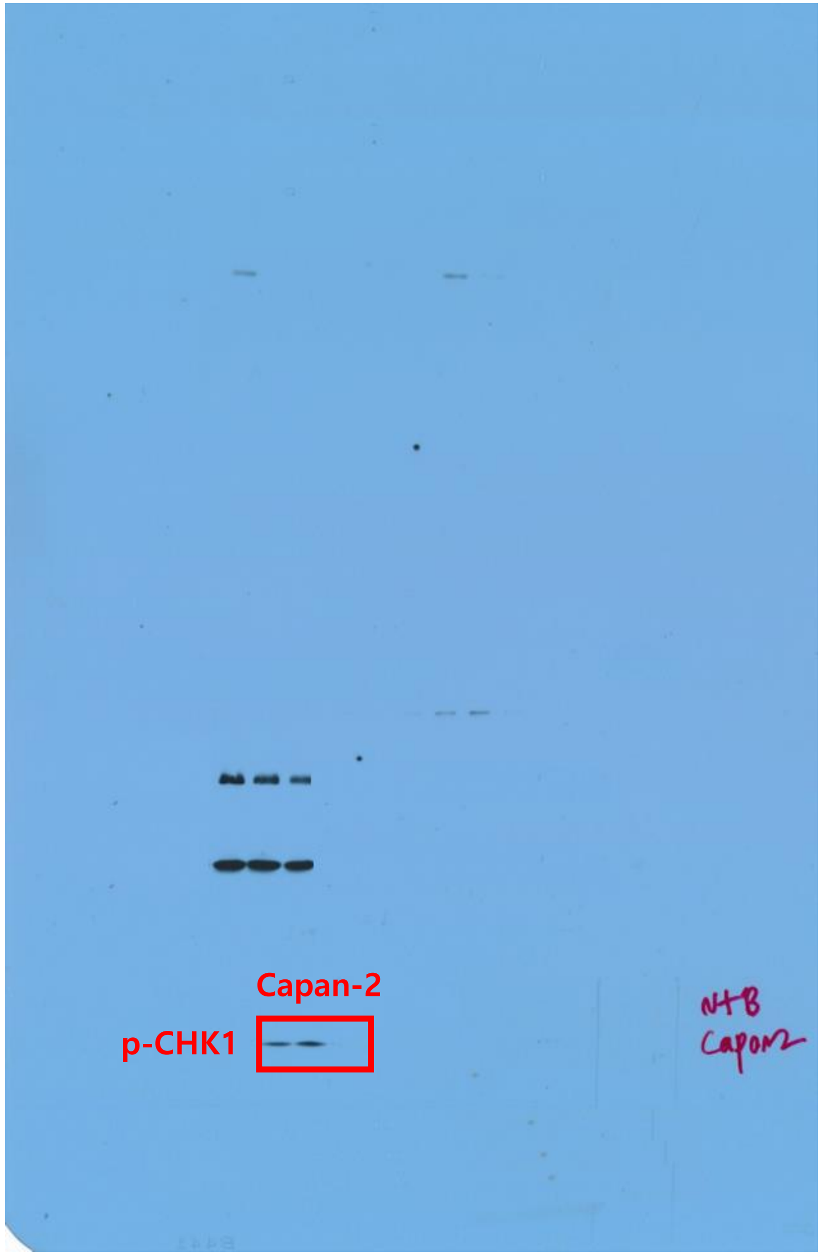

Figure 4D

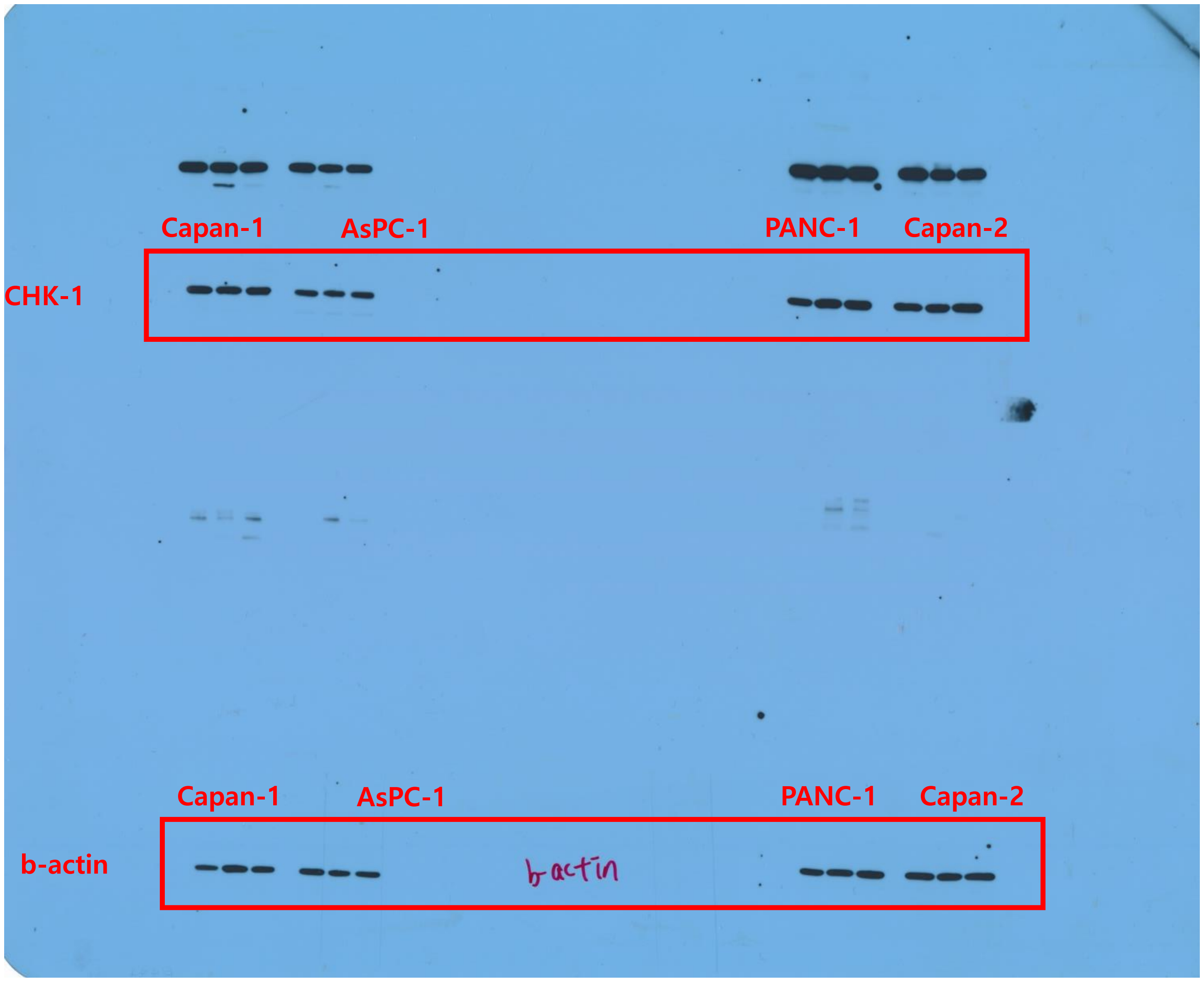
Capan-1
AsPC-1
PANC-1
Capan-2
CHK-1
Capan-1
AsPC-1
PANC-1
Capan-2
b-actin
b-actin

Figure 4F

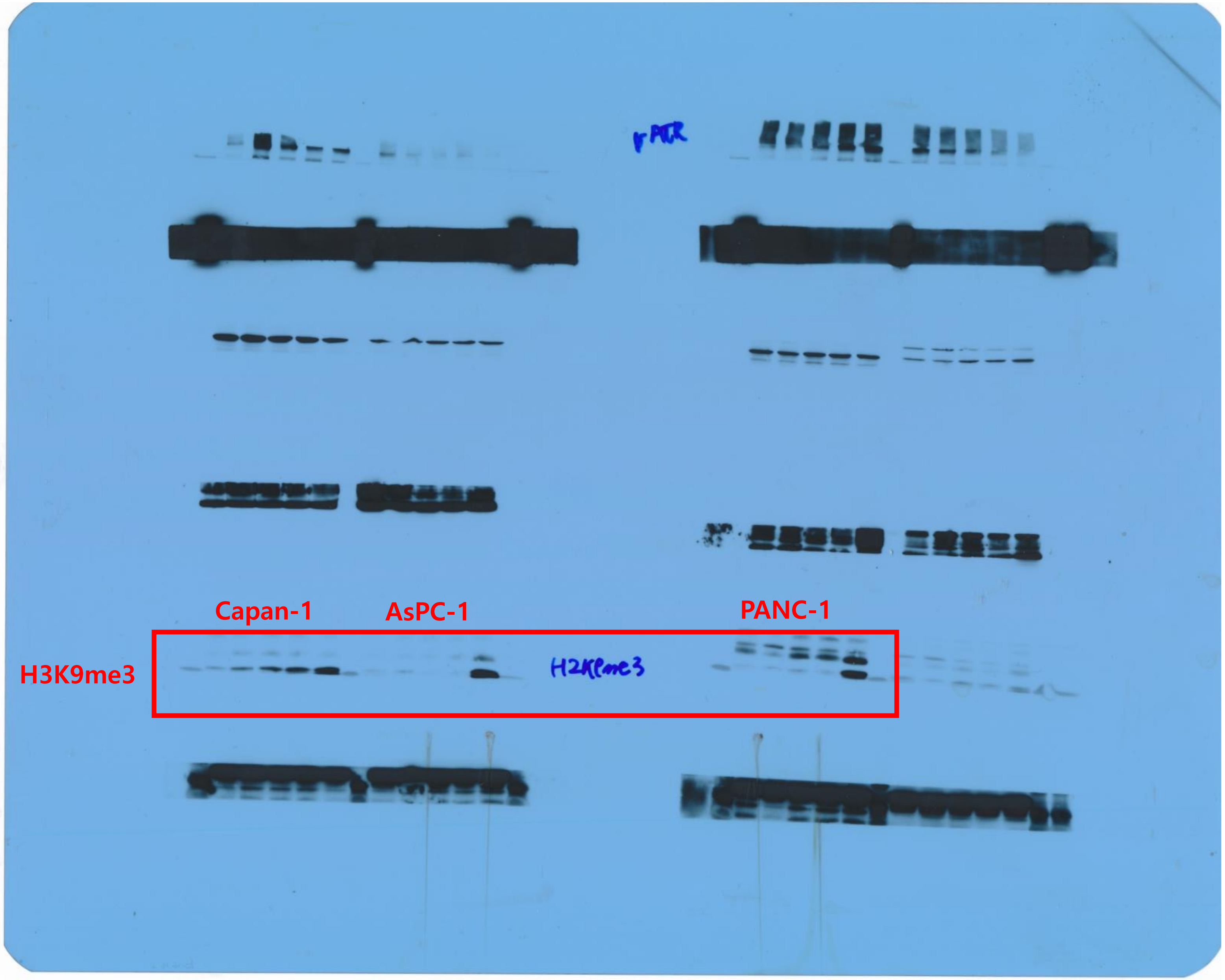

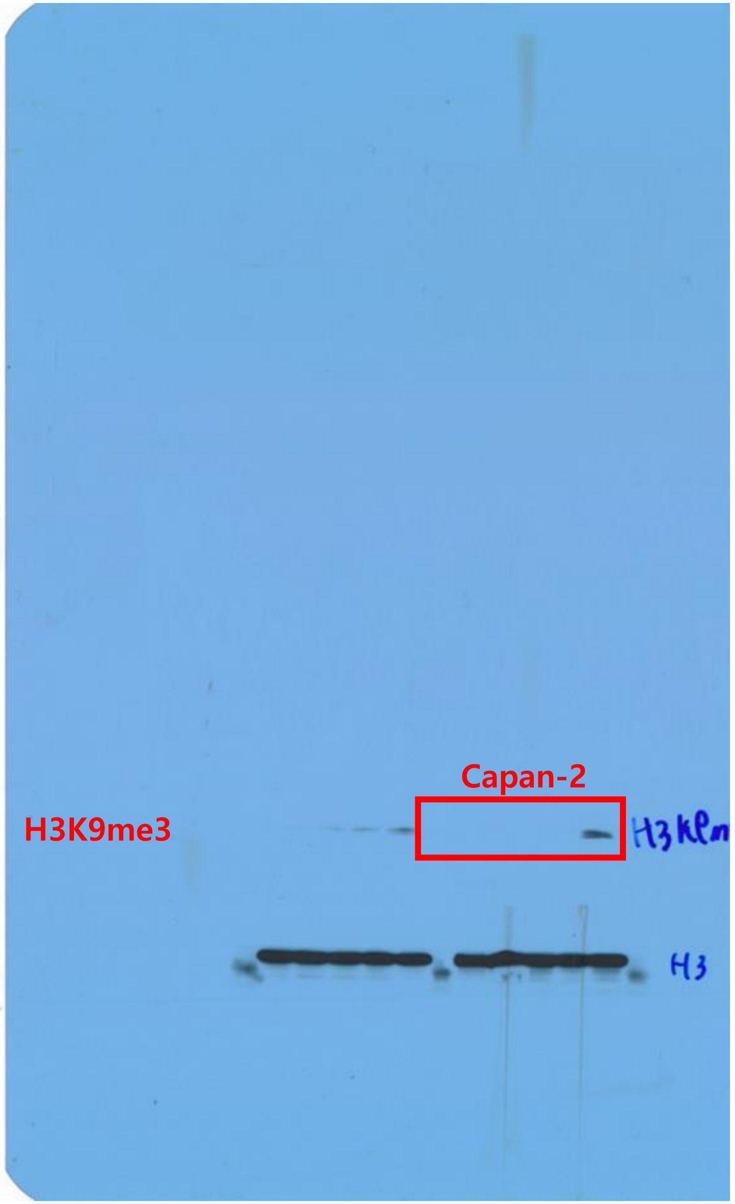

Figure 4F

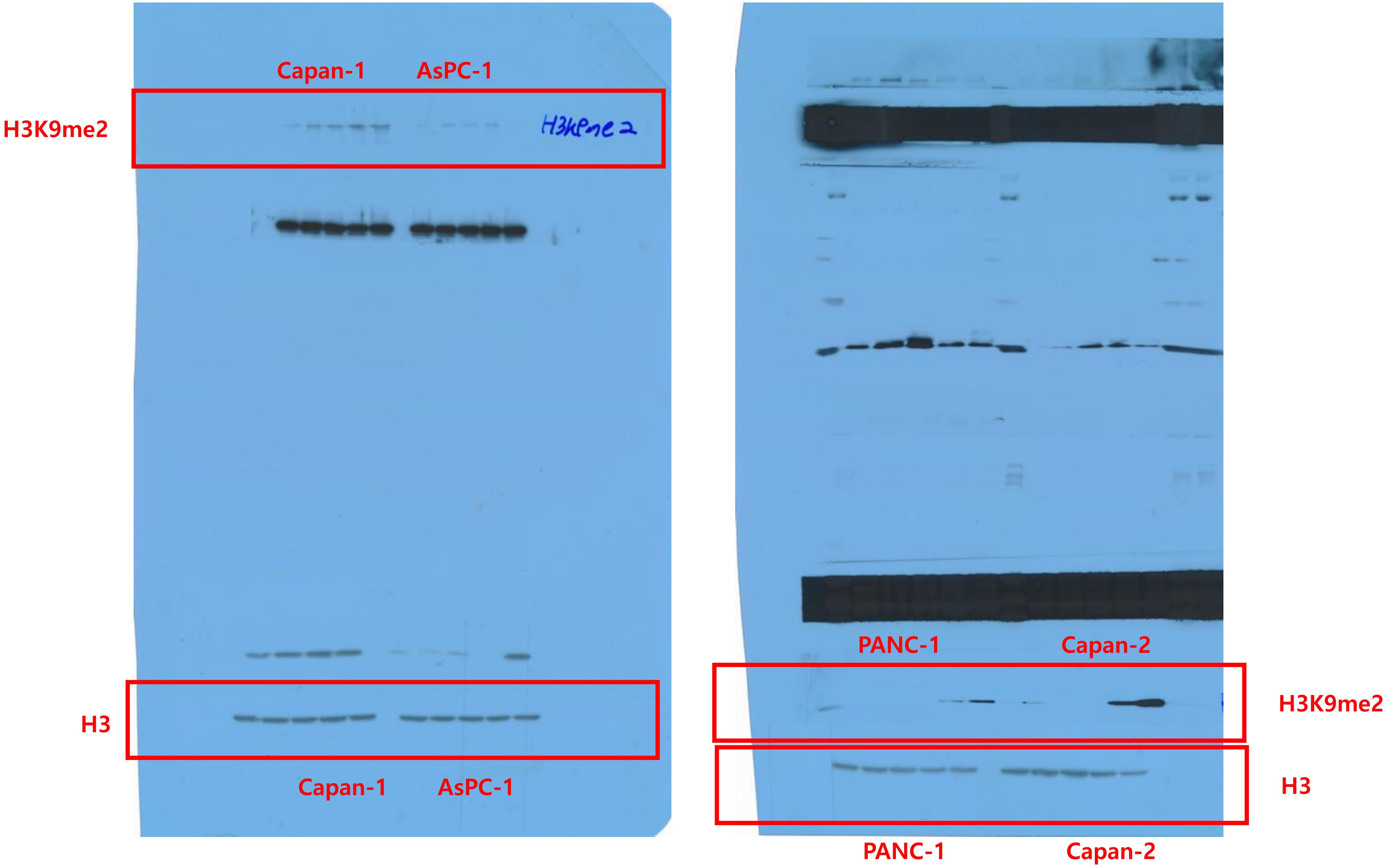

Capan-1
AsPC-1
H3K9me2
H3K9me2
H3
Capan-1
AsPC-1
PANC-1
Capan-2
H3K9me2
H3
PANC-1
Capan-2

Figure 4G

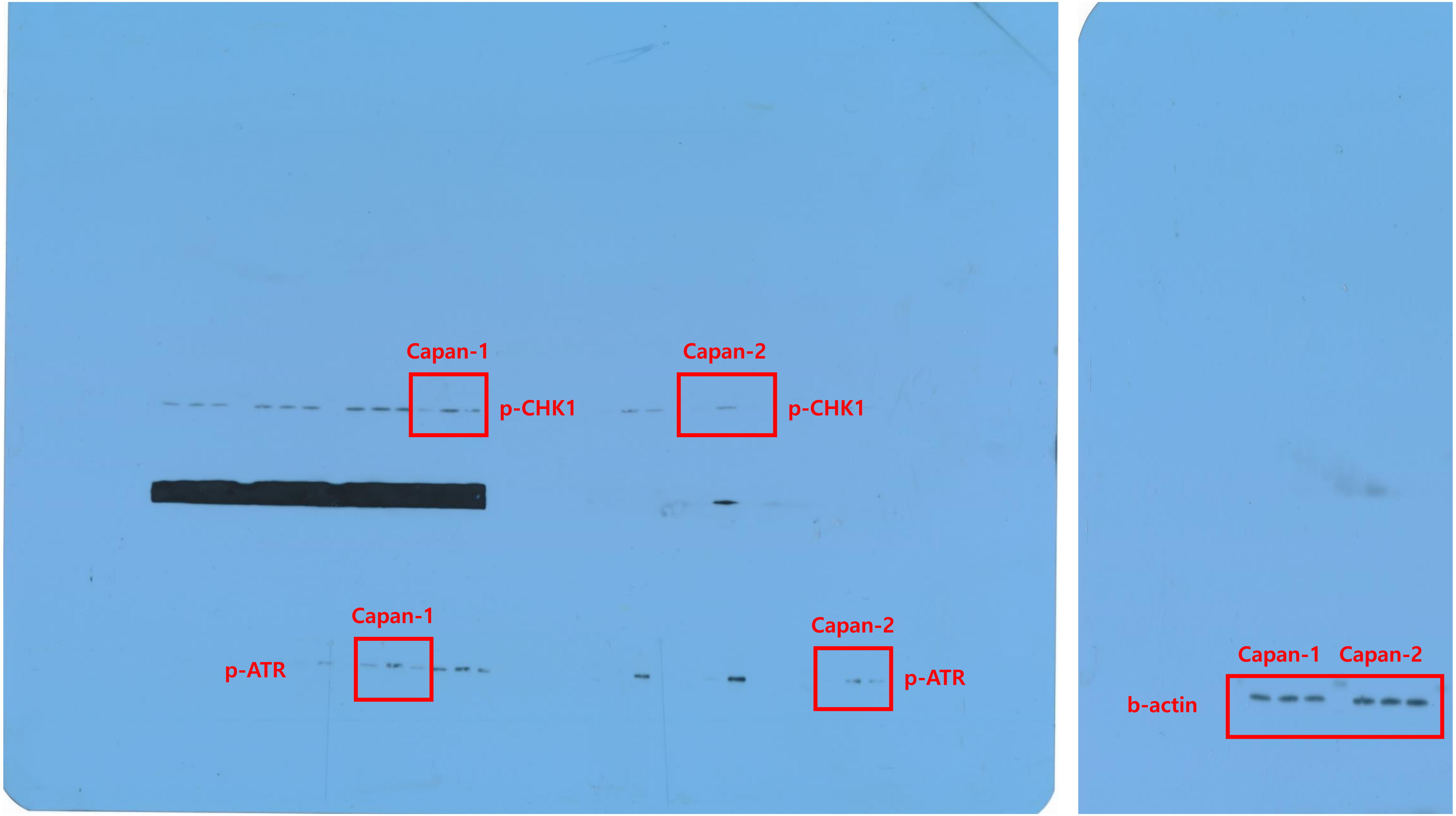

Capan-1
Capan-2
p-CHK1
p-CHK1
Capan-1
Capan-2
p-ATR
p-ATR
Capan-1
Capan-2
b-actin

Figure 5C

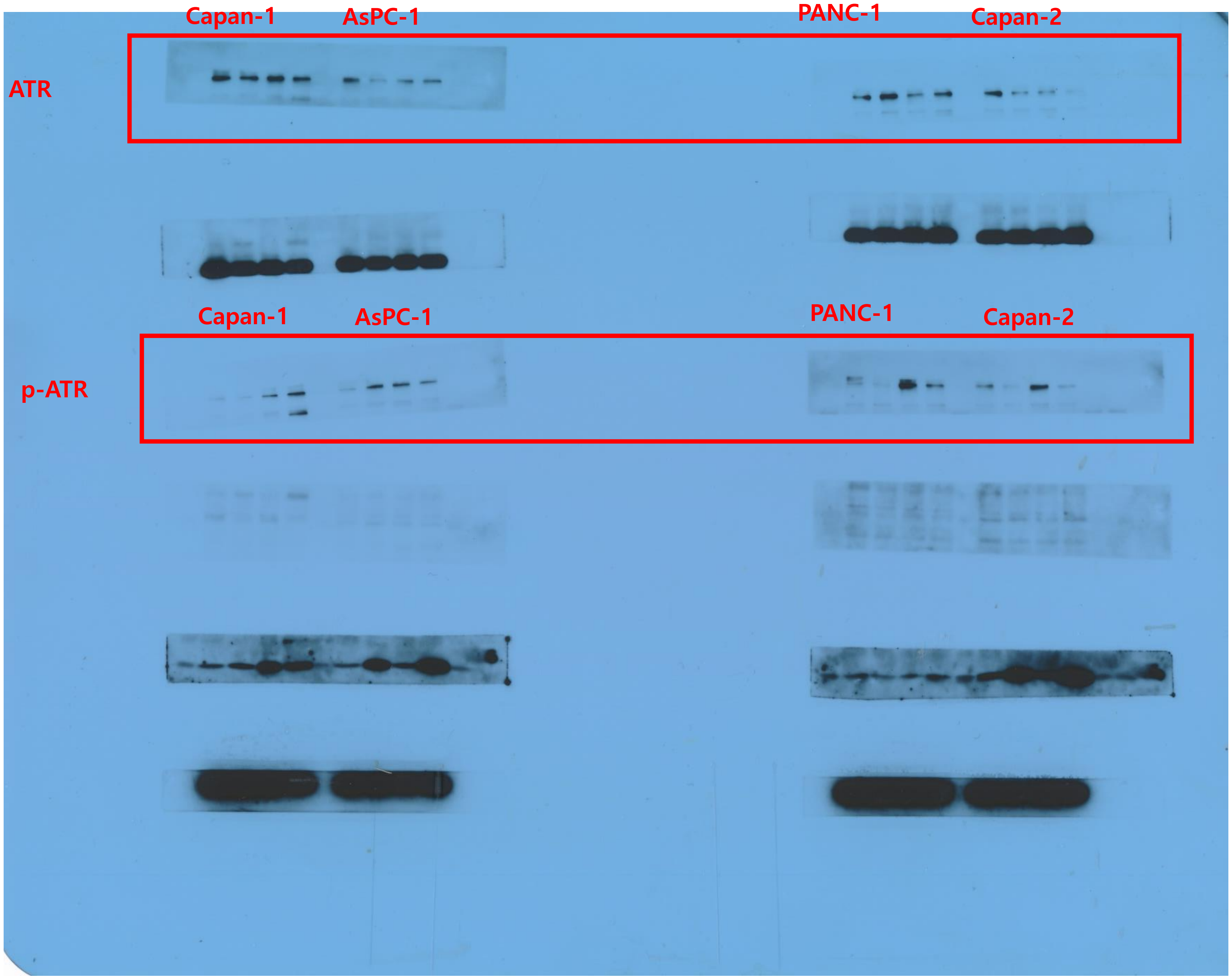
Capan-1
AsPC-1
PANC-1
Capan-2
ATR
Capan-1
AsPC-1
PANC-1
Capan-2
p-ATR

Figure 5C

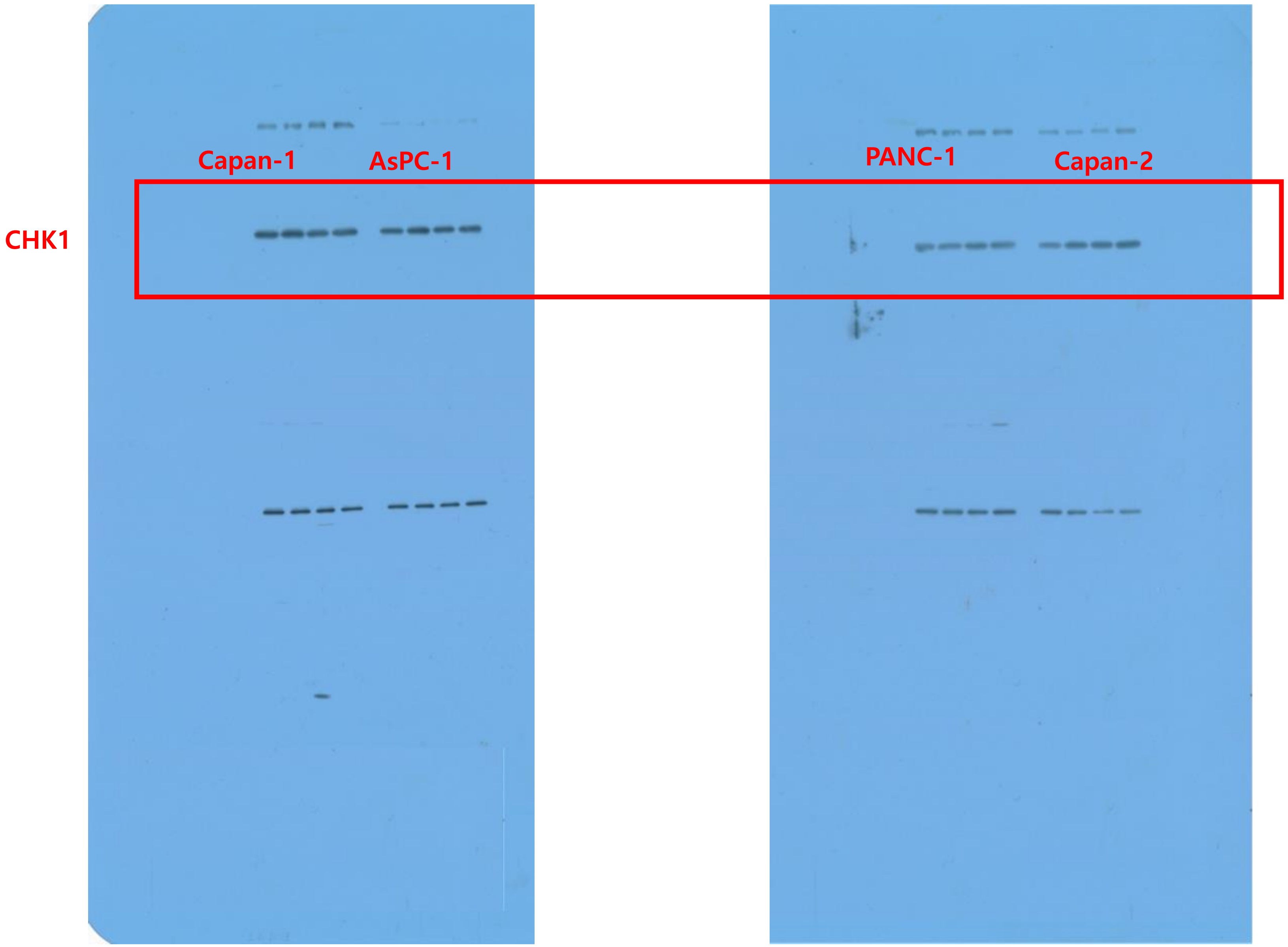

Figure 5C

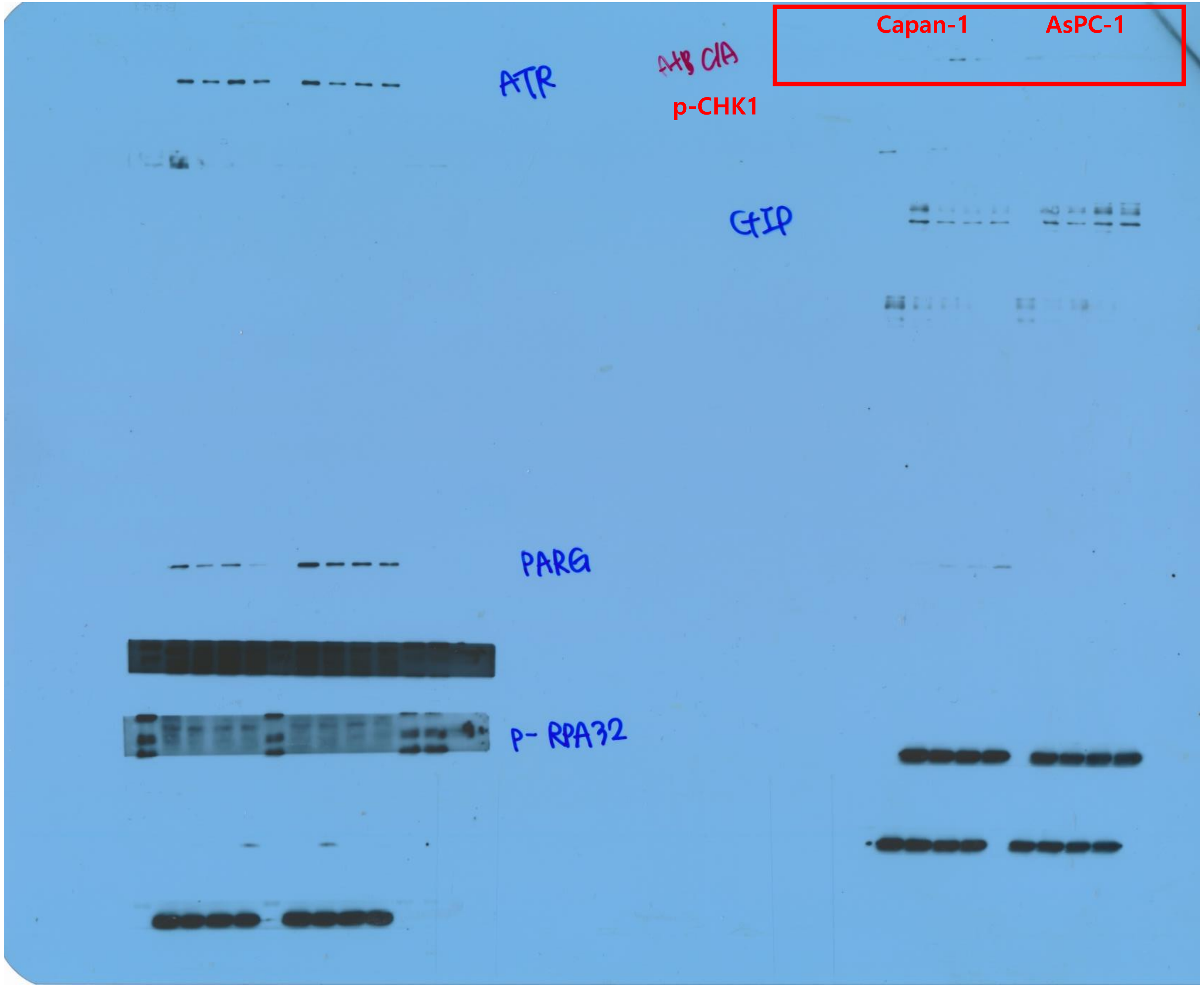

Figure 5C

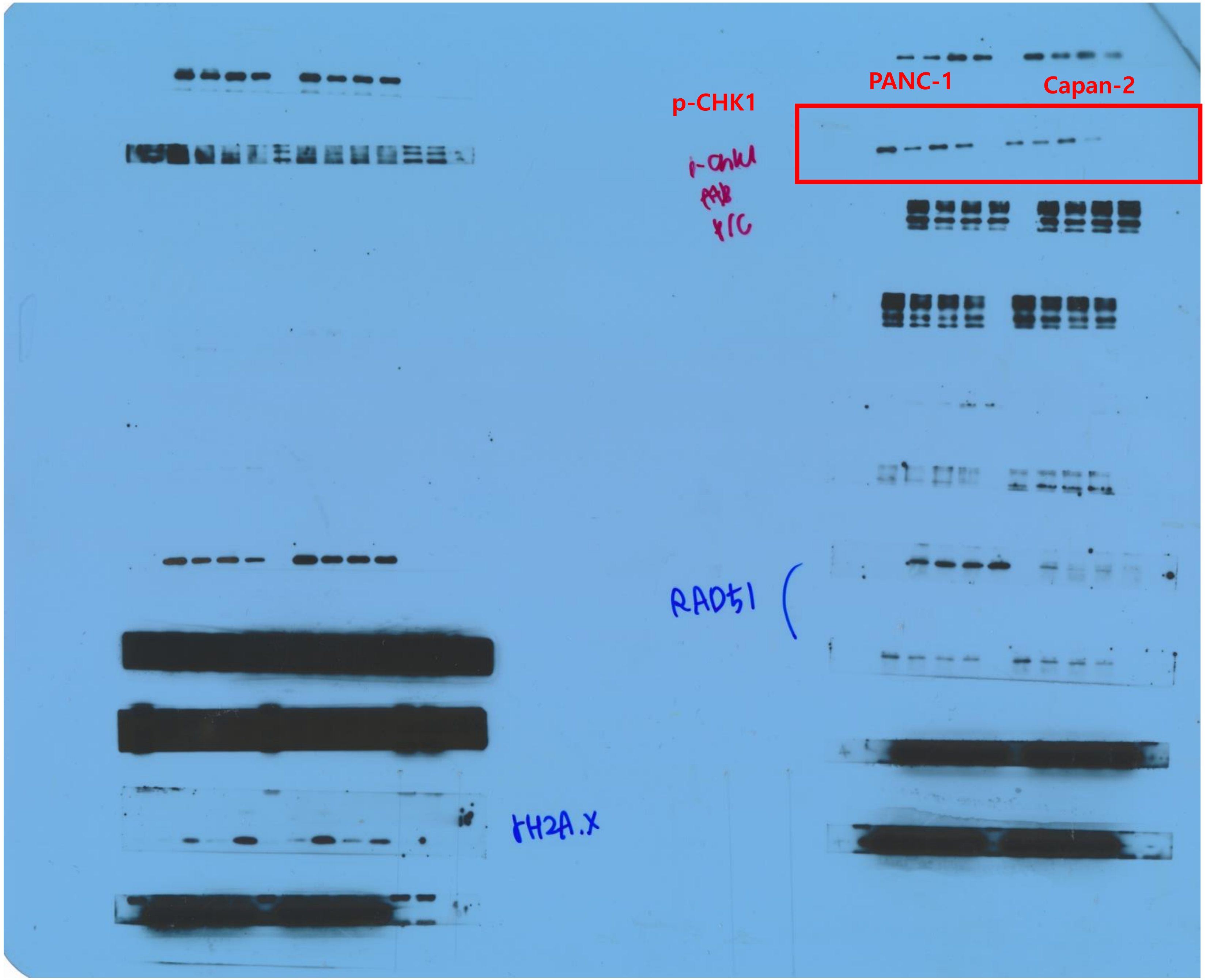

Figure 5C

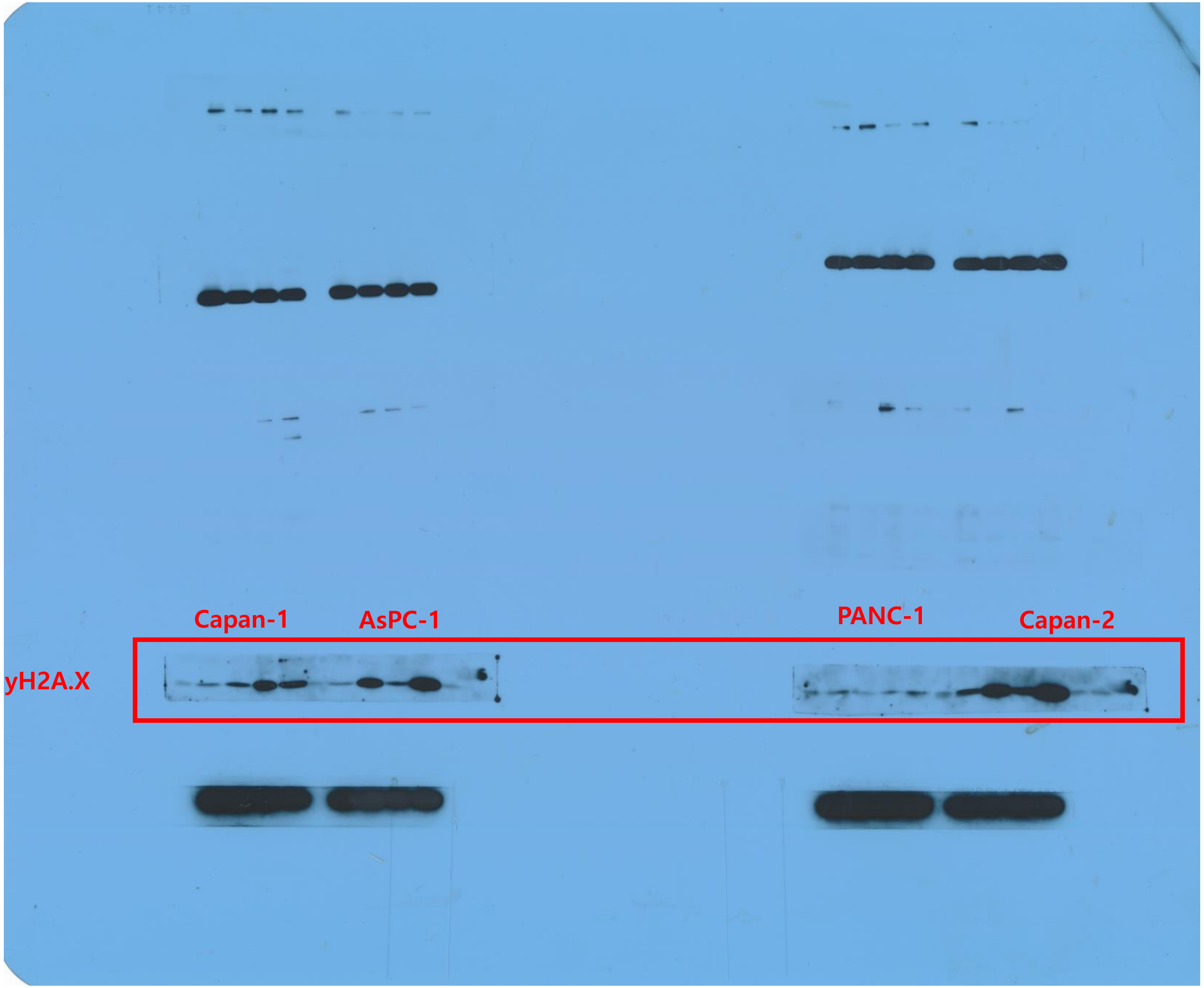

Figure 5C

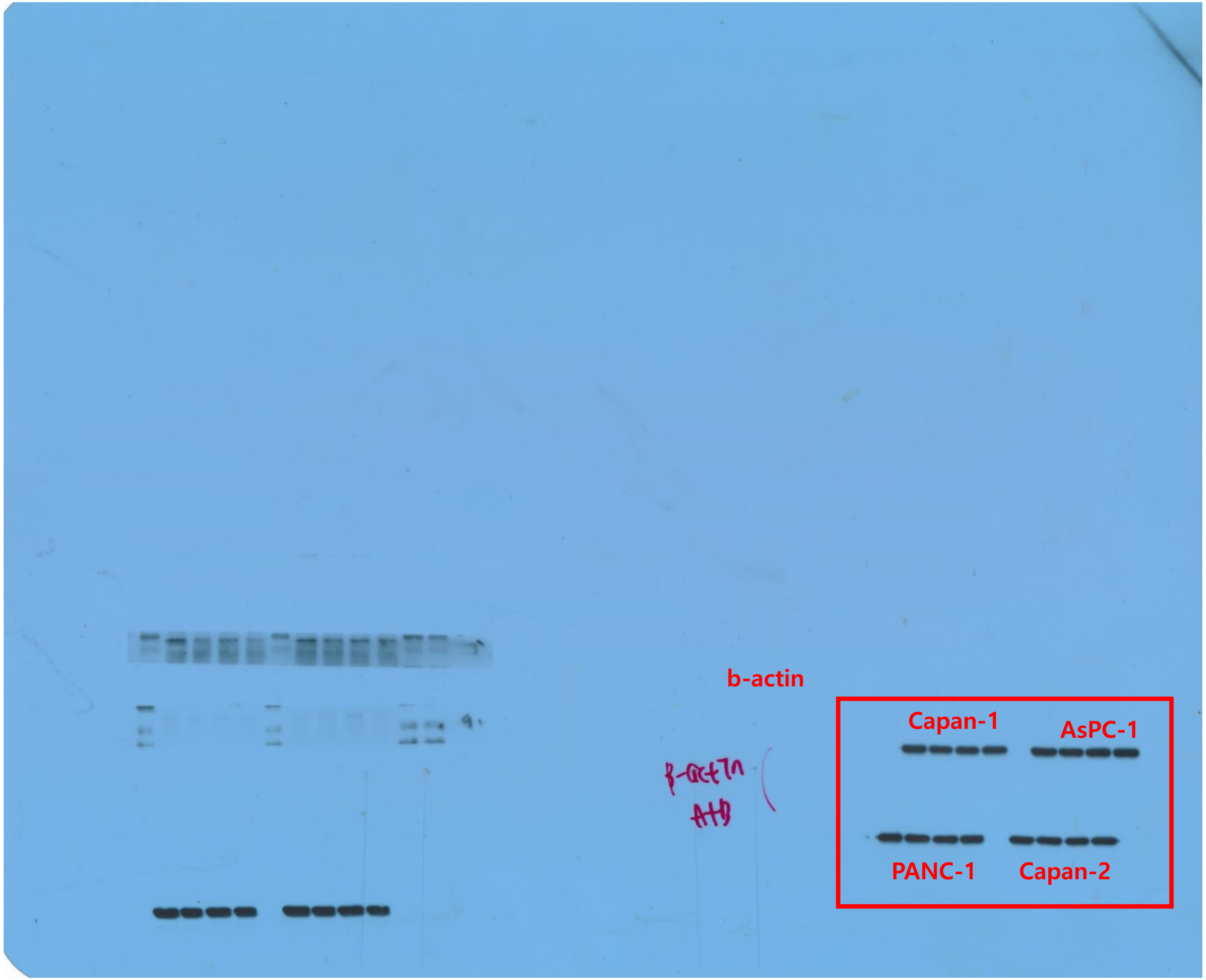

Figure 5E

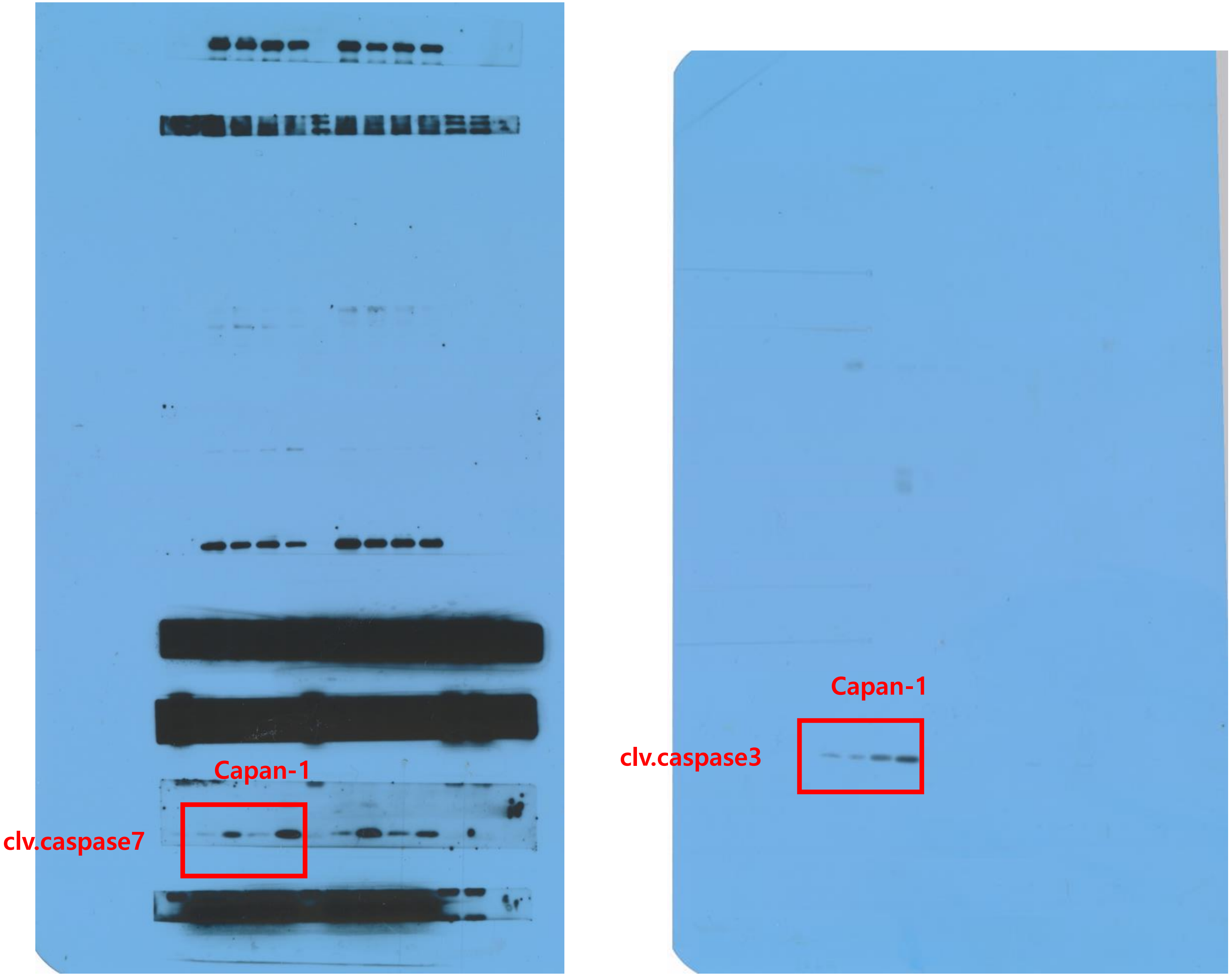

Figure 5E

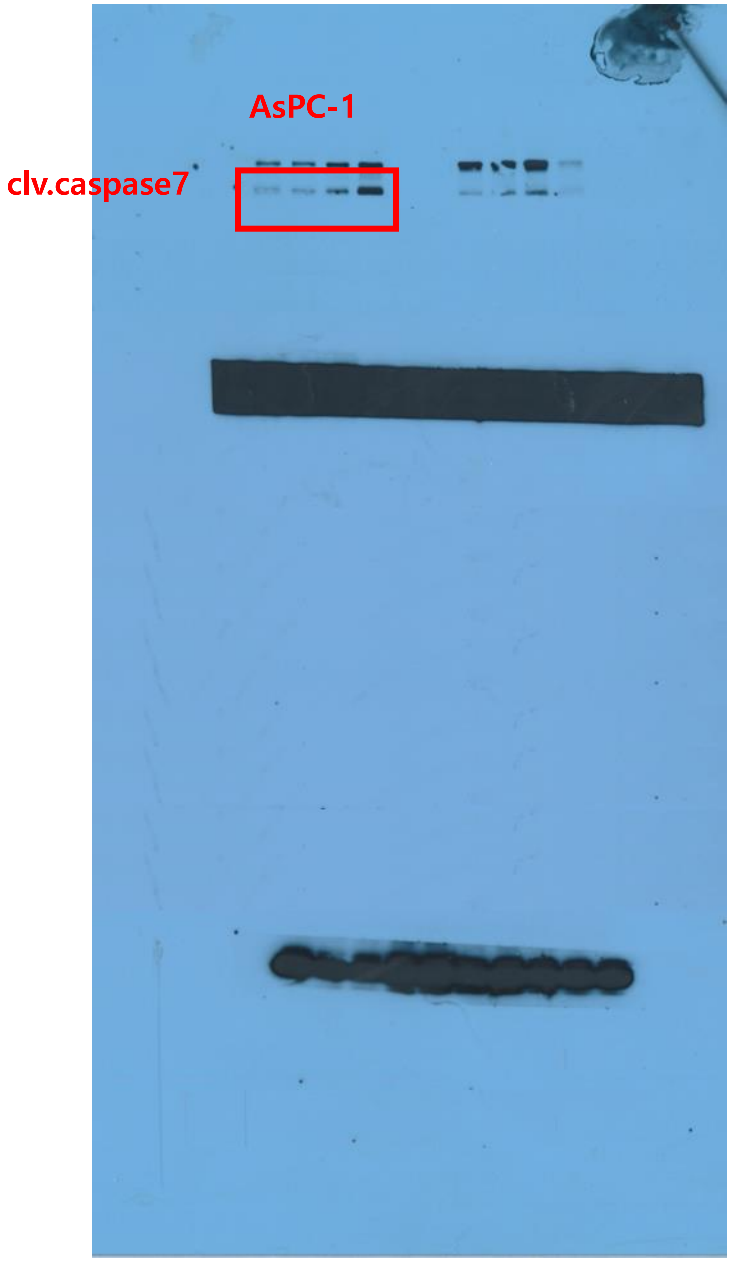

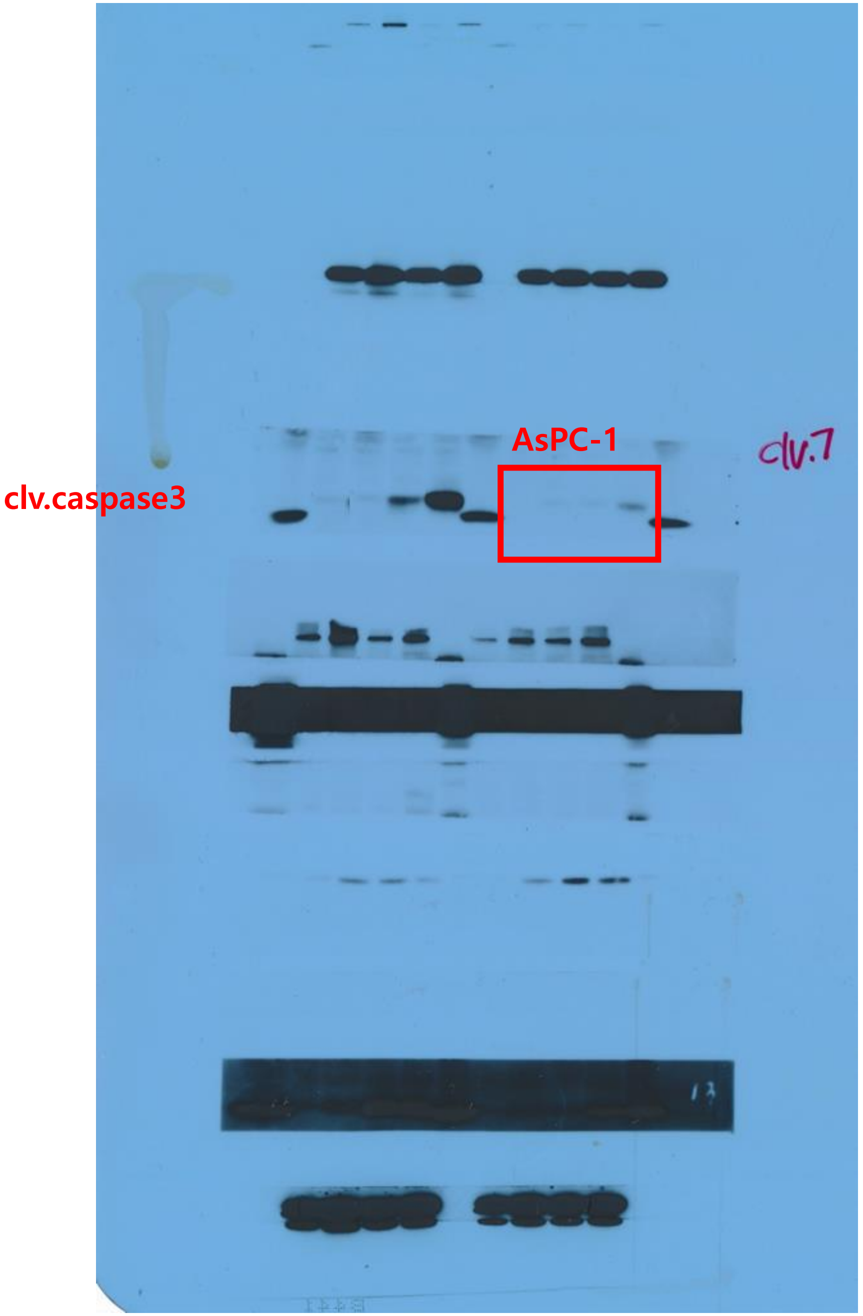

Figure 5E

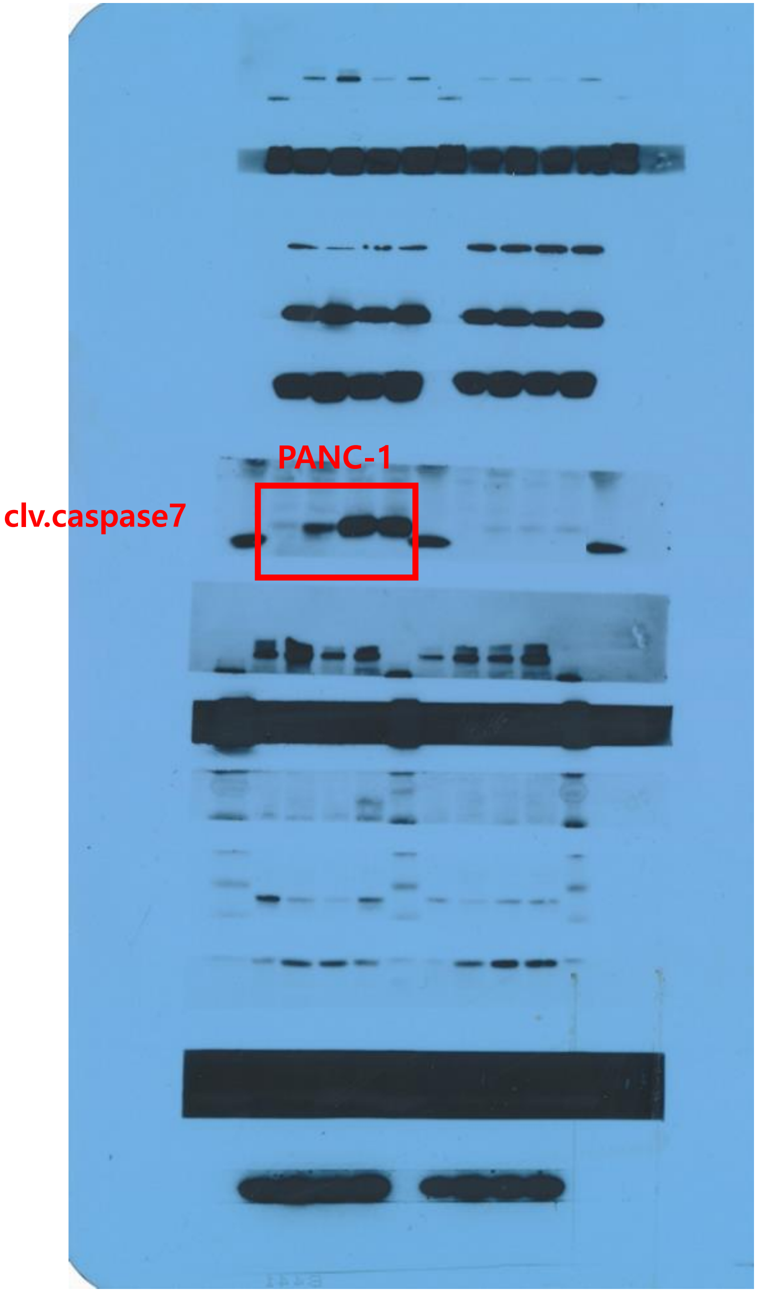

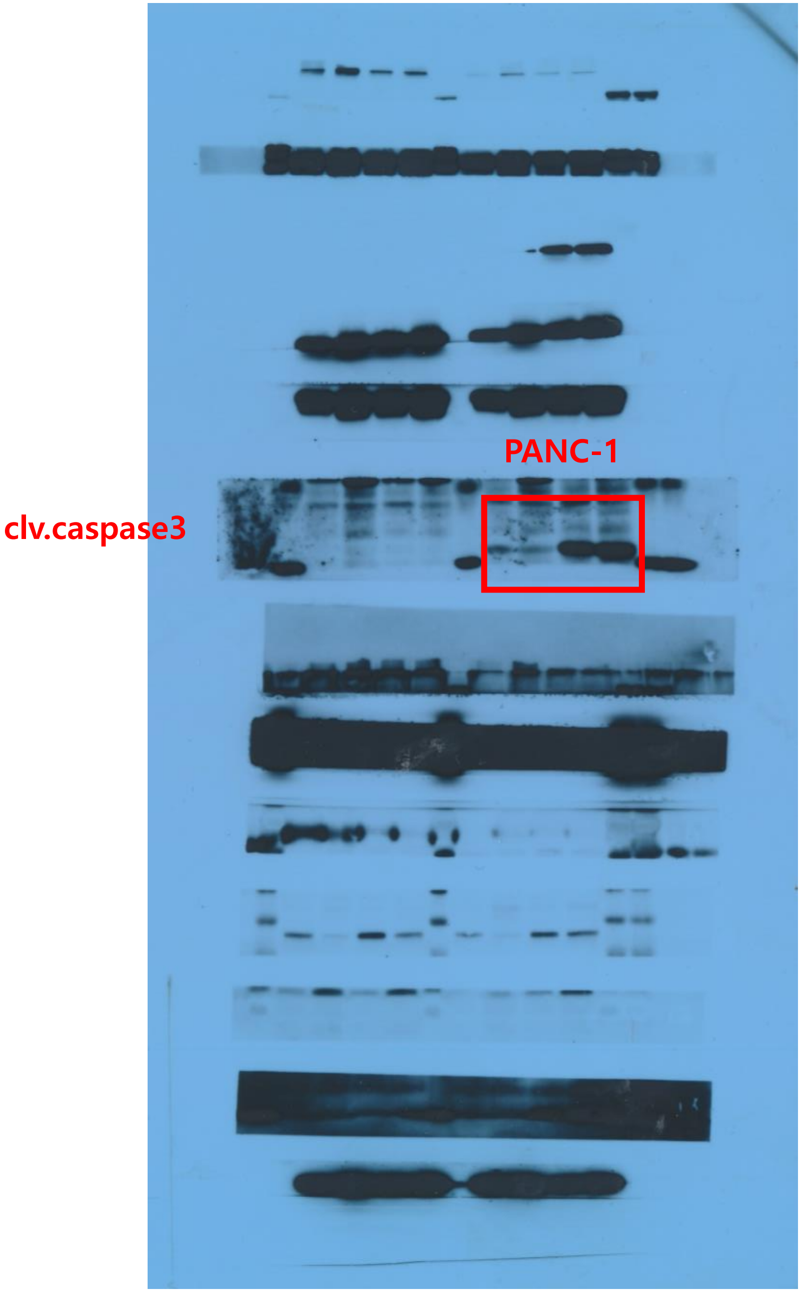

Figure 5E

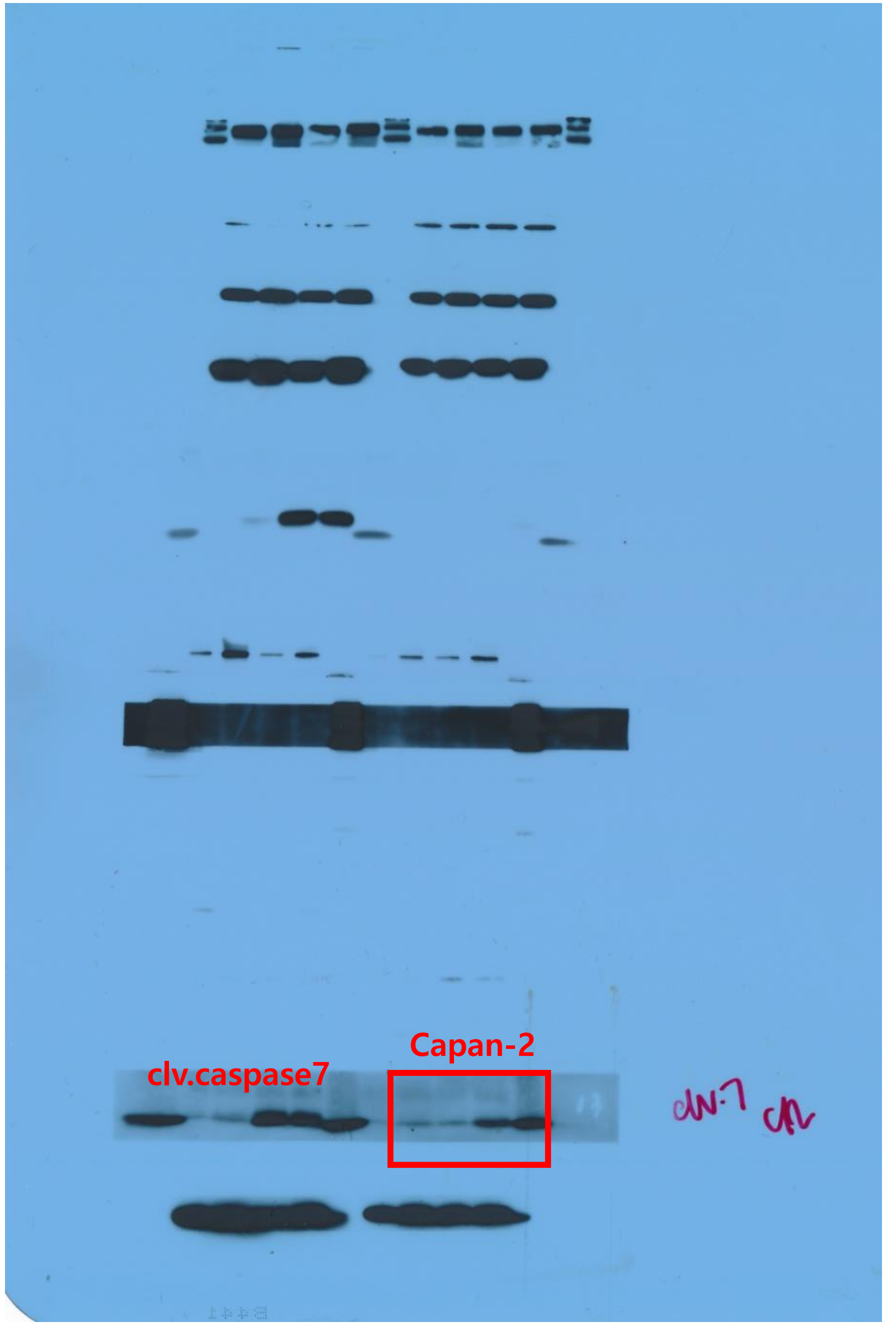

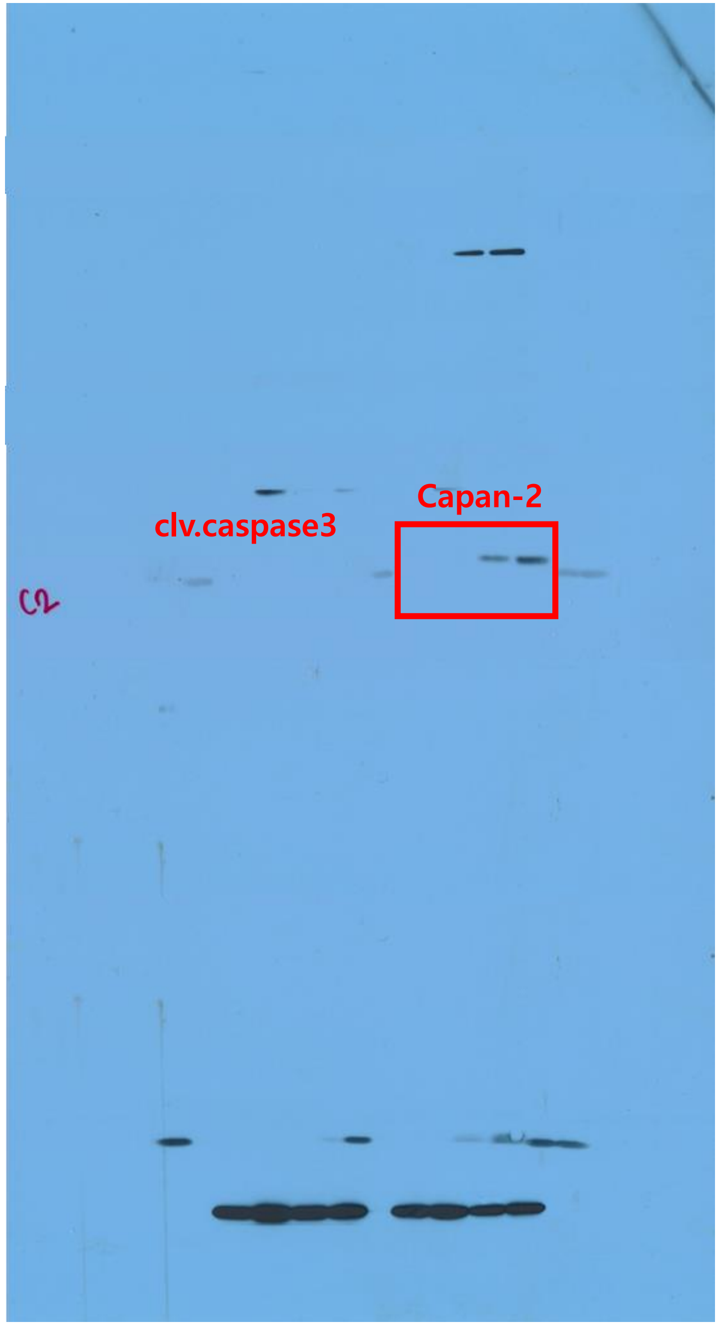

Figure 5E

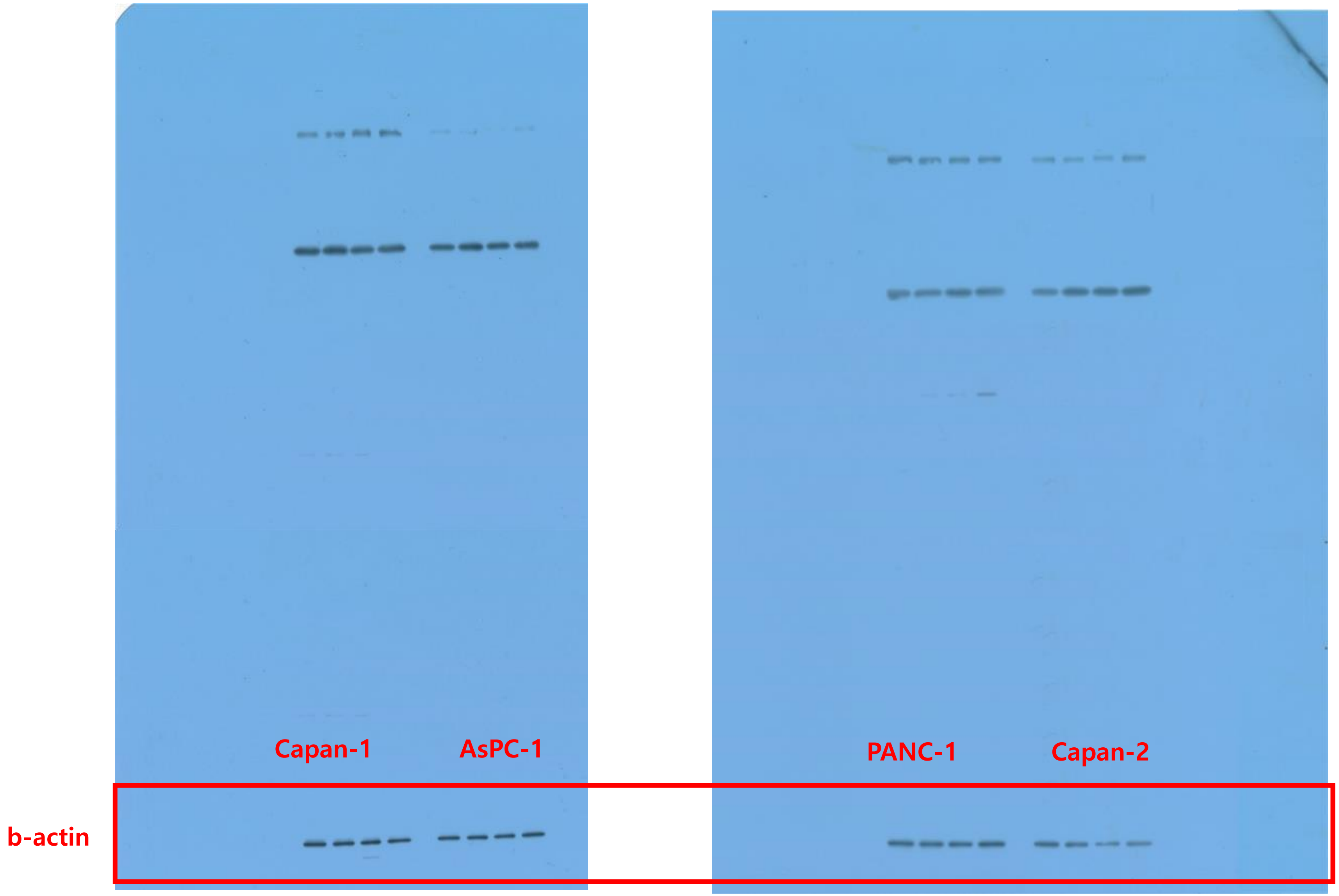

Supplement: Supplementary file 2 — Supplementary Material 2 [file 12964_2025_2242_MOESM2_ESM.pdf]
